# Trimodal, uncertainty-guided whole-slide framework for genome-scale spatial expression and image-only virtual perturbation in cancer cohorts

**DOI:** 10.1186/s13073-026-01713-y

**Published:** 2026-07-14

**Authors:** Zijun Wang, Chongyi Yang, Xiaoya Tang, Enzhi Yin, Yuxin Yao, Yuejun Luo, Jie He, Nan Sun

**Affiliations:** 1 Department of Thoracic Surgery, National Cancer Center/National Clinical Research Center for Cancer/Cancer Hospital, Chinese Academy of Medical Sciences and Peking Union Medical College, Beijing, 100021 P.R. China; 2 State Key Laboratory of Molecular Oncology, National Cancer Center/National Clinical Research Center for Cancer/Cancer Hospital, Chinese Academy of Medical Sciences and Peking Union Medical College, Beijing, 100021 P.R. China; 3 National Cancer Center/National Clinical Research Center for Cancer/Cancer Hospital, Chinese Academy of Medical Sciences and Peking Union Medical College, Beijing, 100021 P.R. China; 4 Department of Orthopaedic Oncology Surgery, Beijing Jishuitan Hospital, Capital Medical University, Beijing, 100035 China; 5 National Center for Orthopedics, Beijing, 100035 China; 6 Beijing Research Institute of Traumatology and Orthopaedics, Beijing, 100035 China

**Keywords:** Spatial transcriptomics, Digital pathology, Whole-slide images, Multimodal models, Uncertainty estimation, Mixture of experts, Virtual perturbation, Genome-wide expression

## Abstract

Spatial transcriptomics is powerful but costly; hematoxylin and eosin (H&E) images are routine. We present Coladan-human3K, the largest human spatial transcriptomics resource (~ 3,000 profiles), and Coladan, a trimodal (image, language, spatial-gene) whole-slide framework predicting genome-wide genes per spot with calibrated uncertainty while preserving foundation-model representations. Across 32 Visium datasets, Coladan improves Pearson correlation from 0.230 to 0.431 (~ 1.9 ×), shows pathway-level enrichment consistency, and transfers zero-shot to VisiumHD and spot-level Xenium. Classification token (CLS) embedding-only perturbation performs on par with expression-based baselines, enabling image-only virtual perturbation without measured expression, illustrated on normal and cancer prostate sections for in-situ hypothesis generation.

**Supplementary Information:**

The online version contains supplementary material available at https://doi.org/10.1186/s13073-026-01713-y.

## Background

Histopathology images are generated routinely in clinical settings, yielding vast archives of high-resolution morphological data [1, 2]. Spatial omics [3–10]—especially spatial transcriptomics—has transformed tissue biology by providing spatially resolved molecular readouts of cellular heterogeneity and microenvironmental interactions [11–13]. Yet current platforms are costly and low throughput [14, 15], which constrains sample sizes and hinders clinical deployment and large-scale translational research — thereby motivating image-based inference that scales with routine pathology.

Since the advent of spatial transcriptomics, researchers have attempted to predict spatial gene expression profiles directly from histopathology images [16–19]. More recent task-specific methods, such as HisToGene [18] and iSTAR [20], further illustrate this direct H&E-to-expression route by modeling spatial gene readouts from image patches. In parallel, pretrained H&E image foundation models [21–27] have provided increasingly strong visual representations for downstream pathology tasks. Building on these advances, several multimodal or representation-driven strategies have emerged. BLEEP [28] uses image–expression alignment and retrieval, PathOmCLIP [29] combines pathology and single-cell foundation representations for multimodal alignment, and DeepSpot [30] leverages pathology-pretrained backbones together with task-specific architectural refinement for spatial expression prediction. In addition, THREADS [31] extends multimodal alignment to bulk-level settings, and ROSIE [32] further explores related ideas for multiplex immunofluorescence protein prediction. Recently, large spatial omics collections such as HEST-1 k [33] and STimage-1K4M [34] have been assembled, providing broader training and evaluation resources for image-to-molecular modeling. Despite these advances, existing H&E-based expression inference still faces persistent limitations.

First, training datasets are small and often multi-species, limiting statistical power and relevance to human biology. Second, predictive accuracy is low and degrades sharply as the target panel scales to thousands of genes [29], yielding results of limited practical value. Third, most methods infer a fixed, small panel from isolated patches without whole-slide aggregation, failing to capture the broader transcriptomic context and losing information. Fourth, none of these models provide any quantitative uncertainty estimates, leaving users unable to judge which predictions to trust. Finally, they generalize poorly across spatial platforms and resolutions, constraining translational applicability.

To address these limitations, we curated Coladan-human3K, a dataset of 3,000 human spatial transcriptomic profiles with rich organ and disease annotations, and developed Coladan, a whole-slide, genome-scale dense prediction framework. Coladan consists of a patch encoder, a slide encoder, a language module and a gene decoder, each pretrained separately before undergoing end-to-end fine-tuning jointly across the language, image, and gene modalities. Notably, our gene decoder builds upon a pretrained single-cell RNA sequencing (RNA-seq) foundation model [35] via transfer learning to the spatial transcriptomics domain, allowing spatial prediction to build on single-cell representation priors while extending beyond fixed small-gene panels. On this backbone, we incorporate DeepSeekMoE [36], a mixture-of-experts (MoE) model together with Chain-of-Continuous-Thought [37] (Coconut) iteration yielding a CLS-driven iterative gene decoder that progressively reconstructs per-gene expression under whole-slide histological context. A novel training scheme enables the decoder to ingest a CLS token for iterative, per-gene expression generation, while a Gaussian negative log-likelihood loss provides gene-level uncertainty estimates for confidence-aware use.

Across 30 + standard 10 × Genomics spatial transcriptomics datasets, Coladan delivers substantial gains— ~ 1.9 × higher spot-wise Pearson than the strongest fine-tuned baseline. It shows stronger directional consistency in anchor-pathway enrichment and is the only method to generalize in zero-shot to VisiumHD (8 and 16 µm outputs) and Xenium-derived spot-level units. Enabled by our modular conditioning/training stack, a CLS-embedding-only perturbation setting performs on par with sequence-based state-of-the-art methods and, to our knowledge, constitutes the first image-only in silico perturbation at whole-slide scale without measured expression. The architecture further extends beyond the transcriptome by swapping task-specific decoders while retaining the image backbone and conditioning; integrating slide-level and spatial gene context yields a trimodal variant that modestly improves slide-level tasks. Calibrated aleatoric and epistemic uncertainty tracks error, enabling confidence-based filtering. Virtual prediction on The Cancer Genome Atlas (TCGA) slides suggests a possible route to scaling training with synthetic supervision. Together, these results establish the effectiveness, robustness, and versatility of our framework and suggest a route to reducing the time and cost of expression profiling for high-throughput research and clinical applications, while also enabling image-only in silico perturbation for hypothesis generation and targeted validation.

Coladan ingests a high-resolution H&E whole-slide image (~ 1 µm/pixel) and returns genome-wide spatial expression at each spot (64 × 64-pixel locus) together with calibrated (aleatoric and epistemic) uncertainty. The workflow also enables spot-selective image-only in silico perturbation, producing post-perturbation gene-by-spot matrices, and it preserves foundation-model representations so that task-specific heads can be fine-tuned for downstream slide-level tasks. Together, these outputs support retrospective analyses, hypothesis generation, aiding clinical decision-making, and screening/triage via confidence-based filtering and selective referral for sequencing. In oncology settings, archived H&E slides far outnumber spatially assayed samples, creating a gap for scalable molecular readouts. We therefore assess Coladan on multi-tumor benchmarks and illustrate cancer-focused case studies in prostate cancer using normal and tumor sections.

## Methods

### Study cohorts, datasets and preprocessing

This study analyzed only publicly available, de-identified human datasets (e.g., TCGA H&E whole-slide images; 10 × Genomics Visium). No new human participants were recruited and no interventions were performed. Ethics approval and informed consent were obtained by the original studies that generated these datasets.

#### Coladan-human3K

We assembled a large-scale, human-only spatial transcriptomics dataset (referred to as Coladan-human3K dataset). In total, Coladan-human3K comprises over 3,000 human samples annotated with diverse organ (e.g. breast, brain, prostate, colon, lung) and disease state, of which 75% include matching H&E whole-slide images, spot‐level coordinates and spatial gene-expression profiles. All data were drawn from publicly available database or published studies (e.g., Gene Expression Omnibus (GEO), 10 × Genomics published and other platforms; see Additional file 2) and span ten technologies: 10 × Genomics Visium (n = 2,440), CosMx (n = 42), GeoMx (n = 58), multiplexed error-robust fluorescence in situ hybridization (MERFISH) (n = 44), Slide-seq V2 (n = 82), Stereo-seq (n = 25), spatial transcriptomics (ST; n = 190), Xenium (n = 153), VisiumHD (n = 19) and electro-seq (n = 4). For the platform-level zero-shot evaluations reported in this study, VisiumHD and Xenium sections were set aside before model development and used only for held-out platform testing.

#### Gene-expression preprocessing

We filtered out genes detected in fewer than 5% of spots across the dataset. For each spot, we retained all nonzero (expressed) genes and randomly sampled zero-count genes in a number equal to 10–35% of that spot’s expressed gene count. This balanced sampling of expressed and non-expressed genes supports robust training. During input to the model, all nonzero expression values are discretized into 50 bins (Bin50), which reduces measurement noise and the influence of outliers, compresses the dynamic range of expression levels, and stabilizes training by having the model learn relative expression differences rather than exact counts. In practice, Coladan’s flexible decoder lets us supply every expressed gene for a spot and add up to 35% additional randomly sampled zero-expression genes. Including these extra zeros broadens the expression spectrum seen during training and empirically improves model learning.

#### Image preprocessing

We began with full-resolution H&E whole-slide scans. If a slide’s spot‐diameter metadata indicated a native spot size between 48 µm and 64 µm, the image was left at its original resolution (thereby offering robustness to resolution variability); otherwise, we resized the entire slide to yield an approximate scale of 1px/µm. For each spot coordinate, we then extracted a 224 × 224 px patch centered on that spot—this larger patch preserves sufficient morphological context while encompassing the central 64 × 64 px region whose gene expression is to be predicted. The patch coordinates within the down-sampled slide were recorded.

#### Training/validation split

To facilitate model training and hyperparameter tuning, Coladan‐human3K was randomly partitioned at the slide level: approximately 3% of the tissue sections—stratified to preserve representation of all organs and disease states—were held out as a validation set for early stopping and model selection, while the remaining 97% were used for training. No tissue section appears in both partitions, and all downstream test evaluations use entirely independent, held‐out datasets.

#### Test datasets (10 × Genomics Visium, Xenium and high-resolution VisiumHD)

For independent evaluation, we curated two additional spatial transcriptomic test sets. The first was a 10 × Genomics Visium test set consisting of multiple human tissue sections from the 10 × Genomics public datasets (breast (n = 9), brain/CNS (n = 6), prostate (n = 5), ovary (n = 3), colon/colorectum (n = 3), heart (n = 2), lymph node (n = 2), lung (n = 1) and uterus (n = 1)), totaling 32 slides were resized to similar spot size (~ 64 μm diameter) and gene panel as our training data. These slides were processed in the same manner as the training data (H&E image preprocessing and gene expression preprocessing). The second test set was a 10 × Genomics Xenium dataset, the Xenium test set comprised 11 human tissue sections curated from public datasets—lung (n = 8; 6 diseased, 2 healthy), prostate (n = 1; cancer), bowel/colon (n = 1; cancer), and skin (n = 1; cancer)—corresponding to IDs TENX158, TENX157, TENX147, NCBI882, NCBI856, NCBI884, NCBI873, NCBI866, NCBI865, NCBI857, and NCBI875 (processed details see HEST-1k [33]). For Xenium, native transcript measurements were converted into processed spot-level target units. These processed units, rather than native cell polygons, were used as the measured targets for evaluation. The third test set was a high-definition spatial transcriptomics dataset from the 10 × Genomics public datasets, denoted VisiumHD, characterized by a much finer spatial resolution. This dataset (colon/colorectum (n = 6), lung (n = 5), breast (n = 3), head & neck (n = 2), kidney (n = 1) and pancreas (n = 1)) challenges the model’s ability to generalize to different spatial platform. To make the VisiumHD data compatible with our model (which was trained on ~ 64 μm spots), we selected the “square_016µm” output layer from VisiumHD as our test set, which yields full-resolution spot diameters of approximately 60 µm at 20 × magnification. For these slides, we applied H&E image preprocessing and gene expression preprocessing in the same manner as the training data. This ensured that the input distribution (in terms of patch size and gene counts per spot) was comparable to the training conditions. We also evaluated the finer “square_008 µm” layer to probe resolution robustness, and observed that Coladan’s predictive performance remained stable, confirming strong cross‐scale generalization.

#### Perturb-seq datasets for zero-shot perturbation analysis

We evaluated Coladan on four independent single-cell Perturb-seq resources that report genome-scale transcriptional responses to defined genetic edits, treating them purely as out-of-distribution test sets (no model re-training). The Adamson dataset contains perturb-seq in the K562 leukaemia cell line [38]. After standard quality control (QC), guide filtering and log-normalization, we retained 87 unique perturbation conditions, each represented by the mean expression vector of its constituent cells. The Norman dataset contains perturb-seq in the K562 leukaemia cell line [39]. Following identical processing, 283 distinct activation conditions were obtained. The Replogle dataset [40] applies compact, multiplex CRISPR interference (CRISPRi) screening to two cell lines: K562 (chronic myeloid leukaemia) and RPE1 (retinal pigment epithelium). We processed the two cell-line subsets separately, yielding 2,057 K562 conditions and 2,393 RPE1 conditions, from raw count matrix. Within each perturbation condition we average-pooled single cells to form a “pseudo-spot” vector. Altogether 4,820 condition-level pseudo-cells underpin the zero-shot perturbation experiments.

#### Single-cell protein dataset for fine-tuning

The single-cell proteomics by mass spectrometry (SCoPE2) monocyte-to-macrophage single-cell proteomics dataset (1,490 cells; 3,042 proteins) [41]. We applied the same gene-expression preprocessing pipeline as for the RNA data. During fine-tuning, the shared patch and slide encoders remained frozen, and only the CLS-driven decoder was updated to predict protein abundance. This adaptation demonstrates that our architecture can be readily extended from spatial transcriptomics to spatial proteomics without modifying the core image encoders, and can leverage the vast quantities of single-cell–level data to train new gene decoders.

#### CODEX multiplexed imaging (protein expression) dataset

To probe cross-omic transfer, we used a public CODEX multiplex-imaging dataset [42] and chose human glioblastoma samples with ~ 20 protein markers per cell. Fluorescence stacks were rigidly aligned to the accompanying bright-field/H&E reference via oriented FAST and rotated BRIEF (ORB) features [43] and random sample consensus (RANSAC) [44], then resampled to 1 µm px⁻^1^ to match the scale of our training slides. A regular 64 µm square grid (≈ 64 px) was rasterised over the tissue mask; tiles with < 20% tissue were discarded. For each retained tile we averaged the non-zero fluorescence of every marker, producing a spot-by-protein matrix. Marker intensities were z-score normalised across the slide. The resulting pseudo-spots, together with the aligned H&E image, were fed to Coladan for test.

#### TCGA histology dataset for virtual profiling

To explore Coladan as an in silico spatial‐omics engine for routine pathology, we harvested diagnostic H&E whole-slide images from The Cancer Genome Atlas (TCGA). Ten slides were randomly drawn from each of tumor types (or all available slides if fewer than ten), then a final stratified sample of 50 slides was retained for inference. Digital slides were inspected for quality and their native physical pixel size, measured in micrometres per pixel (mpp), extracted from image metadata. All TCGA slides were uniformly rescaled to 1 µm px⁻^1^ resolution prior to grid extraction. Large blanks and artifacts were culled with an adaptive tissue mask that combines Gaussian‐thresholded luminance with Canny edge sparsity. A 64 µm square grid was then rasterised across tissue regions (centre-to-centre step = 64 px in the working resolution). For every accepted grid point we recorded (i) the 64 × 64 px “spot” bounding box, (ii) a 224 × 224 px context patch centered on the spot (identical preprocessing pipeline as for training images), and (iii) Cartesian and physical coordinates. Two matched slide versions were exported per case: a native-resolution and a 1 µm version. These 50 slide TCGA sets serve exclusively for the “virtual clinical deployment’’ experiment. Based on our uncertainty estimates, the virtual predictions achieve high confidence scores, supporting the reliability of the inferred spatial expression maps.

### Model architecture

#### Coladan

Coladan is a two‐stage trained, trimodal framework that excels at inferring spatial gene expression from histology images. It comprises four major components (Additional file 1: Fig. S1): a patch encoder that extracts localized feature embeddings from overlapping 224 × 224 px tiles centered on each 64 × 64 px spot; a slide encoder that integrates information across the entire tissue slide, produce slide-aware patch embeddings and a whole-slide representation; a frozen MedGemma [45] model kept frozen during training and used only as a feature extractor; and a CLS‐driven gene decoder whose original feed-forward sublayers are replaced by mixture-of-experts blocks, and genome-scale expression is generated through Chain-of-Continuous-Thought iterative embedding reconditioning mechanism with uncertainty-aware prediction heads.

#### Image-to-expression inference pathway

Coladan uses a local-to-slide-to-CLS-to-gene pathway for image-conditioned spatial expression prediction. For each target locus, a 224 × 224 px H&E patch centered on the 64 × 64 px prediction region is extracted from the whole-slide image. The patch encoder converts this local crop into a morphology embedding. The slide-level encoder then processes patch embeddings together with their spatial coordinates, allowing each local representation to be contextualized by other regions of the same slide. This produces slide-aware locus embeddings rather than isolated patch embeddings alone. For the target locus, the corresponding slide-aware embedding is passed through the projector and mapped into the 512-dimensional CLS space of the gene decoder, yielding an H&E-derived locus-CLS representation. For gene prediction at this locus, the H&E-derived CLS representation is provided to the gene decoder together with the queried gene identities. Each queried gene is represented by its gene-ID embedding, while its expression embedding is replaced by a MASK token because measured expression is unavailable during image-conditioned inference. The CLS-conditioned gene decoder processes these masked gene queries with Transformer layers whose feed-forward blocks are replaced by fine-grained mixture-of-experts modules. The decoder is applied within Coconut iterative decoding, where intermediate hidden representations from one pass are reused in subsequent passes, ultimately producing predicted expression values for the requested genes at the target locus. Predictive uncertainty is computed by combining aleatoric variance estimated by the Gaussian-NLL head with epistemic variance estimated from variability across repeated Coconut inference passes.

#### Patch encoder

The patch encoder divides the whole‐slide image into small, fixed‐size regions and transforms each patch into a compact, high‐dimensional feature embedding which captures local tissue morphology and texture. Coladan’s patch encoder is built on GigaPath [23] patch encoder, a vision transformer foundation model for pathology image patches which was pretrained on millions of H&E image patches to learn generalizable histology features. In Coladan, we fine‐tune this pretrained ViT backbone to capture local context by extracting 224 × 224 px patches centered on each 64 × 64 px spatial spot and mapping them to 1536-dimensional feature embeddings, these patch embeddings are then fed into the slide encoder, which integrates information from all patches so that each local embedding is enriched with information from the entire slide.

#### Slide encoder

The slide encoder of Coladan is a transformer-based module built on the GigaPath [23] slide encoder backbone. It takes as input the set of high-resolution patch embeddings along with their spatial coordinates and produces two outputs: (i) a context-rich whole-slide representation that captures global tissue architecture, and (ii) updated, slide-aware patch embeddings (768-dimensional each) in which each local feature is enriched by information from its surrounding patches. The former is used for slide-level downstream tasks such as prognosis prediction, disease classification, and clustering, while the latter serves patch-level tasks—most notably our spatial gene-expression inference. During Coladan’s training, we further align this visual context with language by incorporating the pretrained language model from MedGemma [45].

#### Projector module

To harmonize the 768-dimensional slide encoder outputs with the gene decoder’s 512-dimensional CLS embedding space, we employ a compact three-layer projector network. First, a linear layer expands the feature dimension from 768 to 2048, followed by Layer Normalization and a LeakyReLU activation to stabilize and enrich the representation. A second linear layer then maintains this 2048-dimensional space and applies another LeakyReLU nonlinearity to introduce additional modeling capacity. Finally, a 2048 → 512 linear mapping projects the features directly into the gene decoder’s CLS embedding space. This sequential design—dimensional expansion, normalization, and non-linear transformation—ensures a smooth, high-fidelity transfer of visual context into the gene decoding module.

#### CLS-driven gene decoder

The gene decoder is a 12-layer transformer adapted from scGPT [35], a foundation model originally pretrained on huge amount of single-cell RNA-seq data. We retain the scGPT-style gene-token formulation and adapt the model to the spatial transcriptomics domain with several modifications. First, we replaced each feed-forward sublayer with a fine-grained mixture-of-experts (MoE) design that incorporates shared-expert isolation. Second, we implement cross-flash attention [46] within the attention layers to accelerate both training and inference. Third, we also overhaul the standard generation scheme by employing the Coconut (Chain-of-Continuous-Thought) iterative inference reconditioning mechanism (Additional file 1: Fig. S1), which enables progressively get per-gene predictions. Fourth, a Gaussian negative log-likelihood regression head is added on top of each output token to jointly predict both the mean expression level and its associated uncertainty. In Coladan, the CLS token serves as the compact conditioning representation for a target locus: during Stage 1, scGPT is adapted to spatial transcriptomics and converted into a CLS-driven gene decoder by learning an expression-derived CLS from measured spatial transcriptomic profiles and using this CLS representation to reconstruct masked or queried gene-expression values; during Stage 2.1, the expression-derived CLS generated by the Stage 1 decoder serves as the alignment target for the projected H&E-derived locus-CLS; and during Stage 2.2 and inference, the gene decoder requires only the H&E-derived CLS representation; the decoder outputs predicted expression values for the requested genes at the target locus.

For clarity, the gene decoder can receive three types of information: queried gene identities, a CLS representation and gene expression inputs. Across training and inference, queried gene identities are always provided. A CLS representation is not externally provided when generating the expression-derived CLS from measured spatial transcriptomic profiles in Stage 1 and Stage 2.1; otherwise, the decoder is conditioned by either an expression-derived CLS or an H&E-derived locus-CLS. Gene expression inputs are provided only when generating the expression-derived CLS from measured spatial transcriptomic profiles in Stage 1 and Stage 2.1; otherwise, measured expression is not provided and expression inputs are replaced by MASK tokens.

#### Gene decoder embedding

Within the gene decoder, each queried gene is represented by two learned embeddings that are summed to form its input feature. First, the gene’s identifier is mapped through a vocabulary lookup to produce a gene-ID embedding. Second, its observed expression value is tokenized into one of 50 discrete bins and similarly embedded as a gene-expression embedding. Adding these two vectors yields a 512-dimensional input embedding for each gene–expression pair. Gene expression can be represented as a MASK token, which means the model is not provided with that gene’s expression information.

Upon processing the sequence of input embeddings, the decoder outputs a corresponding set of output embeddings (one per gene) plus a special 512-dimensional CLS embedding that aggregates information across all genes. Each output embedding then feeds into two separate linear “heads”: one predicts the gene’s expression value, and the other predicts an uncertainty estimate for that prediction. In this way, genes whose gene-expression were masked at inference time can receive a predicted expression (from the output-value head) along with a confidence score (from the uncertainty head), effectively letting the model impute missing values based on the patterns learned from other genes and capture intrinsic relationships among them.

#### CLS inference pathway

At inference time, to predict any given gene, we provide the gene decoder model with the CLS embedding—either directly from the projector module or carried forward from the previous Coconut iteration—and mask part or all gene expression as “MASK” tokens. The decoder then outputs the masked gene’s expression and its uncertainty purely from the contextual information encoded in the CLS token. Note that this is not the standard “decoder” architecture used in large language models but an analogous, transformer-based information-decoding module tailored for gene expression inference.

#### Flexible genome-scale prediction

Coladan’s gene decoder supports dynamically querying any arbitrary set of genes at both training and inference, users can specify exactly which genes to predict simply by inputting their gene-ID without architectural changes. You can specify a maximum input length (e.g. set to 5,000 in evaluation); shorter gene lists are zero-padded. For each queried gene, the decoder generates an output embedding, which is passed through the expression head to produce the predicted expression value. Moreover, by integrating Cross-Flash Attention [46] into our attention layers, we drastically cut memory and compute requirements, allowing us to support maximum sequence lengths on the order of tens of thousands—sufficient to cover the full gene vocabulary. These mechanisms enable on-demand, dense prediction across the full genome (~ 60,000 genes) by simply adjusting the gene-ID (token) query list.

#### Chain-of-Continuous-Thought (Coconut) inference

We perform inference in multiple rounds using our Coconut [37] procedure (Additional file 1: Fig. S1–S2). Our Coconut procedure performs iterations without ever revealing individual gene tokens. At the start, only the CLS token is fed into the decoder, and all gene positions remain masked. On the first pass, the decoder produces an output embedding for each gene along with an uncertainty score. For the next iteration, those gene output embeddings are fed back as the new inputs. Instead of unmasking, we alter the attention mechanism so that at each round, gene positions attend preferentially to those with the highest confidence from the previous round. This feedback of embeddings together with uncertainty-guided attention adjustment is repeated for a fixed number of rounds. Each iteration sharpens the model’s estimates by letting confident predictions guide the remaining ones. After the final round, the CLS-driven output embeddings are projected to yield the genome-scale expression profile for that spot.

This procedure is a latent-space variant of chain-of-thought iteration: we first surface high-confidence gene predictions and then leverage them to refine others, analogous to how chain-of-thought (CoT) methods [47] iteratively build on intermediate conclusions. Crucially, we operate entirely on continuous output embeddings rather than re-tokenizing predicted values into discrete inputs, allowing the model to iterate beyond the confines of a fixed gene-expression token vocabulary [48]. Notably, the Coconut iterative inference mechanism in Coladan should not be understood as a large language model (LLM)-style chain-of-thought reasoning process.

#### Mixture of experts in gene decoder

To enhance the capacity of the gene decoder without sacrificing efficiency, we integrate a mixture-of-experts layer design based on DeepSeekMoE [36] into the transformer blocks (Additional file 1: Fig. S1). We apply two key design principles from DeepSeekMoE to maximize expert specialization. (1) Fine-grained expert segmentation: rather than large monolithic experts, we use a greater number of smaller experts by splitting the feed-forward network (FFN) intermediate neurons into finer segments. This means the total number of FFN parameters remains roughly constant, but divided among many experts. For example, instead of a single 4096-neuron hidden layer, an MoE layer might have 8 experts each with 512 neurons. At runtime, the gating network activates a small subset of these (e.g. 1 expert per token), combining their outputs. This fine segmentation allows each expert to learn more targeted, specific patterns in gene expression, and a more flexible combination of experts can be tailored to each gene’s context. (2) Shared expert isolation: we reserve a fraction of the experts (e.g. 1 out of 8) to be always active for every token as shared experts. These act as global experts that capture knowledge common to all genes (for instance, ubiquitous expression baselines or global normalization factors). By always including the shared experts, the model ensures that general features are accounted for, which in turn frees up the remaining conditional experts to focus on niche patterns without having to relearn the basics. The gated routing works as follows: for each gene token at each MoE layer, a softmax gating function (a small neural network taking the token’s current hidden state) produces a probability distribution over the E experts, and we select the top-k experts (with k = 1 in our setup) to compute that token’s FFN transform. The outputs of the selected experts (each weighted by the gate probability) are summed to form the layer output. During training, we apply standard MoE load-balancing losses to encourage an even usage of experts. The experts’ weights are initialized by partitioning the pretrained FFN layer’s weight matrices into separate expert modules and then applying small random perturbations to each, ensuring both a strong initialization and sufficient expert diversity. The MoE-augmented decoder thus learns to route different genes (and different contexts) through different expert subnetworks – a form of fine-grained modularization – which improves performance on this diverse prediction task. In summary, the DeepSeekMoE mechanism provides fine-grained expert specialization (many small experts per layer, each capturing a specific facet of gene regulation) and shared expert consolidation (a few experts concentrating common knowledge across genes), both of which help the decoder model the wide dynamic range of gene behaviors more effectively.

#### Perturbation prediction architecture and spatial perturbation prediction

First, to predict the effect of a perturbation on gene *X*, we inject into the gene decoder only the CLS embedding—either taken directly from a previous iteration or supplied by the slide encoder—alongside a single non-masked expression token for gene *X* set to its “0” (down) or “50” (up) (By specifying multiple genes simultaneously, the framework can simulate combinatorial perturbations.) and all other gene expression tokens masked. Under Coconut’s iterations, the decoder then generates genome-scale expression changes driven solely by that CLS context and the perturbed token. When that CLS embedding originates from an H&E encoder, this same procedure yields spatially resolved perturbation predictions, mapping how up- or down-regulation of gene *X* would alter gene expression at each tissue location or at selected subsets of locations. Crucially, it requires only the H&E-derived CLS embedding and the perturbed gene’s identifier—other in silico perturbation methods must supply full gene expression profiles.

#### Uncertainty quantification

(Additional file 1: Fig. S3) We model the predicted expression of each gene at spatial location **x** as

$$p(y \mid \mathbf{x}) = \mathcal{N}(y; \hat{\mu}(\mathbf{x}), \hat{\sigma}^{2}(\mathbf{x}))$$

where the network jointly outputs $\widehat{\mu }(\mathbf{x})$(mean) and $\widehat{\sigma }(\mathbf{x})$ (standard deviation) via a Gaussian negative log-likelihood (NLL) head.

##### Aleatoric uncertainty

The predicted $\widehat{\sigma }(\mathbf{x})$ captures measurement noise and intrinsic biological variation. Over T Coconut iterations we obtain ${\left\{{\widehat{\sigma }}_{t}\right\}}_{t=1}^{T}$. We estimate the aleatoric variance as.

$$\sigma_{\mathrm{alea}}^{2} = \hat{\sigma}_{T}^{2}.$$

 

##### Epistemic uncertainty

Each Coconut iteration re-injects the previous hidden state and re-selects a high-confidence gene subset, effectively sampling different regions of latent space with fixed parameters. From the predicted means ${\left\{{\widehat{\mu }}_{t}\right\}}_{t=1}^{T}$ we compute.

$${\sigma}_{\mathrm{e}\mathrm{p}\mathrm{i}}^{2}={\mathrm{V}\mathrm{a}\mathrm{r}}_{t}[{\widehat{\mu }}_{t}]\ ,$$

which quantifies model (epistemic) uncertainty without Monte Carlo (MC) dropout or ensembles [49].

##### Total predictive uncertainty

Assuming conditional independence,

$${\sigma}_{total}^{2}={\sigma}_{\mathrm{a}\mathrm{l}\mathrm{e}\mathrm{a}}^{2}+{\sigma}_{\mathrm{e}\mathrm{p}\mathrm{i}}^{2}$$

In practice, we compute these quantities on-the-fly during training. Across 32 held-out Visium slides, ${\sigma}_{total}$ shows a strong linear correlation with absolute prediction error (Pearson p = 6.6 × ${10}^{-3}$; Pearson p = 4.7 × ${10}^{-6}$), validating it as a reliable confidence measure.

#### Linear probe for per-gene on/off from CLS embeddings

For each Visium spot we extract the same image-conditioned CLS embedding that conditions the gene decoder in Coladan (the embedding produced after patch aggregation and slide-level contextualization). To explicitly model the presence/absence of expression before continuous regression, we attach a lightweight linear probe that maps this CLS embedding to one logit per gene; applying a sigmoid yield an estimated probability that the gene is expressed at that spot. Supervision is binary and derived directly from the count matrix: a gene is labeled “on” if its raw unique molecular identifier (UMI) count at that spot is non-zero after standard preprocessing, and “off” otherwise. The probe is trained on the Coladan-human3K training split with the same spot filtering used for the main model; we optimize a standard logistic classification objective with class-imbalance handling by per-class weighting (the positive class is rarer for most genes), early stopping on a held-out validation set, and weight decay for regularization. No image augmentations or additional context are used beyond the CLS embedding; the probe does not back-propagate into the encoders and does not alter the regression head, making it a true “probe” rather than a re-training of the backbone. At inference, the probe outputs per-gene probabilities for each spot; by default, we threshold at 0.5 to obtain on/off. All probe training and evaluation use the same gene lists and slide partitions as the main experiments. We report the effect of the probe as the change in the number of genes exceeding practical correlation thresholds (e.g., PCC or Spearman > 0.3).

#### TopRatePred: prediction-defined high-score evaluation for deployment

For real-world settings where ground truth is unavailable, we evaluate a prediction-guided region-of-interest (ROI) selection strategy termed TopRatePred. The procedure is as follows. First, we estimate, from the training set only, each gene’s expression on-rate as the fraction of spots with non-zero counts (i.e., the gene’s empirical prevalence of being expressed). This prevalence serves as the gene-specific baseline α that defines how many locations are expected to be informative for that gene on an average slide. Second, for a given keep rate we define a scaled target fraction by multiplying α by a preset factor (e.g., 0.05 ×, 0.10 ×, 0.20 ×, 0.30 ×, 0.40 ×, 0.50 ×, 0.90 ×, 1.00 ×, 1.20 ×, 1.50 ×) and clipping the result to at most all spots on a slide; this allows us to shrink or expand the retained proportion relative to a gene’s prevalence. Third, for each gene–slide pair we rank spots by Coladan’s predicted expression for that gene and retain only the top-ranked subset whose size matches the scaled target fraction for that gene on that slide (a small minimum number of spots is enforced to avoid degenerate sets). Crucially, only predictions are used to define ROIs—no measured values are consulted during selection—so TopRatePred avoids label leakage. Finally, correlations (Pearson and Spearman) are computed restricted to the retained spots and we summarize, per slide, the number of genes whose correlation exceeds a practical threshold (0.3 in our experiments). Additional file 1: Fig. S4a reports results across keep-rate settings, showing that high-confidence, top-ranked regions concentrate usable signal: as the retained fraction increases from very selective (e.g., 0.05 × α) toward almost all spots (e.g., 1.50 × α), the count of genes above the threshold decreases monotonically. TopRatePred is used purely as an evaluation-time strategy for deployment scenarios without ground truth; it does not influence training, hyperparameter selection, or model ranking elsewhere in the paper.

#### Multi-omics adaptation for protein expression

The Coladan architecture is inherently omics-agnostic, enabling straightforward extension to modalities beyond spatial transcriptomics. To illustrate this, we adapted Coladan to predict spatial protein expression from H&E images alongside mRNA. In this multi-omics adaptation, we freeze the pretrained patch encoder and slide encoder—preserving their ability to extract general histological features—and convert the original gene decoder into a protein decoder simply by replacing the gene-ID vocabulary with the corresponding protein-ID vocabulary. We then fine-tune this decoder using single-cell proteomics profiles—analogous to Coladan’s Stage 1 foundation adaptation—or spatial proteomics profiles—analogous to Coladan’s Stage 2 fine-tuning. Because many protein markers have well-known mRNA counterparts (for example, the *CD3E* gene and CD3 protein), the decoder can transfer its learned morphology–expression associations. After fine-tuning, gene decoder can generate spatial protein maps directly from routine histology, requiring no changes to the core architecture beyond the vocabulary swap. As shown in Additional file 1: Fig. S5a, this approach—leveraging the original transcript–vision pretraining—provides a preliminary proof-of-concept for zero-shot spatial proteomics inference after limited single-cell protein fine-tuning, highlighting the model’s potential to extend to other omics modalities.

### Training and ablation

#### Training stage 1

Adapting scGPT into a CLS-driven spatial gene decoder. We first adapted scGPT to spatial transcriptomics without histology image input. The goal of this stage was to establish a CLS-driven compression–decompression interface for spatial gene expression: measured spot/cell-level transcriptomic profiles are compressed into an expression-derived CLS representation, and queried gene-expression values are reconstructed from this compact representation. For each spot/cell, measured gene-expression values were encoded as expression embeddings and combined with gene-ID embeddings to form gene-token inputs. In the first step of the masking curriculum, 30–50% of expression embeddings were replaced by MASK tokens. The gene decoder first processed this partially masked gene-token sequence and produced an expression-derived CLS representation that summarized the measured transcriptomic profile, while reconstructing the masked gene-expression values. In the second step, all expression-state embeddings were replaced by MASK tokens, and the expression-derived CLS produced in the previous step was provided as the conditioning input to the decoder. The decoder was then trained to reconstruct the queried gene-expression values from this CLS information alone. This two-step curriculum teaches the gene model to encode high-dimensional spatial expression into a compact CLS representation and to decode this representation into gene-level outputs before any image features are introduced (Additional file 1: Fig. S6).

This Stage 1 forward pass used the modified scGPT decoder with MoE, Coconut and Gaussian negative log-likelihood regression head described above. The optimization objective combined Gaussian-NLL reconstruction with mixture-of-experts load-balancing regularization. Exact hyperparameter settings are listed in Additional file 1: Table S1. Notice that knowledge distillation [50] from the original model into the modified decoder rapidly restores performance before full Stage 1 training.

#### Training stage 2

Connecting the image pathway to the CLS-driven gene decoder. After Stage 1, the gene decoder had learned a CLS-conditioned expression space. We then connected the H&E image pathway to this decoder space in two phases.

In Phase 1, the patch encoder, slide encoder and gene decoder were frozen, and only the projector was optimized. For each target locus (64 × 64 px) a 224 × 224 px patch is extracted; the patch encoder produced a local embedding, which was processed together with patch coordinates by the slide-level encoder to incorporate whole-slide tissue context and generate a slide-aware locus embedding. A three-layer MLP projector then mapped this slide-aware embedding into the 512-dimensional CLS space expected by the gene decoder, producing an H&E-derived locus-CLS representation. In parallel, the matched measured spatial transcriptomic profile for the same target locus was passed through the Stage 1 adapted gene decoder to generate an expression-derived CLS representation. We optimized a CLS-projection loss that penalized the distance between the H&E-derived locus-CLS obtained from the projected slide-aware image embedding and the expression-derived CLS produced by the gene decoder from the measured expression profile of the same locus. This phase therefore trained the projector to translate slide-aware histology representations into the CLS space learned by the gene decoder, while keeping the image encoders and gene decoder fixed.

In Phase 2, the full network was unfrozen and trained end-to-end for image-to-expression prediction. For each target locus, the training forward pass followed the same image-conditioned pathway used at inference: the H&E patch and spatial coordinates were encoded into an H&E-derived locus-CLS, queried genes were specified by gene-ID embeddings, and gene expression embeddings were represented as MASK tokens. The gene decoder then predicted expression means and uncertainty estimates for the target locus. Measured spatial transcriptomic profiles were used as supervision targets for expression reconstruction and Gaussian-NLL regression, rather than as gene decoder inputs. The slide-to-language alignment was used to match slide embeddings to language embeddings obtained from curated sample information using the frozen MedGemma model. Together, these objectives optimized the complete H&E-to-expression pathway while preserving the trimodal slide-level representation learning.

#### Ablation

In our preliminary experiments, to dissect the contributions of our core architectural components and optimize key hyperparameters, we conducted complementary ablations (Additional file 1: Fig. S7).

##### Gene‐decoder variants

We compared five decoder configurations on the CLS-reconstruction task—quantifying mean squared error between the CLS-driven reconstructed expression profile and the true gene expression: Original: the off-the-shelf scGPT decoder with standard feed-forward sublayers (mean squared error (MSE) = 223). CoT (Chain-of-Thought) only: an uncertainty-guided chain-of-thought iteration in which each pass constructs its new input embedding by replacing the masked embeddings at high-confidence gene positions with their predicted expression values (MSE = 216). Coconut only: full Chain-of-Continuous-Thought iteration (latent feedback of output embeddings) without any mixture-of-experts routing (MSE = 206). MoE only: fine-grained + shared-expert isolation MoE routing applied to each feed-forward layer, with a single inference pass (MSE = 193). MoE + Coconut: our complete design, combining MoE routing with latent-space Coconut iterations, which achieved the best reconstruction error (MSE = 184) (Additional file 1: Fig. S7a).

##### Iteration count vs. runtime trade-off

We swept the number of Coconut inference iterations (C = 3, 5, 7, 11) and measured both CLS reconstruction MSE and per-epoch time. Error decreased from 210 at C = 3 to a minimum of 205 at C = 7, but the marginal gain from 5 → 7 iterations (206 → 205) did not justify the doubled runtime. We therefore adopted C = 5 for all experiments (Additional file 1: Fig. S7b).

##### Coconut training schedule

Rather than applying Coconut feedback from the outset, we staged our training across five phases. In Stage 0, the model is trained with standard CoT passes only. In Stage 1, a single latent-feedback “Thought” pass precedes each CoT pass; in Stage 2, two “Thought” passes are inserted, and so on up to Stage 4, which deploys four “Thought” passes per training step. This curriculum allows the model to gradually learn to leverage its own high-confidence embeddings before full Coconut training (Additional file 1: Fig. S7c and d).

##### Effect of Coconut inference depth

We also explored how the number of Coconut inference rounds (C) affects performance. Using our default C = 5 yields Pearson = 0.431 and Spearman = 0.295. When we varied C to 1, 3, 10, and 20, we obtained (Pearson,Spearman) pairs of (0.430,0.294), (0.433,0.305), (0.434,0.299), and (0.413,0.286), respectively. These results show that while a few extra iteration steps can provide marginal improvements, pushing C too high actually degrades correlation. In other words, “overthinking” does not necessarily yield better predictions and may even introduce noise into the inference [51]. (Additional file 1: Fig. S8e).

Together, these ablations validate that (1) mixture-of-experts routing and latent-feedback iteration each contribute substantial gains, (2) a moderate number of Coconut passes (five) balances accuracy and efficiency, and (3) a staged training schedule smooths the transition from standard CoT to full Coconut inference.

### Benchmark

We benchmarked Coladan against two complementary families of baselines. The first family comprised seven leading pathology foundation models—MUSK [52], UNI2 [22], CONCH v1.5 [21], TITAN [26], GigaPath [23], Virchow [25], and H-Optimus-0 [53]—each employing its own architecture and preprocessing pipeline. For instance, some baselines use larger input tiles (384 × 384 px) instead of our 224 × 224 px patches, while others vary in transformer depth or hidden dimension. The fine-tuning protocols and hyperparameter settings for these baselines are detailed in Additional file 1: Table S2-S8. The second family comprised three directly related image-to-expression methods—DeepSpot, BLEEP, and HisToGene—representing pathology-pretrained regression, joint embedding/retrieval, and patch-and-coordinate transformer prediction, respectively. The fine-tuning protocols and hyperparameter settings for these baselines are detailed in Additional file 1: Note 1. All baselines were fine-tuned on our Coladan-human3K data following their original training recipes to ensure a fair comparison. For all baselines, we selected the 2,500 genes most frequently expressed across the dataset as their fixed prediction panel for training and testing. In contrast, Coladan’s decoder allows arbitrary gene queries and is configured with a maximum input length of 5,000 per spot, thereby accommodating nearly all expressed genes in each sample (a more demanding setting). To ensure a fair comparison unbiased by zero-expression values, we observed that each spot got on average ~ 1,300 genes on fixed panel and, following our gene-expression preprocessing, only ~ 100 genes per spot remained zero—amounting to roughly 7% of the gene count (after gene-expression preprocessing filter). To ensure a fair comparison, we therefore randomly retained only 7% of the zero-valued genes per spot (i.e. matching the zero-to-nonzero ratio observed post-preprocessing) when computing Pearson and Spearman correlations for Coladan. Overall, during training Coladan is fed the full preprocessed gene list for each spot—namely, every expressed gene plus the randomly sampled zero‐expression genes (up to 35% of the nonzero count). At evaluation time, we adopt the same scheme: for each spot we supply Coladan only the set of expressed genes and a matched 7% subset of zero‐count genes (the exact zero-to-nonzero ratio observed in 2,500 fixed gene panel), and the model then predicts expression values for precisely those gene tokens.

#### Gene-level benchmark

All methods were compared under the same 15,000-gene benchmark universe. For each gene, Pearson and Spearman correlations were computed only across loci with non-zero ground-truth expression for that gene (non-zero-loci evaluation), thereby reducing dilution from extreme zero inflation in slide-wide gene-level statistics. We then counted, for every method, the number of genes whose Pearson or Spearman correlation exceeded 0.3, using the same cutoff throughout. All methods were evaluated on aligned sample-wise prediction matrices from the same held-out Visium cohort under the same matrix-level correlation code. As these baselines do not optimize identical objectives, spot-wise and gene-wise rankings need not coincide; brief method-specific notes and interpretation of this divergence are provided in Additional file 1: Note S1.

#### Benchmark for perturbation

For fairness, all compared methods were evaluated on the same condition-level perturbation benchmark and the same train/test split protocol. scGPT was initialized from the published pretrained checkpoint and fine-tuned following the official perturbation pipeline; GEARS was trained following its original recommended pipeline on the same training split; and the linear baseline was fit on the same training data. For Perturb-seq benchmarking, matched histology was not available. Therefore, the baseline-state CLS representation used by the perturbation module was derived from the corresponding unperturbed/control expression profile rather than from image features. Specifically, the control expression vector for each condition was passed through the gene decoder, and the resulting CLS embedding was extracted to represent the pre-perturbation cellular state for downstream perturbation-response prediction. By contrast, in the spatial image-only deployment setting, the CLS representation was obtained directly from histology, and no measured expression input was provided at inference. This distinction separates the benchmark setting, which tests perturbation-response modeling on a shared baseline-state representation, from the spatial application setting, which tests image-conditioned perturbation extrapolation.

Held-out test perturbation conditions were not exposed during training. Performance was quantified using Pearson_Δ, defined as the Pearson correlation between predicted and observed Δ-expression for each perturbation condition. We evaluated two CLS-embedding-only perturbation variants of Coladan. In the multi-task setting, perturbation-response learning was coupled to the image-to-expression framework during training. In the zero-shot setting, no perturbation supervision was used during training, and only the perturbation label was provided at inference. The zero-shot variant also showed a weak but detectable effect, despite receiving no perturbation supervision during training.

#### Benchmark for VisiumHD and spot-level Xenium

VisiumHD and Xenium were evaluated as platform-level zero-shot test sets that were set aside before model development. For VisiumHD, zero-shot correlations were computed directly on the selected native VisiumHD output layer, rather than after post hoc downsampling to conventional Visium resolution. Unless otherwise stated, the main zero-shot VisiumHD results were obtained on the square_016 µm layer, which was chosen because it best matched the effective spot scale of the processed training datasets after preprocessing. We additionally repeated the analysis on the finer square_008 µm layer to assess cross-scale robustness. Thus, the reported VisiumHD correlations reflect direct evaluation on the target output layer itself, not retrospective aggregation to standard Visium resolution. For Xenium, zero-shot evaluation was performed directly on the HEST-style [33] processed Visium-like spot-based units defined after preprocessing, rather than on native cell polygons. Correlations were first computed at the spot level on these processed evaluation units and then summarized within each slide as per-slide averages of spot-wise correlations. No post hoc downsampling to conventional Visium resolution was applied. Accordingly, for both VisiumHD and Xenium, zero-shot performance was assessed directly on the predefined processed evaluation units of the target dataset, rather than by first predicting at one resolution and then retrospectively downsampling to match conventional Visium. For both models, the zero-shot evaluations used the same processed Bin50 target matrices and the same 64 × 64 px prediction target region; only the surrounding image input window followed each model's native backbone requirement.

For representative spot-level Xenium spatial visualization, we selected four sample-gene pairs from the same held-out Xenium slides used for the spot-level Xenium evaluation. These examples were chosen to cover representative sample contexts, biologically interpretable genes, sufficient measured signal, and visually interpretable regional patterns, rather than to define an additional quantitative benchmark. The selected examples included *POU2F3* in primary dermal melanoma, *CTSG* in prostate adenocarcinoma, *PDCD1* in fibrotic IPF lung, and *TNFRSF17* in IPF lung (Additional file 1: Fig. S9). For each example, we visualized the H&E image, native Xenium transcript locations overlaid on H&E, the processed Xenium-derived measured spot-level Bin50 expression, and the corresponding Coladan-predicted spot-level Bin50 expression. The native transcript overlays were used as molecule-level spatial references, whereas the processed spot-level measured maps were the benchmark targets used for quantitative evaluation. For visualization only, measured and predicted maps were independently normalized to 0–1 after clipping at the 90th percentile of their respective displayed non-zero values; all reported correlations were computed from Bin50 values.

### Evaluation

#### Gene-wise and spot-wise

We report two complementary correlation statistics.

Spot-wise correlation evaluates, for each spot s, how accurately the model recovers its entire expression profile. Let ${{\boldsymbol{y}}}_{s}\in {\mathbb{R}}^{G}$ be the ground-truth log-expression vector of G genes, and ${\widehat{{\boldsymbol{y}}}}_{s}$ the prediction. The Pearson (or Spearman) coefficient is

$${r}_{\mathrm{s}\mathrm{p}\mathrm{o}\mathrm{t}}\left(s\right)=\frac{{\sum}_{g=1}^{G}\left({y}_{sg}-\overline{{y }_{s}}\right)\left(\widehat{{y}_{sg}}-{\overline{\widehat{y}}}_{s}\right)}{\sqrt{{\sum}_{g=1}^{G}{\left({y}_{sg}-\overline{{y }_{s}}\right)}^{2}}\hspace{0.17em}\sqrt{{\sum}_{g=1}^{G}{\left(\widehat{{y}_{sg}}-{\overline{\widehat{y}}}_{s}\right)}^{2}}}$$

where ${\overline{y} }_{s} = \frac{1}{G}{\sum}_{g}{y}_{sg}$ and ${\overline{\widehat{y}} }_{s} = \frac{1}{G}{\sum}_{g}{\widehat{y}}_{sg}$. We compute ${r}_{spot}\left(s\right)$ for every spot and report its mean over all spots in a slide.

Gene-wise correlation evaluates, for each gene g, how well the spatial pattern of that gene is reproduced. Denote the vector of ground-truth expression across S spots as ${y}_{g}\in {\mathbb{R}}^{S}$ and the predictions as ${\widehat{{\boldsymbol{y}}}}_{g}$. The coefficient is

$${r}_{\mathrm{g}\mathrm{e}\mathrm{n}\mathrm{e}}\left(g\right)=\frac{{\sum}_{s=1}^{S}\left({y}_{sg}-\overline{{y }_{g}}\right)\left(\widehat{{y}_{sg}}-{\overline{\widehat{y}}}_{g}\right)}{\sqrt{{\sum}_{s=1}^{S}{\left({y}_{sg}-\overline{{y }_{g}}\right)}^{2}}\hspace{0.17em}\sqrt{{\sum}_{s=1}^{S}{\left(\widehat{{y}_{sg}}-{\overline{\widehat{y}}}_{g}\right)}^{2}}}$$

With ${\overline{y} }_{g} = \frac{1}{S}{\sum}_{s}{y}_{sg}$ and ${\overline{\widehat{y}} }_{g} = \frac{1}{S}{\sum}_{s}{\widehat{y}}_{sg}$. We average ${r}_{gene}\left(g\right)$ over all genes.

Throughout the main text, “sample-level correlation” refers to the mean gene-wise Pearson (or Spearman) coefficient over all genes in a slide computed with this protocol.

#### Mitigating zero‐inflation bias in correlation metrics

Sparse zero counts can artificially inflate both Pearson and Spearman correlations—patches with mostly zeros will trivially agree on non‐expression, yielding deceptively high scores. To avoid this zero‐inflation bias in our Coladan benchmarks, we down‐sampled each spot’s zero‐count genes to match the typical zero:non-zero ratio (~ 0.07) we found in competing methods. This correction ensures that reported correlations reflect true agreement.

#### Cluster separation and ASW

We assessed how well the model’s latent space and predictions preserved biological cluster structure using the average silhouette width (ASW) metric. We took the slide embeddings from Coladan and TITAN, then performed dimensionality reduction using uniform manifold approximation and projection (UMAP; Additional file 1: Fig. S7g) [54] and clustering. For known biological categories such as tissue type and disease type, we computed the silhouette width for each point with respect to those categories. A good integration would show high silhouette for true biological classes and low silhouette for modality labels (meaning points mix by modality). ASW is the mean silhouette across points (ranging from −1 to 1, where higher means better cluster separation for the chosen label). In our evaluations (Additional file 1: Fig. S7e), we observed an improved ASW for tissue and disease type in the joint latent space of our model, indicating that our model retains foundation-model capacity even without purely self-supervised training, and that adding the spatial-gene stream further strengthens slide-level representations.

#### Pathway-level directional reproducibility and discordance analysis

We evaluated pathway-level recovery across six fixed sample-pair comparisons, including four primary biological contrasts—Normal_vs_Cancer_Prostate, Parent_vs_Targeted_SpinalCord, Parent_vs_Targeted_Glioblastoma, and Parent_vs_Targeted_ColorectalCancer—and two low-signal section-level controls—Sect. 1_vs_Section2_Breast_BlockA_40_39 and Sect. 1_vs_Section2_Breast_BlockA_46_45. We used five curated pathway collections: NCI-Nature_2016, MSigDB_Oncogenic_Signatures (C6), MSigDB_Hallmark_2020 [55], KEGG_2021_Human, and Reactome_Pathways_2024.

For each comparison, genes were ranked separately from the original expression profiles and from model-predicted profiles. Pathway enrichment was computed using preranked gene set enrichment analysis (GSEA) [56] implemented in gseapy [57] (1,000 permutations; minimum gene-set size = 10; maximum gene-set size = 500; identical settings across methods and pathway collections). To ensure a fair method comparison, Coladan and CONCH v1.5 were re-evaluated under a matched-gene-universe setting, such that both methods were assessed using the same genes for each pair and each panel size.

We defined anchor-pathway coverage as the fraction of original high-priority pathways retained under a given panel, and same-direction recovery as the fraction of covered anchor pathways whose enrichment direction matched between the original and predicted analyses. To assess signal dependence, we further stratified pathways by the strength of the original signal using the absolute normalized enrichment score (NES) from the original data, denoted absolute NES in the original data (abs NES_ori), and computed same-direction recovery within each stratum. Representative discordant anchor pathways were then inspected to identify patterns associated with weak-signal pathways and broad or overlapping pathway ontologies (more details in Additional file 1: Note S2). The same ranking-and-enrichment pipeline was applied to both original and predicted profiles for all methods, and all downstream pathway summaries were derived from the resulting matched-gene-universe comparisons.

#### Coarse compartment overlays for perturbation map interpretation

To interpret the displayed prostate perturbation maps against local tissue structure, we generated coarse compartment overlays from marker-guided expression scores. For normal prostate sections, spots were assigned to epithelial, stroma, immune or others. Epithelial scores were computed from *EPCAM*, *KRT8*, *KRT18*, *KRT19*, *KRT17*, *KRT5*, *KRT14* and *TACSTD2*; stromal scores from *COL1A1*, *COL1A2*, *DCN*, *LUM*, *ACTA2*, *TAGLN*, *VIM*, *PDGFRB* and *FBLN1*; and immune scores from *PTPRC*, *CD3D*, *CD3E*, *MS4A1*, *LYZ*, *NKG7*, *TYROBP*, *LST1* and *HLA-DRA*. For cancer sections, spots were assigned to tumor-enriched, stroma, immune or others. Tumor-enriched scores were computed from *EPCAM*, *KRT8*, *KRT18*, *KRT19*, *TACSTD2*, *KLK3*, *KLK2*, *ACPP*, *FOLH1* and *AMACR*, whereas stromal and immune scores used the same marker panels as above.

Marker scores were computed from the baseline expression matrix, and local k-nearest-neighbour smoothing was applied to reduce isolated label noise. These compartment labels were used only for visualization and interpretation of the displayed perturbation maps and were not used as supervisory labels during model training.

#### Spatial perturbation summary metrics

For each perturbation condition, spatial perturbation maps were defined as Δ = (perturbed prediction − baseline prediction) at the spot level. For the displayed perturbation examples, the primary quantitative spatial summaries were the mean signed Δ values within each coarse compartment. These region-level mean deltas were used to assess whether a displayed example showed a predominantly positive response, a predominantly negative response or mixed-direction behavior across epithelial/tumor-enriched, stromal, immune and other tissue contexts.

For downstream control analyses, we additionally computed several perturbation-structure metrics from Δ across spots. Mean_abs_delta was defined as the mean of |Δ| across all spots and was used to summarize overall response magnitude. Contrast_gap_abs was defined as the difference between mean |Δ| inside the strongest change compartment and mean |Δ| outside that compartment, and was used to quantify compartment-level contrast. Std_delta was defined as the standard deviation of signed Δ across spots. Gini_absdelta was computed from |Δ| to quantify how unevenly perturbation magnitude was distributed across the tissue, with larger values indicating that the response was concentrated in a smaller subset of spots rather than spread broadly across the slide. MoranI_abs_delta was computed from |Δ| to quantify spatial autocorrelation of perturbation magnitude, with larger values indicating that spots with similar perturbation magnitude tended to cluster spatially rather than being randomly dispersed.

#### Gini coefficient

To quantify the inequality of the same absolute change values across spots, we computed the Gini coefficient using the standard Lorenz-curve formulation on non-negative data. The metric equals zero when absolute changes are perfectly even across spots and increases toward one as the changes become concentrated in a subset of spots, reflecting stronger spatial localization of the perturbation effect.

#### Housekeeping-like negative controls

To assess whether the displayed spatial structures were stronger than expected for arbitrary stable genes, we constructed negative controls from a predefined panel of canonical housekeeping and broadly expressed reference genes present in the dataset. The panel comprised *ACTB*, *GAPDH*, *TUBB*, *TUBA1B*, *RPLP0*, *RPL13A*, *RPL11*, *RPL19*, *RPS18*, *RPS3*, *RPS8*, *EEF1A1*, *UBC*, *HSP90AB1*, *HSP90AA1*, *PPIA*, *HPRT1*, *B2M*, *PGK1*, *GUSB*, *SDHA*, *YWHAZ*, *TFRC*, *TOMM20*, *SNRPD3*, *PSMB2*, *CYC1*, *ATP5F1B*, *RAN* and *HNRNPA1*. For each displayed case and each perturbation summary metric, we computed the same metric for the target gene and for all housekeeping genes under the same perturbation condition.

The observed value for the displayed target gene was then compared against the distribution of housekeeping values from the same case. Empirical P values were calculated as the fraction of housekeeping genes whose metric value was greater than or equal to the observed target value. This analysis was used to test whether the displayed examples showed stronger perturbation structure than would be expected for genes with relatively stable baseline behavior.

#### Matched empirical null and exact-entry audit

To control for gene-specific detectability and prediction difficulty, we constructed a matched empirical null within each perturbation condition. For each displayed target gene, all other genes in the same perturbation condition were ranked by similarity in three features: nonzero-locus counts in the original expression matrix, baseline predicted mean expression and baseline predicted variance. These three quantities were standardized, and Euclidean distance in the resulting three-dimensional feature space was used to rank candidate background genes. The nearest matched pool was then formed from the top 250 ranked background genes.

For each perturbation-structure metric defined above, null distributions were generated by Monte Carlo resampling with replacement from this matched pool, using 5,000 draws per target gene and metric. In the corresponding supplementary table, observed denotes the metric value of the displayed target gene, null_mean denotes the mean of the matched null distribution, obs_minus_null denotes the difference between the observed value and the matched null mean, and p_empirical denotes the empirical P value, calculated as the fraction of Monte Carlo null draws greater than or equal to the observed value. Because these null distributions were generated by a finite number of Monte Carlo draws, the smallest attainable empirical P value was determined by the number of resamples.

To assess whether the displayed prostate-specific driver–target pairs were directly represented in the perturbation training resource, we additionally performed an exact-entry audit over perturbation identity, perturbation direction and target gene. Displayed cases not found as exact-matched entries were interpreted as out-of-resource perturbation examples rather than direct replays of seen perturbation conditions.

#### MoE routing diagnostics

To characterize the internal organization of the mixture-of-experts (MoE) decoder, we analyzed routing statistics for every decoder layer and Coconut iteration in Coladan-human3K. Because all MoE analyses in this study used top-1 routed selection, each valid gene token contributed exactly one routed-expert assignment. For each sample, we pooled all valid gene tokens within a given decoder layer and Coconut iteration, and then computed the fraction of tokens assigned to each routed expert. From this expert-usage distribution, we derived two complementary summary statistics. First, we recorded the maximum expert usage, defined as the largest token fraction assigned to any single expert, which quantifies how strongly routing concentrates on one dominant expert. Second, we computed the effective number of experts using the inverse Simpson index, which summarizes how many experts are meaningfully engaged after accounting for load imbalance. Under this definition, values closer to 1 indicate near-collapse onto a single expert, whereas larger values indicate more distributed routing across experts. We also quantified routing uncertainty before top-1 selection by computing the Shannon entropy of the full gate softmax distribution over routed experts for each valid token, and then averaging this quantity across tokens within each sample, layer, and iteration. Higher mean routing entropy indicates a flatter, less decisive routing distribution, whereas lower entropy indicates sharper expert preference. In addition, because each MoE block contains both routed experts and an always-active shared branch, we quantified the shared ratio from the relative magnitudes of the shared and routed branch outputs. Specifically, for each valid token we measured the L2 norm of the shared-branch output and the L2 norm of the routed-branch output, and defined the shared ratio as the shared-branch magnitude divided by the sum of the shared- and routed-branch magnitudes; this quantity was then averaged over valid tokens. Higher shared ratio therefore indicates that the shared pathway contributes more strongly relative to the routed pathway. Unless otherwise noted, these routing statistics were first computed at the sample level for each layer and Coconut iteration and then averaged across samples. For tissue-stratified analyses, the same quantities were recomputed after regrouping tokens by tissue rather than by individual sample. Because the final Coconut iteration is most directly linked to the final gene-expression output, iteration 3 was used for downstream expert-role and pathway-level analyses.

#### Matched-null pathway-to-routing co-assignment analysis

To further assess whether the learned MoE routing structure showed measurable correspondence with existing curated biological pathway annotations, we performed a matched-null pathway-to-routing co-assignment analysis using the final refinement iteration. The MoE decoder contains routed experts and an always-active shared branch. In the routed branch, each valid gene token contributes one selected routed-expert assignment at each MoE layer under top-1 routed selection, whereas the shared branch remains active for all valid tokens. The analysis below focused on selected routed-expert assignments from the routed branch.

For each gene and decoder layer, selected routed-expert assignments were aggregated across the evaluation set. Specifically, for each gene within a given layer, we counted its assignments to each routed expert and normalized these counts to obtain a gene-level selected-expert profile. We then evaluated curated Hallmark, KEGG, and Reactome pathways. For each pathway, observed pathway coherence was computed as the mean pairwise cosine similarity among the selected-expert profiles of member genes. Higher coherence indicates that genes within the same pathway show more similar selected routed-expert assignment patterns.

To estimate the matched null distribution, we generated 500 random gene sets for each pathway by sampling from the same routed-gene universe, with each random set containing the same number of genes as the corresponding pathway. For each random set, we computed the same pathway coherence statistic. We reported coherence delta as the difference between observed pathway coherence and the mean coherence of matched random pathway sets. We also computed a null-standardized coherence score:

$${Z}_{\mathrm{c}\mathrm{o}\mathrm{h}}=\frac{{C}_{\mathrm{o}\mathrm{b}\mathrm{s}}-{\mu}_{\mathrm{n}\mathrm{u}\mathrm{l}\mathrm{l}}}{{\sigma}_{\mathrm{n}\mathrm{u}\mathrm{l}\mathrm{l}}}$$

where ${C}_{\mathrm{o}\mathrm{b}\mathrm{s}}$ is the observed pathway coherence, and ${\mu}_{\mathrm{n}\mathrm{u}\mathrm{l}\mathrm{l}}$ and ${\sigma}_{\mathrm{n}\mathrm{u}\mathrm{l}\mathrm{l}}$ are the mean and standard deviation of coherence values from matched random gene sets. One-sided empirical P values were computed as:

$${P}_{\mathrm{e}\mathrm{m}\mathrm{p}}=\frac{1+\sum_{i=1}^{{N}_{\mathrm{r}\mathrm{a}\mathrm{n}\mathrm{d}\mathrm{o}\mathrm{m}}}I({C}_{\mathrm{n}\mathrm{u}\mathrm{l}\mathrm{l},i}\ge {C}_{\mathrm{o}\mathrm{b}\mathrm{s}})}{1+{N}_{\mathrm{r}\mathrm{a}\mathrm{n}\mathrm{d}\mathrm{o}\mathrm{m}}}$$

where ${C}_{\mathrm{n}\mathrm{u}\mathrm{l}\mathrm{l}}$ denotes coherence values from matched random gene sets and ${N}_{\mathrm{r}\mathrm{a}\mathrm{n}\mathrm{d}\mathrm{o}\mathrm{m}}=500$. To summarize layer-wise evidence, we reported, for each decoder layer and pathway library, the number of pathways tested, the mean and median coherence delta, the mean and median null-standardized coherence score, and the fraction of pathways with one-sided empirical *P<0.05*. These empirical *P* values were used as descriptive matched-null statistics to assess pathway-level routing coherence, rather than to claim definitive pathway-level significance for individual gene sets.(Additional file 1: Table S9).

#### Virtual gene expression mapping on TCGA slides

To demonstrate Coladan’s potential for in silico spatial‐omics on routine histology, we applied the model to 50 TCGA H&E whole‐slide images. For each slide, we followed the exact pipeline outlined in the “TCGA histology dataset for virtual profiling” section. We then assessed how predictive uncertainty scales with the size of the gene panel by randomly drawing, without replacement, subsets of 1,000, 2,000, 4,000, and 6,000 genes from the 20,000 most highly expressed human genes. For each gene panel, Coladan generated 1,000, 2,000, 4,000, 6,000 genes expression predictions at every grid point, and computed uncertainty (Methods “Uncertainty quantification”). Finally, we summarized the median uncertainty across all spots for each panel size, illustrating that while uncertainty increases with panel size, it remains relatively low across all settings, and highlighting a blueprint for future synthetic-data-driven model scaling.

#### Computing hardware and software

All experiments and analyses were implemented in Python 3.10 and PyTorch 2.0.1 with CUDA 11.7 (https://pytorch.org) [58]. Coladan was trained using PyTorch's Distributed Data Parallel (DDP) across 4 NVIDIA A100 80 GB GPUs, totaling 4,000 + GPU hours. All other computations for downstream experiments and benchmark were conducted on a single 24 GB NVIDIA GeForce RTX 3090 GPU (On a single 24 GB NVIDIA GeForce RTX 3090 GPU, end-to-end inference or image-only perturbation for a typical Visium Sect. (3–5 k spots) completes in ~ 10 min). Implementations of the pathology foundation models we benchmarked are publicly available at the following links: GigaPath (github.com/prov-gigapath/prov-gigapath), UNI2 (https://huggingface.co/MahmoodLab/UNI2-h), Virchow (https://huggingface.co/paige-ai/Virchow), TITAN (https://huggingface.co/MahmoodLab/TITAN), MUSK (https://huggingface.co/xiangjx/musk), H-Optimus-0 (https://huggingface.co/bioptimus/H-Optimus-0), CONCH v1.5 (https://huggingface.co/MahmoodLab/conchv1_5). Exact hyperparameters for fine-tuning these models are detailed in Additional file 1: Table S2-S8. Implementations, training settings, and benchmark notes for the specialized image-to-expression baselines, including DeepSpot, BLEEP, and HisToGene are summarized in Additional file 1: Note S1. And they are publicly available at the following links: DeepSpot (https://github.com/ratschlab/DeepSpot), BLEEP (https://github.com/bowang-lab/BLEEP), HisToGene (https://github.com/maxpmx/HisToGene).

## Results

### Coladan dataset, training workflow and model architecture overview

**Fig. 1:**
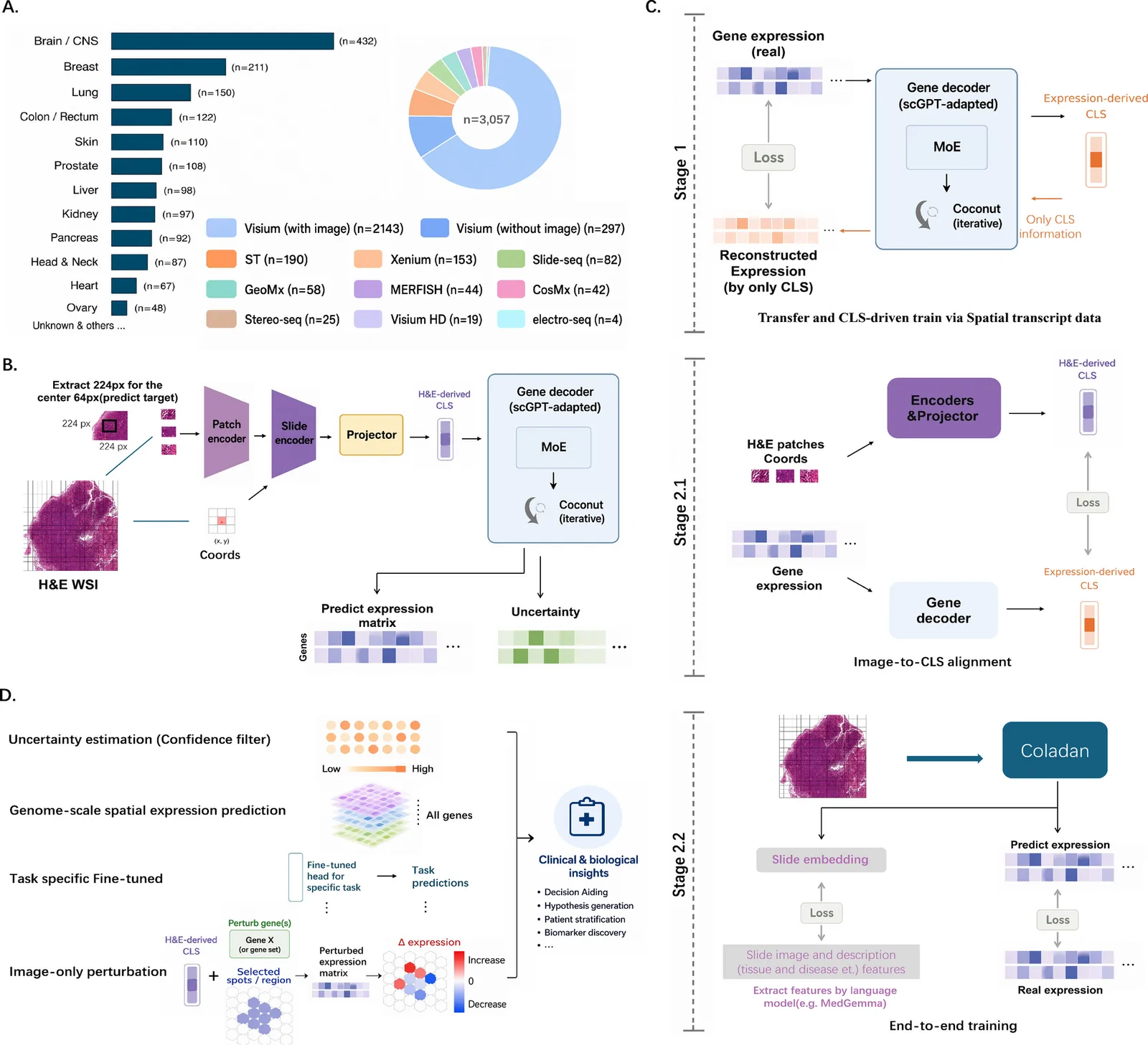
Overview of Coladan. **a** Composition of the Coladan-human3K resource by tissue/organ category and spatial omics platform. **b** Coladan architecture for H&E-to-expression prediction. For each target locus, a 224 × 224 px H&E patch centered on the 64 × 64 px prediction region is extracted from the whole-slide image. The patch encoder generates local image features, which are combined with spatial coordinates and contextualized by the slide encoder. A projector maps the resulting slide-aware representation into an H&E-derived CLS embedding, which is then passed to a scGPT-adapted gene decoder with MoE routing and Coconut iterative decoding to predict spatial gene expression and uncertainty. **c** Training workflow. Stage 1 adapts the gene decoder to reconstruct measured spatial transcriptomic profiles from expression-derived CLS representations. Stage 2.1 aligns H&E-derived CLS embeddings with expression-derived CLS embeddings. Stage 2.2 trains the full image-to-expression model end-to-end, with additional slide-level alignment to language-derived sample descriptions. **d** Downstream applications enabled by Coladan, including confidence filtering, genome-scale spatial expression prediction, task-specific fine-tuning, and image-only perturbation to support clinical and biological insights

Coladan is a whole-slide, transformer-based framework [59, 60] for genome-scale dense prediction of spatial gene expression from H&E histopathology images. By integrating histology images, sample-level language information and spatial-gene representations, Coladan learns morphology-informed molecular features within whole-slide tissue context. To support robust training and evaluation, we assembled Coladan-human3K, the largest publicly available human-only spatial transcriptomics resource to date. Coladan-human3K comprises ~ 3,000 spatial profiles—spanning diverse tissues and disease states—collected from multiple public repositories and platforms (Fig. 1a; Additional file 2). This represents a threefold increase in overall cohort size and nearly a fourfold increase in exclusively human samples compared to existing resources (e.g., HEST-1 k [33], STimage-1K4M [34]), providing unparalleled statistical power and biological diversity for model development and benchmarking.

Coladan consists of four core modules—a patch encoder, a slide-level encoder, a language module, and a CLS-driven iterative gene decoder—and leverages Chain-of-Continuous-Thought iteration alongside fine-grained DeepSeekMoE routing. During inference (Fig. 1b), for each target locus, a 224 × 224 px patch is cropped for context and expression is predicted for its central 64 × 64 px area. The patch encoder produces an embedding that, concatenated with slide coordinates, is processed by the slide encoder to inject global tissue context and generate slide-aware locus embeddings. A lightweight projector maps this feature to the H&E-derived locus-CLS representation, which the gene decoder converts into expression values for that locus. Total predictive uncertainty is computed as the sum of aleatoric variance from the Gaussian negative log-likelihood (NLL) head of the gene decoder and epistemic variance estimated across Coconut inference passes (see Methods "Uncertainty quantification").

First training stage (overview in Fig. 1c). We begin with a large self-supervised single-cell foundation model (scGPT [35]) and adapt it to spatial data through a CLS-driven decoding strategy: a single CLS embedding can iteratively generate expression profiles for any subset of genes—up to the full genome (~ 60,000 genes)—per spot/cell. In this stage, no histology image input was used; measured spatial transcriptomic profiles were used to teach the decoder to compress spot/cell-level expression states into an expression-derived CLS embedding and to reconstruct queried gene-expression values from this compact representation (Additional file 1: Fig. S6; see Methods “Training stage 1”). To support this CLS-driven spatial gene decoding setting, we introduce three key changes. First, we integrated DeepSeek mixture-of-experts routing with fine-grained micro-experts and a small shared pool for granular specialization and parameter-efficient activation (Additional file 1: Fig. S1). Second, we incorporated Chain-of-Continuous-Thought (Coconut) iteration, which reuses hidden-state embeddings across passes to perform structured multi-step latent computation in continuous embedding space (Additional file 1: Fig. S1). Third, we apply an uncertainty-guided iterative CLS decoding scheme that combines Gaussian-NLL–derived uncertainty with Coconut inference passes to prioritize high-confidence genes and guide subsequent rounds (workflow in Additional file 1: Fig. S2; Methods). Ablations show each component reduces reconstruction loss (Additional file 1: Fig. S7; see Methods “Ablation”).

Building on pre-trained vision and language backbones (GigaPath patch/slide encoders [23] and language module of MedGemma [45]), we extend Coladan from single-spot (patch) to dense, whole-slide inference. The second training stage consists of two phases [61]. Phase 1 optimizes only the projector to map slide-aware locus embeddings into the decoder CLS space. The resulting H&E-derived locus-CLS embeddings were aligned to expression-derived CLS embeddings generated by the Stage 1 decoder from the matched measured spatial transcriptomic profiles. Phase 2 unfreezes the entire network and introduces two additional objectives: (i) a reconstruction loss between predicted and measured gene expression for each locus, and (ii) an alignment loss that matches slide embeddings to language embeddings obtained by encoding our curated sample information with the frozen MedGemma model [45]. Removing the language stream yields negligible change in gene-level accuracy (Additional file 1: Fig. S7f). Slide embeddings from our trimodal model (image + language + spatial genes) remain foundation-model–level and are slightly more discriminative: average silhouette width (ASW) = 0.23 vs 0.21 for TITAN (Additional file 1: Fig. S7e). This indicates that adding the spatial-gene stream does not erode the pretrained representation and even strengthens slide-level semantics, so the model continues to support slide-level foundation-model tasks after fine-tuning.

After training, Coladan supports multiple downstream applications through the framework (Fig. 1d), including dense genome-scale spatial expression prediction, predictive uncertainty estimation for confidence-aware filtering, gene- and region-selective image-only in silico perturbation, and task-specific fine-tuning using learned whole-slide and locus-level representations. The CLS-conditioned decoding framework supports image-only perturbation, which estimates perturbation-associated expression changes at selected tissue locations without requiring measured spatial transcriptomics as inference input.

### Benchmark on 30 + standard 10 × Genomics spatial transcriptomics datasets vastly exceed existing methods

Coladan achieves state-of-the-art accuracy on 32 unseen Visium datasets spanning nine organs and benign–malignant states, including multiple tumor types with matched normal tissues (Additional file 1: Fig. S8a), none of which were used for training. We benchmarked Coladan against two complementary families of methods. The first comprised seven strong pathology-foundation baselines (UNI-2 [22], Virchow [25], TITAN [26], MUSK [52], H-Optimus-0 [53], GigaPath [23] and CONCH v1.5 [21]) under matched protocols. The second comprised three specialized image-to-expression baselines—DeepSpot [30], BLEEP [28] and HisToGene [18]—representing pathology-pretrained regression, joint-embedding/retrieval, and patch-and-coordinate transformer prediction, respectively. Coladan flexibly predicts a genome-scale vocabulary (see Methods "Flexible genome-scale prediction"), rather than a fixed panel. To ensure a fair comparison we down-sampled zero-valued genes so that the ratio of zero to non-zero entries matched that used for the baselines fixed panel. A panel-size ablation of CONCH v1.5 showed accuracy decreasing as the panel grew, with a steep early drop that plateaued beyond ~ 3 k genes (Additional file 1: Fig. S8c).

**Fig. 2:**
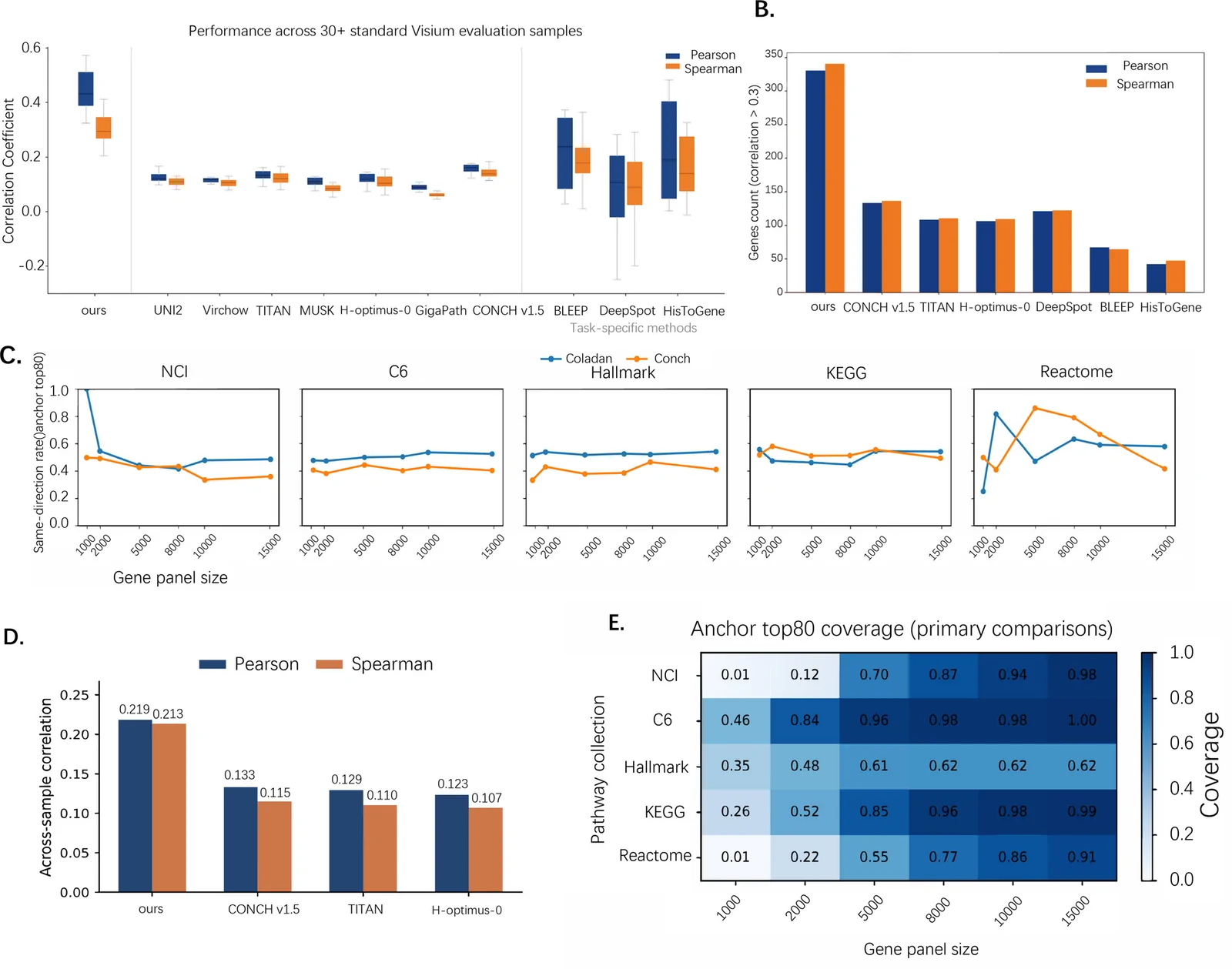
Benchmark performance. **a** Mean spot-wise Pearson (blue) and Spearman (orange) correlations on 32 unseen 10 × Genomics Visium slides for seven fine-tuned baselines and Coladan (ours). Bars show the average across slides (ours (Coladan): 0.431/0.295; UNI-2: 0.131/0.110; Virchow: 0.121/0.103; TITAN: 0.140/0.126; MUSK: 0.124/0.090; H-Optimus-0: 0.137/0.119; GigaPath: 0.097/0.059; CONCH v1.5: 0.168/0.143; BLEEP: 0.213/0.181; DeepSpot: 0.078/0.065; HisToGene: 0.230/0.169). Boxes show the interquartile range (IQR; 25th–75th percentiles); center line is the median; whiskers indicate the minimum and maximum across slides. **b** Number of genes exceeding a practical accuracy threshold. Counts of genes with Pearson (blue) or Spearman (orange) > 0.3 under the non-zero-loci evaluation protocol (correlations computed only at Visium spots where the gene is non-zero). Coladan (ours) yields the largest pool of usable genes, substantially exceeding all fine-tuned baselines run under the same genome-scale training setup. **c** Same-direction recovery across gene panel sizes under matched gene universes. For each pathway collection (National Cancer Institute [NCI], Molecular Signatures Database oncogenic signature gene-set collection [C6], Hallmark, Kyoto Encyclopedia of Genes and Genomes [KEGG], and Reactome), curves show the mean same-direction rate among top 80 anchor pathways across the primary biological comparisons, comparing Coladan (blue) and CONCH v1.5 (orange) at 1 k, 2 k, 5 k, 8 k, 10 k and 15 k genes. Coladan is generally more competitive at larger panels, indicating that higher gene-wise fidelity supports more consistent pathway signals. **d** Sample-level agreement across slides. Mean sample-level (slide-level) Pearson (blue) and Spearman (orange) correlations—computed by aggregating per-slide correlations across all genes—on the benchmark Visium cohort. Coladan achieves the highest agreement across slides, while TITAN and H-Optimus-0 track CONCH v1.5 at a lower level. **e** Anchor-pathway coverage across pathway collections and gene panel sizes. Heatmap showing the mean coverage of anchor top80 pathways across the primary biological comparisons for each pathway collection and gene panel size. Values denote the proportion of original anchor pathways retained under the corresponding gene panel, illustrating the expected increase in pathway coverage as panel size expands

Coladan outperformed every competing model by a large margin (Fig. 2a; see Additional file 1: Table S10-S11 for per-slide benchmark details). Across the 32 slides the mean Pearson correlation (see Methods "Gene-wise and spot-wise") reached 0.431, 1.9-fold higher than the best baseline (0.230, HisToGene), while Spearman correlation (spot-wise) reached 0.295, 1.6-fold higher than the best baseline (0.181, BLEEP). Notably, language-augmented models consistently outperformed purely vision-based backbones: the vision transformer (ViT)-based GigaPath lagged even after extensive fine-tuning, supporting the view that “language-augmented” visual models capture richer histomorphologic cues [60, 62].

On Visium, slide-wide gene-level Pearson correlation coefficient (PCC) and Spearman computed across all spots are near zero because extreme zero inflation dilutes signal from expressing regions (consistent with prior reports [63]). We therefore use a non-zero-loci evaluation (for each gene, correlations are computed only at loci that are non-zero), under which both metrics increase and stabilize, and Coladan yields far more genes with PCC or Spearman > 0.3 than baselines (Fig. 2b). This is expected: conditioning on expression turns the task from rare-event detection into recovering rank and relative magnitude, improving rank statistics and mitigating PCC dilution. It also aligns with vision-to-transcriptome mapping that leverages single-cell-derived cell-level priors to better discriminate genuinely expressing spots even when absolute values are imperfect. For fairness, the same non-zero-loci evaluation and the same 0.3 practical cutoff were applied to all methods in this benchmark; full protocol details are provided in the Methods section. Interestingly, the gene-wise ranking is not identical to the spot-wise ranking: DeepSpot becomes more competitive on this axis and overtakes BLEEP and HisToGene, although it still remains below CONCH v1.5. This likely reflects differences in what these models are optimized to recover (Additional file 1: Note. S1).

To model zeros explicitly, we add a lightweight logistic on/off probe to CLS embeddings (see Methods "Linear probe for per-gene on/off from CLS embeddings"). While global means change little, this substantially increases the number of genes exceeding practical thresholds (e.g., PCC or Spearman > 0.3; Additional file 1: Fig. S4b), indicating that improved binary detection moves borderline genes into a usable regime; larger gains in mean correlation likely require coupling such detectors with stronger continuous regression. Finally, for real-world deployment without ground truth, we adopt a prediction-guided region-of-interest (ROI) selection strategy (see Methods "TopRatePred: prediction-defined high-score evaluation for deployment"): for each gene and slide, spots are ranked by Coladan’s predicted expression and only the top-ranked subset is retained at a pre-specified keep rate, under which the count of genes above 0.3 remains consistently favorable (Additional file 1: Fig. S4a), showing that high-confidence regions contain actionable signal without label leakage. Together, these results support a practical recipe for gene-level use: (i) pair continuous predictors with a lightweight on/off probe. (ii) compute readouts and statistics only on the model’s top ranked loci to concentrate reliable signal and reduce false positives and sparsity-driven noise.

We assessed pathway recovery via directional concordance between pred-vs-pred and ori-vs-ori enrichments (see Methods "Pathway-level directional reproducibility and discordance analysis"). Using six fixed sample-pair comparisons spanning four primary biological contrasts (including normal-versus-cancer prostate and parent-versus-targeted comparisons in spinal cord, glioblastoma, and colorectal cancer) together with two low-signal section-level controls, and evaluating five curated pathway collections (NCI, C6, Hallmark, KEGG, and Reactome), we found that Coladan showed pathway-level enrichment consistency beyond single-gene accuracy and performed better in more comparison–ontology settings (Fig. 2c). The strongest gains were observed among anchor pathways and pathways with stronger original signals (Additional file 1: Fig. S10a). The remaining discordant cases were enriched in weak-signal pathways (Additional file 1: Fig. S10b, c) and in broader, more overlapping pathway collections, suggesting that pathway-level instability is concentrated in specific signal regimes rather than uniformly distributed across all biological programs. Detailed cross-ontology comparisons and signal-stratified analyses are provided in (Additional file 1: Note. S2) and (Additional file 3).

At the sample level (PCC and Spearman computed per slide across all genes and then averaged across slides), Coladan attains mean PCC = 0.2186 and Spearman = 0.2134 across the benchmark cohort, substantially outperforming CONCH v1.5 (0.1328 and 0.1148, respectively); TITAN and H-Optimus-0 track CONCH v1.5 and remain below our method on both metrics (Fig. 2d). This confirms that gains observed locally (spot- and gene-wise) persist when summarized at the slide level.

Efficient compression–decompression in a compact latent space. Despite relying on a mere 512-dimensional hidden vector per spot, Coladan achieves the highest accuracy (Additional file 1: Fig. S8b), demonstrating that our CLS + Coconut + MoE–based compression–decompression pipeline effectively preserves gene-expression structure and spot-level information.

Capturing gene-specific variation. To verify that Coladan learns gene-specific rather than “pan-cellular” signals from the foundation model, we counted the number of unique predicted values per gene across the test set (Additional file 1: Fig. S11). Over 95% of genes assume more than 400 distinct values, confirming that the decoder models high-dynamic-range variability instead of collapsing all cells into a single latent embedding.

### CLS-embedding-only perturbation and image-only in silico perturbation

**Fig. 3:**
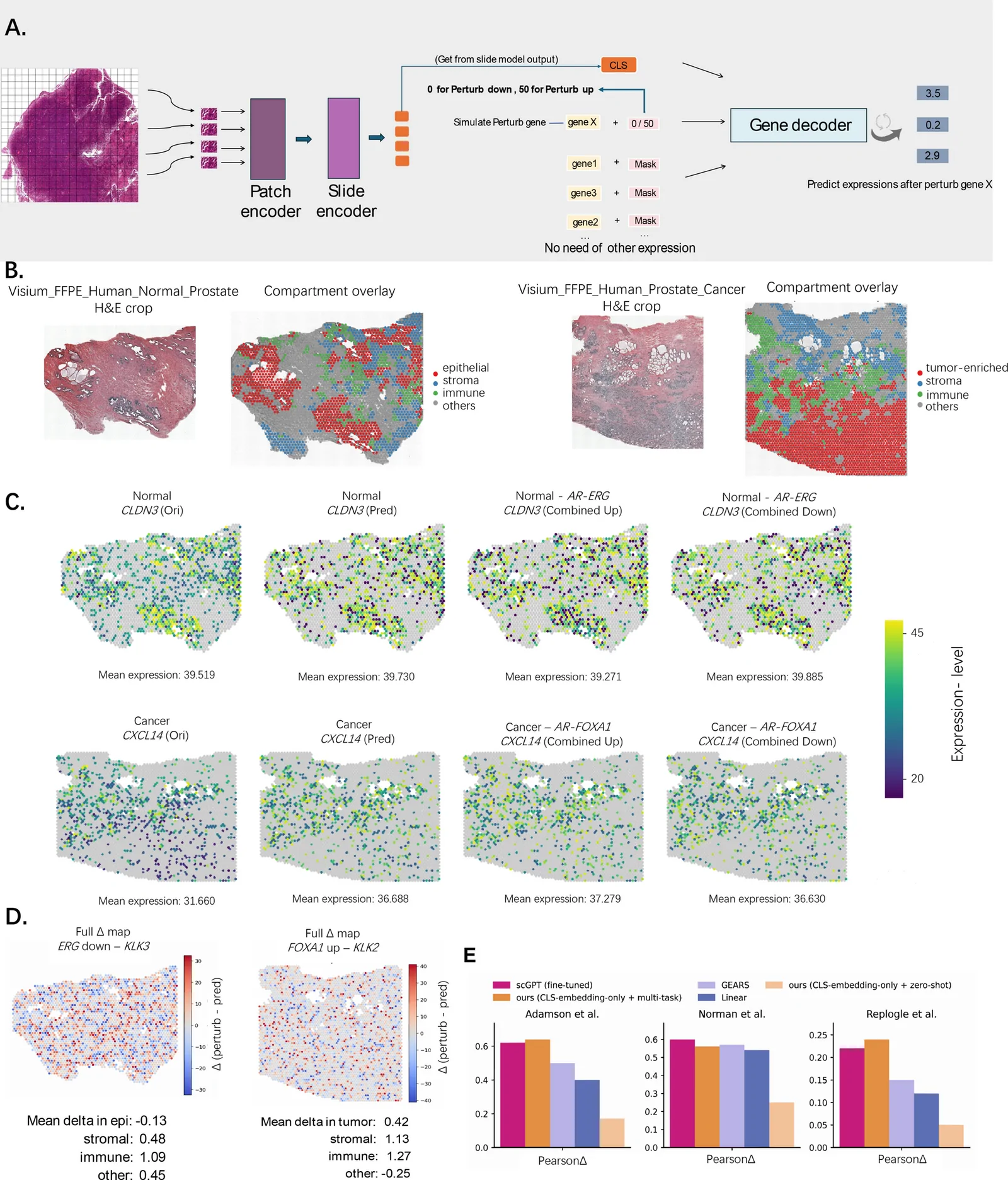
CLS-embedding-only perturb prediction and spatial image-only perturbation. **a** Workflow for in silico spatial perturbation: H&E patches → patch encoder → slide encoder → CLS token. To simulate up/down regulation of gene X, the expression token is set to “0” or “50” for that gene and passed—without any measured expression—to the gene decoder under Coconut iteration generation. **b** Histological context for displayed perturbation examples. Representative H&E crops and coarse compartment overlays are shown for one normal prostate Visium section and one prostate cancer Visium section. Normal prostate spots were assigned to epithelial, stroma, immune and others compartments, whereas cancer spots were assigned to tumor-enriched, stroma, immune and others compartments using marker-guided coarse scoring (see Methods "Coarse compartment overlays for perturbation map interpretation"). **c** Representative spatial perturbation (image-only) on Visium prostate slides. Normal prostate (top) and prostate cancer (bottom) slides. Columns are, from left to right: Ori (measured), Pred (unperturbed), and CLS-driven expression after simulated up-/down-regulation (Combined-Up/Combined-Down) for the specified axis (*AR*–*ERG*; *AR*–*FOXA1*). Colors denote per-spot expression level. The direction of change for the genes (*CLDN3* and *CXCL14*) is concordant with prior studies. These examples illustrate that Coladan produces spatially coherent image-only perturbation effects without using measured expression. **d** Full spatial perturbation maps for single-perturbation examples. Left, Normal | *ERG* down—> *KLK3*. Right, Cancer | *FOXA1* up—> *KLK2*. Color indicates the signed perturbation response, defined as Δ = (perturbed prediction − baseline prediction), with red indicating higher predicted expression under perturbation and blue indicating lower predicted expression relative to baseline. These maps complement the compartment-based summaries reported in Additional file 1: Table S12. **e** Quantitative comparison on perturbation benchmarks (Pearson correlation of expression changes, Pearson_Δ). Across three datasets—Adamson (n = 87), Norman (n = 283), and Replogle (n = 4,450)—our CLS-embedding-only + multi-task variant performs on par with fine-tuned scGPT, and surpasses GEARS and a linear baseline. The zero-shot variant (trained without perturbation data) still yields reproducible non-zero Pearson_Δ

We next asked whether perturbation responses could be inferred from histology alone. Perturbation supervision was available only from single-cell Perturb-seq resources rather than from spatial perturbation data, so the spatial maps examined below should be interpreted not as direct spatial ground-truth reconstructions but as image-conditioned extrapolations of perturbation responses. In our inference setting (Fig. 3a), each spot was represented only by its slide-level CLS embedding together with perturbation condition, and perturbed expression was generated by the gene decoder under Coconut iterations without measured expression inputs at inference time. Unlike perturbation frameworks that require control expression vectors or a prespecified gene order at inference (e.g., scGPT [35]; GEARS [64]; Geneformer [65]), this CLS-only formulation turns perturbation prediction into a histology-context-conditioned counterfactual interface, enabling comparisons at the same spatial locus while avoiding direct leakage of measured expression into spatial inference.

We evaluated two CLS-only perturbation variants: a multi-task variant trained with perturbation supervision and a zero-shot variant that never saw perturbation data during training and received only the perturbation label at inference. For perturbation benchmarking, the baseline-state CLS representation was derived from matched control expression rather than from histology, whereas in the spatial application below the CLS representation was obtained directly from image features (see Methods "Perturbation prediction architecture and spatial perturbation prediction"). Across the Adamson, Replogle and Norman Perturb-seq [38–40] benchmarks, the multi-task variant performed on par with fine-tuned scGPT and consistently outperformed GEARS and a linear baseline, measured by Pearson_Δ (see Methods "Benchmark for perturbation"; Fig. 3e). The zero-shot variant still produced reproducible non-zero Pearson_Δ across all datasets, suggesting that the model retained non-trivial perturbation-relevant signal even without explicit perturbation supervision in this setting. A possible explanation is that this behavior is supported by the iterative completion dynamics used during baseline gene-expression training; we discuss this interpretation further in Supplementary Note 3. Taken together, these results support the feasibility of direct image-only in silico perturbation within an intact whole-slide context and, to our knowledge, provide the first such demonstration.

To examine biological plausibility in situ, we perturbed several key prostate drivers, including *AR*, *PTEN*, *FOXA1* and *ERG* [66], on normal and cancer prostate 10 × Genomics Visium sections (Fig. 3b). Illustrative combinatorial perturbation maps are shown for *AR*–*FOXA1* and *AR*–*ERG* (Fig. 3c; Additional file 1: Fig. S12a-c). These predicted responses were organized along biologically interpretable prostate axes, with *CXCL14* highlighting a stromal/microenvironment-associated field in *AR*–*FOXA1* [67] and *CLDN3* highlighting an epithelial/tight-junction-associated field in *AR*–*ERG*, broadly consistent with *ERG*-linked rewiring of prostate differentiation programs [68, 69] (Fig. 3c). These combinatorial examples are therefore presented as pathology-anchored, image-conditioned perturbation readouts rather than direct spatial validation.

Single-perturbation maps further resolved region-biased response fields in prostate tissue (Fig. 3d; Additional file 1: Table S12). In normal tissue, *ERG* down—> *KLK3* showed the clearest directionally concordant pattern [69] (Fig. 3d), with predominantly positive responses across stromal, immune-rich and mixed regions and only a small negative epithelial shift. In cancer tissue, *FOXA1* up—> *KLK2* provided the most concordant luminal example [70] (Fig. 3d), with positive mean signed Δ across tumor-enriched, stromal and immune-rich compartments. By contrast, *AR* down—> *KLK2* in normal tissue showed stronger regional polarization with mixed directional behavior, whereas *ERG* down—> *CLDN3* in cancer tissue was predominantly negative despite clear regional structure (Additional file 1: Fig. S12e; details in Note. S3). Quantitative spatial summaries based on per-compartment mean signed Δ confirmed that these examples span both directionally concordant and anatomically structured response fields rather than spatially uniform causal maps (see Methods "Spatial perturbation summary metrics"; Additional file 1: Table S12).

Finally, we asked whether the displayed spatial structures were stronger than expected for arbitrary stable genes or arbitrary matched background genes. The displayed cases remained more separated from housekeeping controls across multiple perturbation metrics, and several also deviated from matched empirical-null expectations after controlling for nonzero-locus counts, baseline predicted mean expression and baseline predicted variance (see Methods "Housekeeping-like negative controls" and “Matched empirical null and exact-entry audit”; Additional file 1: Fig. S12d; Additional file 1: Table S13). In addition, displayed driver–target pairs did not appear as exact-matched entries in the perturbation training resource. Together, these analyses support interpreting the displayed maps as image-conditioned, out-of-resource, hypothesis-generating spatial extrapolations rather than simple replays of seen training examples.

### Robust zero-shot generalization to VisiumHD and spot-level Xenium

#### Zero-shot benchmarking across VisiumHD and spot-level Xenium

**Fig. 4:**
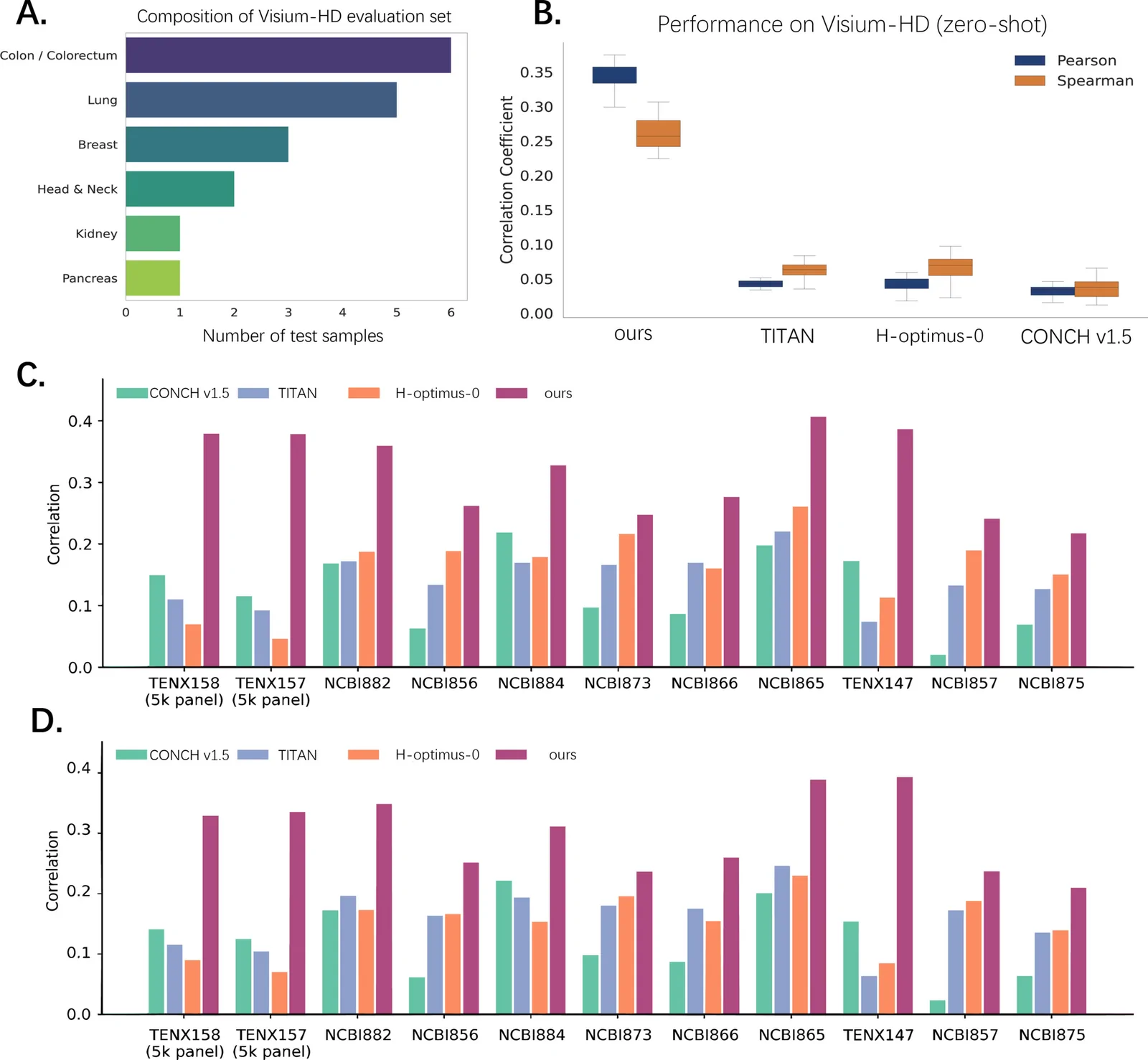
Zero-shot generalization to VisiumHD and spot-level Xenium. **a** Composition of the VisiumHD evaluation set by tissue type (total n = 18 slides): colon/colorectum (6), lung (5), breast (3), head and neck (2), kidney (1), pancreas (1). **b** Zero-shot performance on VisiumHD (square_016 µm output layer): spot-wise Pearson (blue) and Spearman (orange) correlations for Coladan and top baselines, presented as mean Pearson/Spearman: ours (Coladan): 0.338/0.25; TITAN: 0.042/0.062; H-Optimus-0: 0.040/0.060; CONCH v1.5: 0.03/0.03. Boxes show the IQR (25th–75th percentiles); center line is the median; whiskers indicate the minimum and maximum across slides. **c** Zero-shot performance on 10 × Genomics spot-level Xenium (spot-wise Pearson by slides). Per-slide correlations (average of spot-wise correlations) across 11 Xenium slides: TENX158 (5 k panel) and TENX157 (5 k panel) and NCBI882, NCBI856, NCBI884, NCBI873, NCBI866, NCBI865, TENX147, NCBI857, NCBI875. Coladan (ours) consistently attains the highest Pearson correlation without any fine-tuning or domain adaptation, outperforming CONCH v1.5, TITAN and H-Optimus-0 on every slide. **d** Zero-shot performance on 10 × Genomics spot-level Xenium (spot-wise Spearman by slides). Same slides and ordering as in **c**; Coladan again ranks first on all slides

We evaluated Coladan without fine-tuning on 18 publicly released VisiumHD slides spanning colorectum, lung, breast, head & neck, kidney, and pancreas (Fig. 4a). Among the available output grids, we selected the square_016 µm layer (median spot diameter ~ 64 px in full-resolution H&E), which best matches the effective spot size in our processed training datasets, and treated its measured expression as ground truth. Under identical zero-shot settings, Coladan retained meaningful predictive power on VisiumHD (mean spot-wise Pearson = 0.338), whereas baseline models collapsed toward near-zero correlations across the evaluation (Fig. 4b; Additional file 1: Table S14-S15 for per-slide details). We further assessed cross-platform transfer on 11 Xenium-derived spot-level slides spanning the 5k targeted panel (TENX158, TENX157) and additional predesigned small targeted panels (Fig. 4c–d; Additional file 1: Table S16-S17 for per-slide details). Coladan consistently achieved the highest per-slide spot-wise Pearson/Spearman; in contrast, baselines performed poorly on the 5k panel and only reached acceptable scores on smaller panels.

#### Resolution robustness

Repeating the VisiumHD evaluation at the finer square_008 µm layer, Coladan maintained strong performance (Pearson = 0.269; Spearman = 0.206), whereas baselines approached zero (best baseline ≈0.028/0.027), confirming that the advantage persists across spatial scales and molecule-depth regimes (Additional file 1: Fig. S8d).

### Uncertainty-guided reliability filtering

Reliable translational use of image-to-omics models requires uncertainty estimates that track prediction error and support confidence-aware filtering. Coladan addresses this by predicting, for each gene at each spatial spot, both a mean and a data-dependent standard deviation through a Gaussian negative log-likelihood (NLL) objective, yielding an estimate of aleatoric uncertainty. This NLL term also stabilizes Coconut training (Additional file 1: Fig. S2), without which iterative decoding diverges after only a few epochs. To capture epistemic uncertainty, we further exploit Coconut’s multi-step iterations: repeated inference passes re-inject the previous hidden state while re-masking different high-confidence subsets, and the variance across passes serves as a proxy for epistemic uncertainty (Additional file 1: Fig. S3a). These components are combined into a spot-level predictive uncertainty score (see Methods, “Uncertainty quantification”). Consistent with this role, the Coconut depth sweep showed that increasing iterative decoding depth produced only limited changes in point-prediction accuracy (Additional file 1: Fig. S8e), suggesting that Coconut contributes more clearly to repeated latent decoding for epistemic uncertainty estimation and reliability-aware filtering. We further compared three uncertainty formulations—a cross-entropy (CE)-head entropy baseline, a point-wise Gaussian model, and an interval-aware Gaussian model—to assess how uncertainty quality and predictive fidelity trade off in downstream use (Additional file 1: Note. S4).

**Fig. 5:**
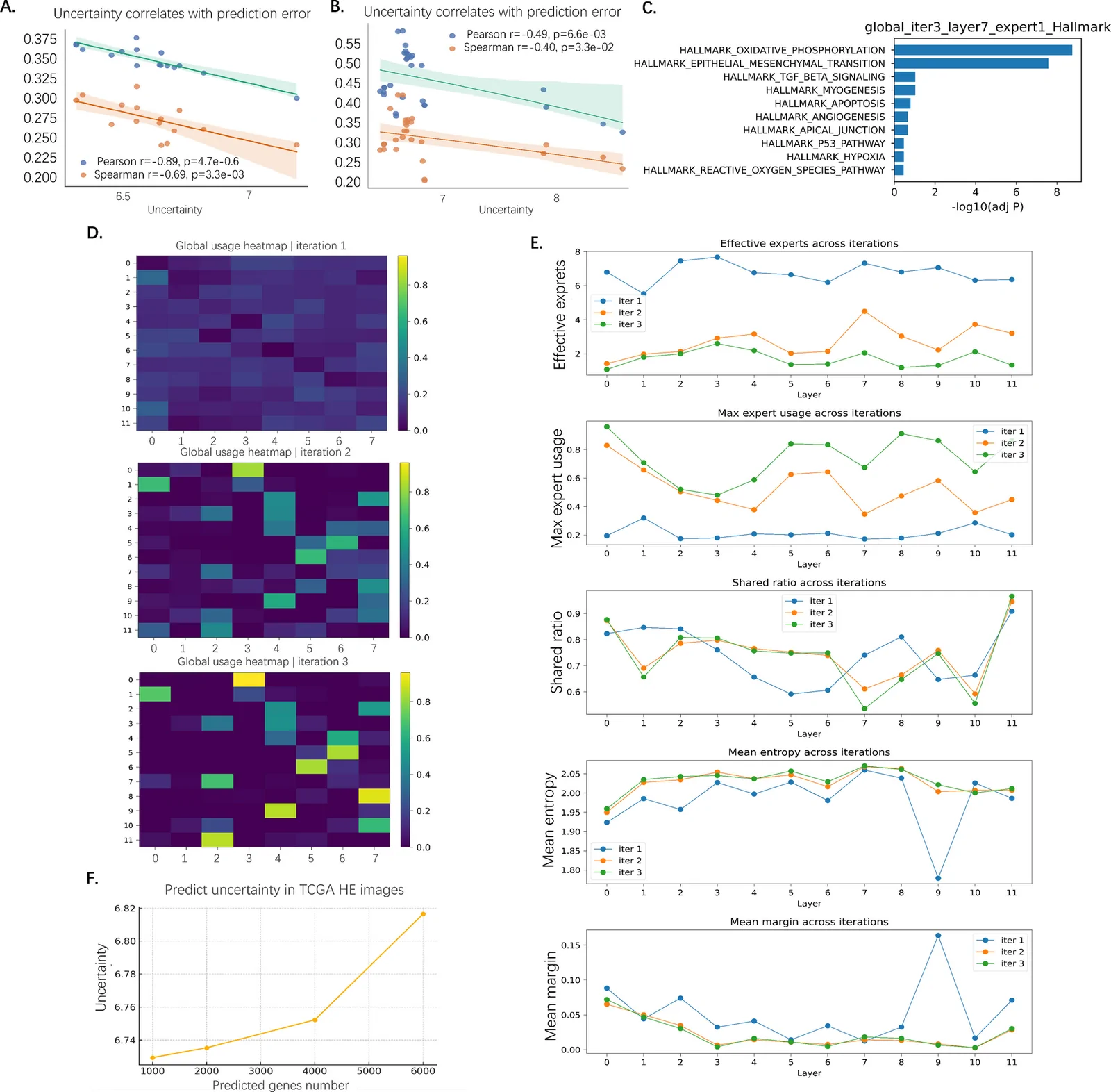
Uncertainty-guided filtering, internal organization of MoE, and virtual deployment. **a** Model-predicted uncertainty (aleatoric + epistemic variance) versus absolute Pearson correlation on the same VisiumHD test slides. Each point represents one slide; accuracy is the mean spot-wise Pearson r. The negative trend (Pearson r = −0.89, p = 4.7 × 10⁻⁶; Spearman r = −0.69, p = 3.3 × 10⁻^2^) confirms that higher estimated uncertainty reliably flags lower prediction fidelity. The shaded bands denote 95% confidence intervals around each regression. **b** Model-predicted uncertainty (aleatoric + epistemic variance) versus absolute Pearson correlation on the same Visium test slides. Each point represents one slide; accuracy is the mean spot-wise Pearson r. The negative trend (Pearson r = −0.49, p = 6.6 × 10⁻^3^; Spearman r = −0.4, p = 3.3 × 10⁻^2^) confirms that higher estimated uncertainty reliably flags lower prediction fidelity. The shaded bands denote 95% confidence intervals around each regression. **c** Pathway enrichment for a representative routed expert. Shown are the top Hallmark pathways enriched among genes most frequently routed to layer 7, expert 1 at the final Coconut iteration step (iteration 3). This expert is prominently associated with oxidative phosphorylation/respiratory electron transport-related programs, illustrating that selected routed components can be biologically annotated. **d** Global routed-expert usage heatmaps across the three Coconut iterations. Each row denotes a decoder layer and each column denotes a routed expert; color indicates the fraction of valid gene tokens assigned to that expert within the corresponding layer. **e** Layer-level routing summaries across Coconut iterations. From top to bottom, panels show the effective expert number, maximum expert usage, shared ratio, mean routing entropy, and mean top1–top2 routing margin for each decoder layer at iterations 1, 2, and 3. Together, these statistics show that MoE routing is layer-dependent rather than uniform, with a subset of layers retaining clearer routed-expert diversity and others showing more concentrated, shared-dominated behavior by the final iteration step. **f** Virtual genome-scale inference on TCGA whole-slide H&E images. Median per-spot predictive uncertainty (aleatoric + epistemic) is plotted as the requested gene panel expands from 1,000 to 6,000 genes, demonstrating only a gradual increase in uncertainty and suggesting feasibility of synthetic-data-driven training strategies

Across our benchmarks, the result showed the model-predicted uncertainty correlates strongly with absolute prediction error (Pearson p = 6.6e-03; Fig. 5b). The same linear relationship persists in zero-shot VisiumHD inference (Pearson p = 4.7e-06; Fig. 5a), indicating that uncertainty estimates transfer across resolutions. To test whether uncertainty is directly actionable, we ranked spots by predicted uncertainty and recalculated average spot-wise correlations after retaining only the lowest-uncertainty 25%, 50%, 75%, or all spots (Additional file 1: Fig. S13b). In both Gaussian formulations, lower-uncertainty spots consistently corresponded to higher-fidelity regions, with nearly identical retention behavior. Calibration analysis further showed that the interval-aware Gaussian achieved higher empirical coverage at the cost of wider intervals, whereas the point-wise Gaussian produced sharper predictions (Additional file 1: Fig. S13d, e). Thus, predictive uncertainty provides a practical signal for prioritizing spatial regions for interpretation, visualization, candidate-region selection, and downstream whole-slide deployment.

### Internal organization and biological annotatability of the MoE architecture

To characterize the internal organization of the MoE decoder, we quantified expert routing across all decoder layers and across the three Coconut iterations (Fig. 5e). Because the final Coconut iteration is most directly linked to the final gene-expression output, we used iteration 3 for downstream expert-role analyses. Routing was not uniformly distributed throughout the decoder; instead, layers 3, 4, 7, and 10 retained clearer expert diversity, whereas other layers showed more consolidated, shared-dominated behavior. These dominant routing patterns were also broadly conserved across tissues at the dominant-pattern level rather than being strongly reconfigured in a tissue-specific manner (Additional file 1: Fig. S14).

Gene- and pathway-level analyses showed that selected routed components were associated with recognizable biological annotations. Representative expert-associated gene sets were enriched for oxidative phosphorylation and respiratory electron transport (Fig. 5c), proteostasis and antigen-processing programs, membrane trafficking and RAB geranylgeranylation, and signaling-related regulation including NOTCH- and MYC-associated pathways (Additional file 1: Fig. S15a, c, d). We further performed a pathway-centric matched-null coherence analysis across decoder layers to assess whether known pathway membership was reflected in the broader selected-expert profile structure. This analysis showed that pathway-level coherence was not uniformly distributed across the decoder, but was positive in a subset of layers relative to random gene sets (Additional file 1: Fig. S16). Together, these results indicate that MoE specialization in Coladan involves biologically annotatable selected routed components and partial concordance between the learned routing structure and existing curated pathways. Detailed analyses are provided in Additional file 1: Note S5.

### Extension to other spatial omics and virtual clinical deployment

Coladan’s modular design also permits preliminary extension beyond transcriptomic decoding. As a proof-of-concept, we adapted the decoder to a small spatial proteomics setting [41, 42]; these exploratory results are presented in Additional file 1: Note. S6.

Leveraging the uncertainty delivered by our dual-variance estimator (aleatoric + epistemic; Methods “Uncertainty quantification”), we next applied Coladan in silico to TCGA whole-slide H&E images [71], generating virtual genome-scale spatial expression profiles across diverse tumor types. As the requested gene panel expanded from 1,000 to 6,000 genes, the median per-spot uncertainty increased only gradually (Fig. 5f), reflecting a controlled confidence degradation. These virtual predictions on TCGA slides provide a blueprint for future uncertainty-guided synthetic-data-driven training strategies [72].

## Discussion

Leveraging the Coladan-human3K resource—the largest human-only spatial-transcriptomics compendium to date (about 3,000 samples)—we introduce Coladan, a transformer-based, calibrated whole-slide image-to-omics model that predicts genome-scale (about 60 k genes) expression directly from H&E images. Key architectural innovations include a fine-grained mixture-of-experts router [36], Chain-of-Continuous-Thought (Coconut) [37] iterative decoding, and a CLS token that aggregates global context. A Gaussian negative-log-likelihood head learns aleatoric variance, while Coconut iterations expose an epistemic component, yielding calibrated per-gene uncertainty. A training recipe with bin-based supervision and controlled zero-sampling stabilizes zero-inflated targets; in practice, a compact 512-D latent suffices to preserve expression structure. Finally, the framework is modular, allowing task-specific decoders to be swapped while reusing the image backbone and conditioning, although in the current study the proteomics extension should be regarded as a preliminary proof-of-concept rather than a main result. Together these elements enable state-of-the-art accuracy and, importantly, image-only in silico perturbation within an intact whole-slide context.

Coladan further provides three scaled advantages: genome-scale accuracy, cross-platform generalization, and uncertainty calibration. First, Coladan delivers practical genome-scale utility: improving spot-wise correlations translates into more usable genes under non-zero-loci evaluation and more directionally consistent pathway, with gains that persist after slide-level aggregation. Second, Coladan generalizes in true zero-shot fashion across platforms and resolutions—including VisiumHD and Xenium—without any domain adaptation, supporting robust deployment when chemistry, resolution, or preprocessing shift. Third, Coladan provides calibrated uncertainty by decomposing predictive variance into aleatoric and epistemic terms; confidence-based filtering or prioritization enriches for more reliable spatial predictions, helping to close a practical reliability gap for image-based molecular inference. Collectively, these properties make Coladan not only more accurate, but also more usable and trustworthy at scale.

We further show that perturbation responses can be predicted from histology alone. Inference proceeds from CLS embeddings plus perturbation labels, with no measured expression or pre-specified gene order. On single-cell Perturb-seq benchmarks, a multi-task perturbation setting shows competitive performance against strong sequence-based baselines, while a zero-shot setting still recovers reproducible response directionality. In spatial applications, however, these perturbation maps should be interpreted as pathology-conditioned, hypothesis-generating extrapolations, rather than as direct reconstructions of spatial perturbation ground truth. On prostate slides, virtual perturbations yielded biologically interpretable, pathology-anchored response axes (e.g., stromal *CXCL14* responses in *AR*–*FOXA1*, compartment-biased *KLK3* responses after *ERG* down, and luminal *KLK2* reinforcement after *FOXA1* up), suggesting that image-conditioned perturbation effects can resolve compartment-specific response fields in intact tissue. To our knowledge, this constitutes the first whole-slide, image-only in silico perturbation, opening a path to spatially targeted hypothesis generation and follow-up validation.

Limitations remain. First, spatial-scale perturbation benchmarks are scarce, and current validation still relies on single-cell Perturb-seq supervision rather than paired spatial perturbation ground truth. Second, the current language branch provides only modest gains under coarse sample-level textual supervision and should be viewed as a semantically motivated alignment scaffold rather than a major driver of spot-level prediction accuracy. Third, the proteomics extension was demonstrated on a small co-detection by indexing (CODEX) set, so cross-omics generalisation remains to be quantified systematically, and is now treated as proof-of-concept rather than a fully developed multi-omic result. Fourth, pathway-level recovery remains signal- and ontology-dependent: directional agreement is weaker for low-signal pathways, more fragile in low-contrast comparisons, and less stable in broader or more overlapping pathway collections. Fifth, we have not yet integrated Coladan’s outputs into downstream clinical tasks such as prognosis or subtype stratification, an essential step toward clinical adoption.

Future directions include: (i) establish paired spatial-perturbation datasets—e.g., organoid or animal models with controlled gene edits—to rigorously evaluate causal predictions. (ii) expand the multi-omics branch to additional modalities (spatial proteome, epigenome) and larger cohorts, aiming for a unified spatial-omics foundation model. (iii) exploit synthetic or pseudo-labelled data—generated by Coladan on unlabeled slides—to further scale training and improve robustness. (iv) combine predicted expression maps with histological features for downstream tasks such as grading, subtyping and outcome modeling, quantifying added clinical value.

In summary, Coladan makes high-fidelity, genome-scale spatial omics accessible from standard pathology, at a fraction of the cost of experimental assays. By focusing on well-defined biomedical tasks, it achieves a favourable accuracy-to-compute ratio compared with generic billion-parameter models and can run on a single graphics processing unit (GPU). For cancer discovery, these capabilities enable retrospective cohort mining, biomarker and pathway hypothesis generation, and prioritization of regions or cases for follow-up spatial assays. We expect Coladan to serve as an affordable digital-molecular companion to histopathology, accelerating spatial-omics research and, ultimately, advancing precision medicine.

## Conclusions

We curate Coladan-human3K (~ 3,000 human spatial-transcriptomics profiles; to our knowledge the largest human-only resource) and introduce Coladan, a trimodal whole-slide image-to-omics framework that predicts ~ 60 k genes per spot with calibrated aleatoric and epistemic uncertainty while preserving foundation-model representations. Across 32 held-out 10 × Visium datasets, Coladan improves mean Pearson correlation from 0.230 to 0.431 (1.9 ×), supports pathway-level analysis, and generalizes zero-shot to VisiumHD and Xenium without domain adaptation. Architecturally, Coladan employs a fine-grained mixture-of-experts decoder and Chain-of-Continuous-Thought iterative decoding; enabled by our modular conditioning/training design, we perform a CLS-embedding-only perturbation mode on par with expression-based baselines. Building on this feasibility, we present—to our knowledge—the first image-only in silico perturbation at whole-slide scale, requiring no measured expression at inference and supporting spot-selective, in-situ hypothesis-forming analyses across datasets. Together, the resource, method, and open releases provide a scalable, reproducible route from routine histology to genome-scale spatial expression and a practical platform for exploratory perturbation studies and future multi-omics extensions. 

## Supplementary Information

Additional file 1: Notes S1-S6, Figures S1-S16, Tables S1-S19. Supplementary Notes and figures that complement the analyses in the main text, and tables detailing the benchmarks and training.

Additional file 2: Per-sample manifest of the Coladan-human3K collection—listing source provenance (e.g., GEO), accession (PMID/preprint), sample_id, and assay/technology (e.g., 10 × Visium)—to enable exact reproduction of the dataset.

Additional file 3: Full pathway-level records for the reproducibility benchmark across six comparison pairs and five pathway collections, including method, pair/group, gene panel size, pathway identity, original and predicted NES, signal-strength annotation, same-direction label, and anchor-pathway membership.

Additional file 4: Complete pathway-enrichment results for all experts in the selected interpretable MoE layers (layers 3, 4, 7, and 10) at iteration 3, across Hallmark, KEGG, and Reactome, including top enriched terms and associated enrichment statistics. Only significant layer–expert–library combinations are listed.

## Data Availability

The full model code is hosted at https://github.com/99wzj/Coladan [73]. The trained Coladan model weights and inference demo have been deposited at https://huggingface.co/WangZj99/Coladan [74]. An archival copy of the released source code, together with manuscript-associated source-data tables, analysis scripts and reference workflows, is available in Zenodo: https://doi.org/10.5281/zenodo.20815655 [75]. All data sources are described in the Datasets section and all of them are publicly available. Sample provenance and metadata for the Coladan-human3K cohort are provided in Additional file 2 [4, 8, 13, 33, 34, 76–210]. The processed Coladan-human3K training dataset is publicly available at https://huggingface.co/datasets/WangZj99/Coladan-human3K [211]. Source-data tables and analysis scripts associated with the manuscript analyses are available in the Zenodo archive [75]. Benchmark Visium and VisiumHD datasets were obtained from 10 × Genomics (https://www.10xgenomics.com) [76]. Perturbation-seq data for Norman and Adamson are retrieved from Harvard Dataverse (https://dataverse.harvard.edu/api/access/datafile/6154020 and https://dataverse.harvard.edu/api/access/datafile/6154417, respectively) [38, 39]. The Replogle perturbation dataset is available via the GWPS portal at MIT (https://gwps.wi.mit.edu/) [40]. Single-cell proteomics data for decoder fine-tuning were downloaded from the SCoPE2 resource (https://scope2.slavovlab.net/mass-spec/data) [41]. CODEX multiplex immunofluorescence imaging data are available from The Cancer Imaging Archive (https://www.cancerimagingarchive.net/collection/cptac-gbm/) [42]. TCGA whole-slide H&E images were accessed through the NIH Genomic Data Commons (https://portal.gdc.cancer.gov) [71]. All models used for benchmarking are available on the Hugging Face Hub (https://huggingface.co/).

## Abbreviations

ASW: Average silhouette width
CAMS: Chinese Academy of Medical Sciences
C6: Molecular Signatures Database oncogenic signature gene-set collection
CE: Cross-entropy
CLS: Classification token
CODEX: Co-detection by indexing
CoT: Chain of thought
CRISPRi: CRISPR interference
DDP: Distributed data parallel
FFN: Feed-forward network
GEO: Gene Expression Omnibus
GPU: Graphics processing unit
GSEA: Gene set enrichment analysis
H&E: Hematoxylin and eosin
IQR: Interquartile range
KEGG: Kyoto Encyclopedia of Genes and Genomes
LLM: Large language model
MC: Monte Carlo
MERFISH: Multiplexed error-robust fluorescence in situ hybridization
MLP: Multilayer perceptron
MoE: Mixture of experts
MSE: Mean squared error
NCI: National Cancer Institute
NES: Normalized enrichment score
NLL: Negative log-likelihood
ORB: Oriented FAST and rotated BRIEF
PCC: Pearson correlation coefficient
QC: Quality control
RANSAC: Random sample consensus
ROI: Region of interest
ST: Spatial transcriptomics
TCGA: The Cancer Genome Atlas
UMAP: Uniform manifold approximation and projection
UMI: Unique molecular identifier
ViT: Vision transformer
