# Supplementary material for: Trimodal, uncertainty-guided whole-slide framework for genome-scale spatial expression and image-only virtual perturbation in cancer cohorts
Source: Genome Med. 2026 Jul 14;18:147. doi: 10.1186/s13073-026-01713-y (PMC13651337; doi:10.1186/s13073-026-01713-y)
Supplement: Supplementary file 1 [file 13073_2026_1713_MOESM1_ESM.pdf]

Supplementary Notes

1. Image-to-expression baselines implementation/benchmark details and interpretation of spot-wise versus gene-wise ranking differences.

To complement the pathology-foundation baselines listed in Tables S2–S8, we also benchmarked three directly related image-to-expression methods: DeepSpot, BLEEP, and HisToGene. These methods were selected because they represent three distinct methodological routes that are all relevant to hematoxylin and eosin (H&E)-to-expression prediction, but they do not optimize identical objectives and therefore should not be expected to behave identically across evaluation axes. All three were trained or adapted on the same Coladan-human3K data split used throughout the benchmark, and all predictions were exported as aligned sample-wise expression matrices for the same held-out Visium cohort. As in the main benchmark, the basic image input was always defined as a spot-centered H&E patch, while the patch size followed the native preprocessing of the corresponding backbone or public implementation.

DeepSpot was run in the finalized UNI-based configuration used for this benchmark, with spot_context set to "spot" and a direct supervised prediction head trained on pathology-pretrained image features. We used the same train/test split as in the rest of the benchmark and matched the prediction panel to the corresponding evaluation setting. In our implementation, DeepSpot was more naturally aligned with recovering gene-wise variation across spots than with reconstructing the full within-spot transcriptomic profile. Consistent with this, DeepSpot was the strongest of the three specialized baselines under gene-wise evaluation, although it still remained below CONCH v1.5. By contrast, its spot-wise performance was weaker, and some samples even showed low or negative spot-wise values.

BLEEP was implemented in the finalized CLIP-ViT-based setting used in our benchmark. Following the benchmark script, we froze the image encoder and trained the projection/alignment components on Coladan-human3K, then generated test-time predictions through the model's native joint image–expression embedding and retrieval/imputation pipeline. This design is not optimized for direct gene-by-gene regression; instead, it learns a shared representation for image patches and expression profiles and then imputes from a reference expression bank. In our benchmark, BLEEP was also competitive on spot-wise evaluation, although weaker than HisToGene, but substantially weaker on gene-wise recovery.

HisToGene was implemented in its native patch + coordinate + transformer-based direct prediction formulation and trained on the same Coladan-human3K split used for the benchmark. Compared with retrieval-style methods, HisToGene performs direct prediction from histology patches and spatial coordinates, which again favors recovery of the overall transcriptomic state of individual spots rather than explicit optimization of per-gene spatial variation. In our benchmark, HisToGene was the strongest non-Coladan model on spot-wise evaluation, indicating strong recovery of the overall transcriptomic state of individual spots, but it was substantially weaker on gene-wise recovery.

Taken together, these results indicate that the specialized baselines should not be interpreted as a homogeneous comparison group. Rather, they differ systematically in what aspect of expression recovery they are best aligned to optimize. BLEEP and HisToGene were more competitive on spot-wise evaluation, suggesting stronger recovery of whole-spot transcriptomic states, whereas DeepSpot was more competitive on gene-wise recovery, consistent with a prediction setting more naturally aligned with per-gene variation across spots.

Importantly, however, DeepSpot still remained below CONCH v1.5 on the gene-wise benchmark. We therefore interpret spot-wise and gene-wise metrics as complementary readouts rather than interchangeable summaries of the same property. This is precisely why both evaluation axes were retained in the benchmark.

2. Pathway-level reproducibility across ontologies and signal regimes

To better interpret the pathway-level discrepancies observed in the main text, we expanded the analys is to six fixed sample-pair comparisons, including four primary biological contrasts—Normal_vs_Canc er_Prostate, Parent_vs_Targeted_SpinalCord, Parent_vs_Targeted_Glioblastoma, and Parent_vs_Targeted_ ColorectalCancer—and two low-signal section-level controls—Section1_vs_Section2_Breast_BlockA_40_ 39, Section1_vs_Section2_Breast_BlockA_46_45.

We further evaluated five curated pathway collections: National Cancer Institute (NCI)-Nature_2016, Molecular Signatures Database oncogenic signature gene-set collection (C6), MSigDB_Hallmark_2020, Kyoto Encyclopedia of Genes and Genomes (KEGG)_2021_Human, and Reactome_Pathways_2024. To ensure a fair method comparison, Coladan and CONCH v1.5 were re-evaluated under a matched-gene-universe framework, so that pathway-level comparisons were performed using the same genes for each pair and each panel size (Fig. 2c; Fig. 2e). Because significance-based overlap summaries proved highly threshold-sensitive in the predicted-expression setting, we did not use false discovery rate (FDR)-based detected-pathway counts as the primary evidence in the pathway narrative. Instead, we focused on normalized coverage, same-direction recovery, and normalized enrichment score (NES)-level consistency.

Within this expanded benchmark, we observed that the relative performance of Coladan and CONCH v1.5 depended on both the comparison context and the pathway collection. In particular, when the six comparisons were separated into primary biological contrasts and low-signal controls, pathway directional recovery was generally more stable in the primary comparisons than in the controls, consistent with the idea that weaker baseline biological contrast yields greater enrichment instability (Fig. S10b; Fig. S10c). In addition, under the matched-gene-universe comparison, CONCH v1.5 remained more competitive at small panels in several pathway collections, whereas Coladan became more competitive and often stronger as panel size increased. This indicates that the small-panel discrepancy is consistent with differences in how the two models behave under sparse and low-signal conditions.

To understand why directional mismatch persists, we further stratified pathways by the strength of the original signal (Fig. S10a). Specifically, within the primary biological comparisons, we ranked overlapping pathways by abs (NES_ori) and evaluated same-direction recovery across signal strata. This analysis showed that pathways with weaker original abs (NES) had substantially lower same-direction recovery, whereas pathways with stronger original signals were markedly more stable. Thus, the remaining pathway-level inversions are not uniformly distributed across all biological programs, but are concentrated in weaker-signal regimes.

We also inspected representative discordant anchor pathways directly. These cases were enriched in broader and more overlapping pathway collections, including Hallmark programs such as MYC Targets V1, mTORC1 Signaling, Complement, Oxidative Phosphorylation, Glycolysis, and DNA Repair; C6 oncogenic signatures such as MEK UP.V1 DN, E2F1 UP.V1 DN, MYC UP.V1 UP, and ERBB2 UP.V1 DN; and Reactome

terms related to translation initiation / ribosome-associated programs, as well as signaling pathways such as KIT, NOTCH, and PD-1 (Additional file 3). These pathway categories share three features: they are relatively broad, often contain substantial gene overlap, and frequently represent closely related biological axes. As a result, their enrichment direction is more sensitive to modest ranking perturbations in predicted expression profiles. Taken together, these analyses support a signal- and ontology-dependent view of pathway recovery: Coladan retained pathway-level enrichment signals most reliably when the original pathway signal is stronger, whereas weak-signal pathways and broad or overlapping pathway ontologies remain substantially more challenging.

3. Biological interpretation, control analyses and classification token (CLS)-based extrapolation of displayed prostate perturbation examples.

The displayed perturbation examples were selected by balancing quantitative robustness, regional contrast and biological interpretability, rather than by any single metric alone. *KLK2* and *KLK3* were prioritized as target genes because they are canonical androgen-linked luminal/secretory markers in prostate tissue and prostate cancer [1]. *CLDN3* was prioritized because it provides an epithelial/tight-junction-associated readout in the cancer setting [4]. *FOXA1* was prioritized as a perturbation axis because it is a key determinant of androgen receptor chromatin (*AR*) targeting and transcriptional program in prostate cancer [2], whereas *ERG* was prioritized because it is a lineage-defining oncogenic factor that can disrupt *AR*-mediated differentiation programmes in prostate cancer [3].

Among the main-text single-perturbation examples shown in Fig. 3d, *ERG* down -> *KLK3* and *FOXA1* up -> *KLK2* are the most directionally concordant cases (Table S12). For *ERG* down -> *KLK3* in normal tissue, the regional mean signed Δ values were +0.482 in stroma, +1.100 in immune-rich regions and +0.457 in other mixed regions, whereas the epithelial compartment showed only a small negative shift (−0.129). This pattern is broadly compatible with partial relief of *ERG*-associated suppression of *AR*-linked differentiation output [3], while also indicating that the strongest response is not confined to a purely epithelial domain. For *FOXA1* up -> *KLK2* in cancer tissue, the regional mean signed Δ values were +0.424 in tumour-enriched regions, +1.137 in stroma and +1.273 in immune-rich regions, with only the residual "other" category showing a negative shift (−0.253). This pattern is broadly consistent with positive perturbation of an *AR*-linked luminal/secretory programme [1,2], while also indicating that the response field extends beyond the tumour-enriched compartment into surrounding stromal and immune contexts.

By contrast, *AR* down -> *KLK2* and *ERG* down -> *CLDN3*, shown in Fig. S12e, are better interpreted as regionally polarized response fields than as clean directional validations (Table S12). For *AR* down -> *KLK2* in normal tissue, the epithelial compartment showed a small negative shift (−0.073), but the stromal compartment showed a larger positive shift (+1.029), accompanied by a modest positive immune-rich response (+0.202) and a negative shift in other regions (−0.301). Thus, although *KLK2* is a canonical androgen-responsive luminal marker [1], this example is more naturally interpreted as a spatially redistributed perturbation field than as a uniformly decreasing luminal response. For *ERG* down -> *CLDN3* in cancer tissue, the map was predominantly negative across tumour-enriched (−0.238), stromal (−0.456) and immune-rich (−0.206) regions, with only a near-zero value in other regions (+0.007). Given that *CLDN3* serves here as an epithelial/tight-junction-associated readout [4], this example remains spatially informative but is not presented as a directionally clean positive-control case. Taken together, these four displayed cases illustrate that histology-conditioned perturbation responses can range from directionally concordant patterns to spatially structured but less uniformly interpretable response fields.

An additional supplementary example, *FOXA1* down -> *CLDN3*, is presented as exploratory because its regional behaviour is spatially structured but directionally less clean (Fig. S12e; Table S12). In this case, the regional mean signed Δ values were −0.104 in tumour-enriched regions and −0.480 in stroma, but +0.368 in immune-rich regions and +0.677 in other regions. This mixed-direction pattern does not support a simple single-axis biological interpretation, but it remains informative because it shows that histology-conditioned perturbation responses can redistribute strongly across tissue compartments even when they do not yield a uniformly directional output.

The control analyses were designed to address complementary alternative explanations. Housekeeping controls tested whether similar spatial structure could be reproduced by arbitrary stable genes under the same perturbation condition (Fig. S12d). Matched empirical null analysis tested whether the observed structure could be explained by gene-specific detectability or prediction difficulty after matching on nonzero-locus counts, baseline predicted mean expression and baseline predicted variance (Table S13). In practice, the displayed cases deviated from matched empirical-null expectations to varying degrees, whereas their separation from housekeeping controls was generally broader across multiple perturbation-structure metrics. The clearest empirical-null departure was observed for Cancer | *ERG* down -> *CLDN3*, particularly for compartment-contrast and spatial-autocorrelation-related metrics, whereas the full set of displayed cases remained more consistently separated from the housekeeping background. Exact-entry audit addressed a third possibility, namely that the displayed cases simply replayed already seen perturbation entries from the training resource. Because most displayed driver–target pairs were not found as direct exact-matched entries, these analyses together support interpreting the displayed prostate perturbation maps as anatomically anchored and quantitatively constrained hypothesis-generating outputs, while also clarifying that they do not constitute direct spatial ground-truth validation.

A conceptual advantage of the CLS-only formulation is that it removes the requirement for measured control expression at inference and allows perturbation effects to be queried at exactly the same spatial locus while holding tissue context fixed. This conceptual advantage is also relevant to the reproducible non-zero zero-shot benchmark signal described in the main text. Because the displayed prostate examples are out-of-resource with respect to the perturbation training set, the CLS-only design is relevant not only as an engineering simplification but also as a plausible interface for transferring perturbation knowledge into unseen tissue contexts. Because the same CLS-only interface is used at spatial inference without measured expression, it also provides a natural setting in which such transfer can be tested directly in tissue. A further plausible interpretation is that this extrapolative behaviour is supported not only by perturbation supervision itself but also by the iterative completion mechanism used during baseline gene-expression prediction, in which high-confidence gene representations from earlier decoding rounds are fed back into later rounds under the constraint of a shared CLS embedding. We therefore regard this as a plausible explanation for out-of-resource perturbation extrapolation rather than as a directly established mechanism.

4. Comparison of uncertainty formulations for reliability-aware spatial prioritization

To assess how uncertainty should be represented for image-to-transcriptome prediction, we compared three uncertainty formulations: a cross-entropy (CE)-head entropy baseline, a point-wise Gaussian model, and an interval-aware Gaussian model. These analyses were designed to evaluate both predictive fidelity and whether the resulting uncertainty signals could support practical prioritization of spatial regions by reliability.

The three formulations differ in how they encode predictive uncertainty. In the CE-head formulation, the decoder predicts discretized expression categories and uncertainty is summarized by the entropy of the categorical output distribution. In the point-wise Gaussian formulation, each discretized target is represented by a single value in the bin-based expression space, and the model predicts both a mean and a data-dependent standard deviation. In the interval-aware Gaussian formulation, each discretized target is instead interpreted as belonging to the interval defined by its bin boundaries, so that uncertainty is evaluated with respect to the full bin interval rather than a single representative point.

We first compared predictive fidelity across samples. Both Gaussian formulations substantially outperformed the CE-head baseline in average spot-wise Pearson correlation (Fig. S13a), indicating that although CE-style uncertainty provides an interpretable confidence proxy, it comes at a substantial loss of predictive accuracy. Consistent with this, CE-head entropy still showed a strong negative association with sample-level accuracy (Pearson $r = -0.75$; Fig. S13c), confirming that higher entropy corresponds to less reliable predictions even though the overall model performance is weaker.

We next examined whether Gaussian uncertainty can be used directly to prioritize spatial spots by reliability. To do so, we ranked spots by predicted uncertainty and recalculated average spot-wise correlations after retaining only the lowest-uncertainty 25%, 50%, 75%, or all spots. Both Gaussian formulations showed the same qualitative behavior: restricting analysis to lower-uncertainty spots consistently enriched for higher-fidelity regions (Fig. S13b). At 25% retained spots, the average spot-wise Pearson reached 0.477 for the point-wise Gaussian model and 0.478 for the interval-aware Gaussian model, indicating that the two Gaussian formulations were very similar in their ability to identify reliable regions.

We additionally evaluated coverage calibration at nominal coverage levels of 50%, 80%, and 90% (Fig. S13d,e). Here the distinction between the two Gaussian formulations became clearer. The interval-aware Gaussian achieved higher empirical coverage at all three nominal levels, indicating a more conservative uncertainty profile, but this came at the cost of substantially wider prediction intervals. In contrast, the point-wise Gaussian produced narrower and sharper intervals, but with lower empirical coverage. Thus, the interval-aware Gaussian provides a more conservative coverage profile, whereas the point-wise Gaussian yields more compact uncertainty estimates.

Together, these results support three conclusions. First, uncertainty is directly useful for ranking spatial regions by expected reliability. Second, the CE-head baseline provides an informative but substantially weaker alternative, showing that uncertainty quality and predictive fidelity must be assessed jointly. Third, the point-wise and interval-aware Gaussian formulations behave similarly in ranking reliable regions, but differ in uncertainty style: the interval-aware Gaussian is more conservative, whereas the point-wise Gaussian is sharper. In practice, low-uncertainty regions can therefore be prioritized first for interpretation, visualization, and candidate-region selection, while the choice between Gaussian formulations mainly determines whether one prefers broader conservative coverage or tighter predictive intervals.

5. Quantitative dissection of mixture-of-experts (MoE) routing organization

We analyzed routing organization across all 12 decoder layers and across the three Coconut refinement iterations.

Because all MoE analyses in this study used top-1 routing, each valid gene token contributed exactly one routed-expert assignment. During evaluation, routing statistics were recorded for every valid gene token, including the selected routed expert under top-1 routing, the full gate softmax distribution over routed experts, and the relative contribution of the shared and routed branches. These quantities were first summarized at the sample level for each decoder layer and Coconut iteration and then aggregated across samples for global analyses. For tissue-stratified analyses, the same statistics were recomputed after grouping tokens by tissue.

Because the final Coconut iteration is most directly linked to the final gene-expression output, iteration 3 was used for downstream expert-role analyses. Across the decoder, routing showed a clear layer-dependent organization rather than uniform specialization (Fig. 5e). A subset of intermediate and mid-to-late layers retained higher routed-expert diversity, whereas several other layers showed more concentrated routed usage together with stronger shared-path contribution (Fig. 5e). On this basis, layers 3, 4, 7, and 10 were selected for downstream gene- and pathway-level analyses, as they showed the clearest combination of higher effective expert number and lower maximum expert usage (Fig. 5e; Fig. S15b). By contrast, layers with lower effective expert number and more concentrated routing were interpreted more conservatively as consolidation- or integration-dominated stages. This interpretation was further supported by exploratory deconsolidation attempts targeted to later layers, including gate-regularization adjustment, selective unfreezing of routed components, and mild gate perturbation. These interventions produced only local changes and did not consistently restore broad late-layer routed diversity, supporting a structural refinement interpretation rather than a simple training-failure explanation.

In parallel with routed-expert usage, we quantified the relative contribution of the always-active shared branch. Shared-path contribution remained non-zero throughout the decoder and became more prominent in several later layers (Fig. 5e). This pattern supports interpreting later-layer concentration jointly with increasing shared-path contribution, consistent with a transition from broader routed specialization toward more shared integrative refinement.

To assess tissue dependence, we repeated the same aggregation procedure within tissue groups. At this level of aggregation, dominant routing patterns were broadly conserved across tissues rather than extensively reconfigured in a tissue-specific manner (Fig. S14). Tissue-stratified enrichment analyses likewise showed that the major expert-associated biological programs were largely preserved across tissues. These observations suggest that the dominant routed programs correspond to broadly shared cellular modules rather than strong tissue-specific rewiring of the decoder.

We next examined the biological annotatability of selected routed components using an expert-centric enrichment analysis. For each selected layer, we collected the genes most frequently assigned to individual experts at iteration 3 and performed pathway enrichment using Hallmark, KEGG, and Reactome. This analysis identified reproducible expert-associated annotations, including membrane trafficking and RAB geranylgeranylation (layer 3, expert 4; Fig. S15c), oxidative phosphorylation and respiratory electron transport (layer 7, expert 1; Fig. 5c), proteostasis and antigen-processing programs (layer 7, expert 0; Fig. S15a), and signaling-related regulation including NOTCH- and MYC-associated pathways (layer 4, expert 7; Fig. S15d). A complete reference table listing the top enriched Hallmark, KEGG, and Reactome terms for experts in the selected layers is provided in Additional file 4. Only layer–expert–library combinations that reached significance under the current threshold are listed; absence from the table therefore indicates that no significant

enrichment was detected under the current threshold, rather than selective omission.

Because expert-level enrichment indicates whether selected routed components can be annotated by existing pathways but does not by itself establish pathway-wide routing organization, we further performed a complementary pathway-centric matched-null analysis. For each gene and decoder layer, selected routed-expert assignments from the final refinement iteration were aggregated into gene-level selected-expert profiles. For each curated Hallmark, KEGG, or Reactome pathway, we computed pathway-to-routing coherence as the mean pairwise cosine similarity among the selected-expert profiles of pathway member genes. The observed coherence was compared with 500 random gene sets matched for both pathway size and routing-count coverage. This analysis asked whether genes within the same curated pathway shared more similar selected-expert profiles than expected under matched random controls.

The matched-null analysis showed that pathway-to-routing coherence was layer-dependent rather than uniformly present across the decoder. Positive coherence was concentrated in a subset of layers, whereas other layers showed weak, near-null, or negative coherence relative to matched random controls. This result indicates that existing curated pathways explain part, but not all, of the learned selected-expert assignment structure. Thus, the expert-centric enrichment and pathway-centric matched-null analyses support complementary interpretations: selected routed components can be associated with known biological annotations, and a subset of the broader routing structure is concordant with existing curated pathways. However, these analyses do not imply that individual experts directly correspond to biological pathway modules or that all routing roles are biologically explained by current pathway collections.

Together, these analyses indicate that MoE routing in Coladan is organized in a layer-dependent manner, with clearer routed specialization in a subset of decoder layers and stronger shared-path integration in others. The routed branch should be interpreted primarily as a computational routing mechanism, but part of its learned assignment structure is associated with known pathway-level biological organization. Other routing roles may reflect computational specialization, biological structure not captured by current pathway annotations, or both.

6. Preliminary extension to spatial proteomics

We purposely designed Coladan to be both multimodal and multiomic: the shared patch and slide encoders remain frozen during downstream adaptation, and the learned image representations can in principle support multiple omics-specific decoder branches. As a preliminary proof-of-concept, we instantiated this flexibility by adapting the transcript decoder into a protein decoder and pairing it with the same frozen image backbone (Fig. S5a).

Specifically, we fine-tuned the decoder branch on a published single-cell proteomics dataset comprising approximately 1,500 cells5, and then re-inserted the resulting protein branch into the Coladan pipeline to predict spatial protein abundance from co-detection by indexing (CODEX)-derived H&E images6. Although the overall cell-level Spearman correlations were modest, likely reflecting the limited scale of the current fine-tuning data, several representative proteins already showed encouraging spatial recovery, including GFAP ($r \approx 0.39$; Fig. S5b) and OLIG2 ($r \approx 0.26$; Fig. S5b). These results suggest that the shared morphology-conditioned representation can, in principle, support additional omics-specific heads beyond RNA.

We note that these correlations were computed across all spots/cells in the evaluation set, rather than using the spot-level gene-expression metric reported for transcriptomic prediction in the main text. Given the limited size and marker coverage of the currently available paired spatial proteomics data, we present this analysis as a preliminary proof-of-concept rather than a fully validated multi-omic result.

Supplement Reference:

1. Thorek DLJ, Evans MJ, Carlsson SV, Ulmert D, Lilja H. Prostate-specific kallikrein-related peptidases and their relation to prostate cancer biology and detection: established relevance and emerging roles. Thromb Haemost. 2013;110:484–92. https://doi.org/10.1160/TH13-04-0275

2. Jin H-J, Zhao JC, Wu L, Kim J, Yu J. Cooperativity and equilibrium with FOXA1 define the and rogen receptor transcriptional program. Nat Commun. Nature Publishing Group; 2014;5:3972. https://doi.org/10.1038/ncomms4972

3. Yu J, Yu J, Mani R-S, Cao Q, Brenner CJ, Cao X, et al. An integrated network of androgen receptor, polycomb, and TMPRSS2-ERG gene fusions in prostate cancer progression. Cancer Cell. Elsevier; 2010;17:443–54. https://doi.org/10.1016/j.ccr.2010.03.018

4. Büyücek S, Schraps N, Menz A, Lutz F, Chirico V, Viehweger F, et al. Prevalence and clinical significance of claudin-3 expression in cancer: a tissue microarray study on 14,966 tumor samples. Biomarker Res. 2024;12:154. https://doi.org/10.1186/s40364-024-00702-w

5. Specht H, Emmott E, Petelski AA, Huffman RG, Perlman DH, Serra M, et al. Single-cell proteomic and transcriptomic analysis of macrophage heterogeneity using SCoPE2. Genome Biol. 2021;22:50. https://doi.org/10.1186/s13059-021-02267-5

6. Liu J, Cao S, Imbach KJ, Gritsenko MA, Lih T-SM, Kyle JE, et al. Multi-scale signaling and tu mor evolution in high-grade gliomas. Cancer Cell. Elsevier; 2024;42:1217-1238.e19. https://doi.org/10.1016/j.ccell.2024.06.004

Supplementary Figures

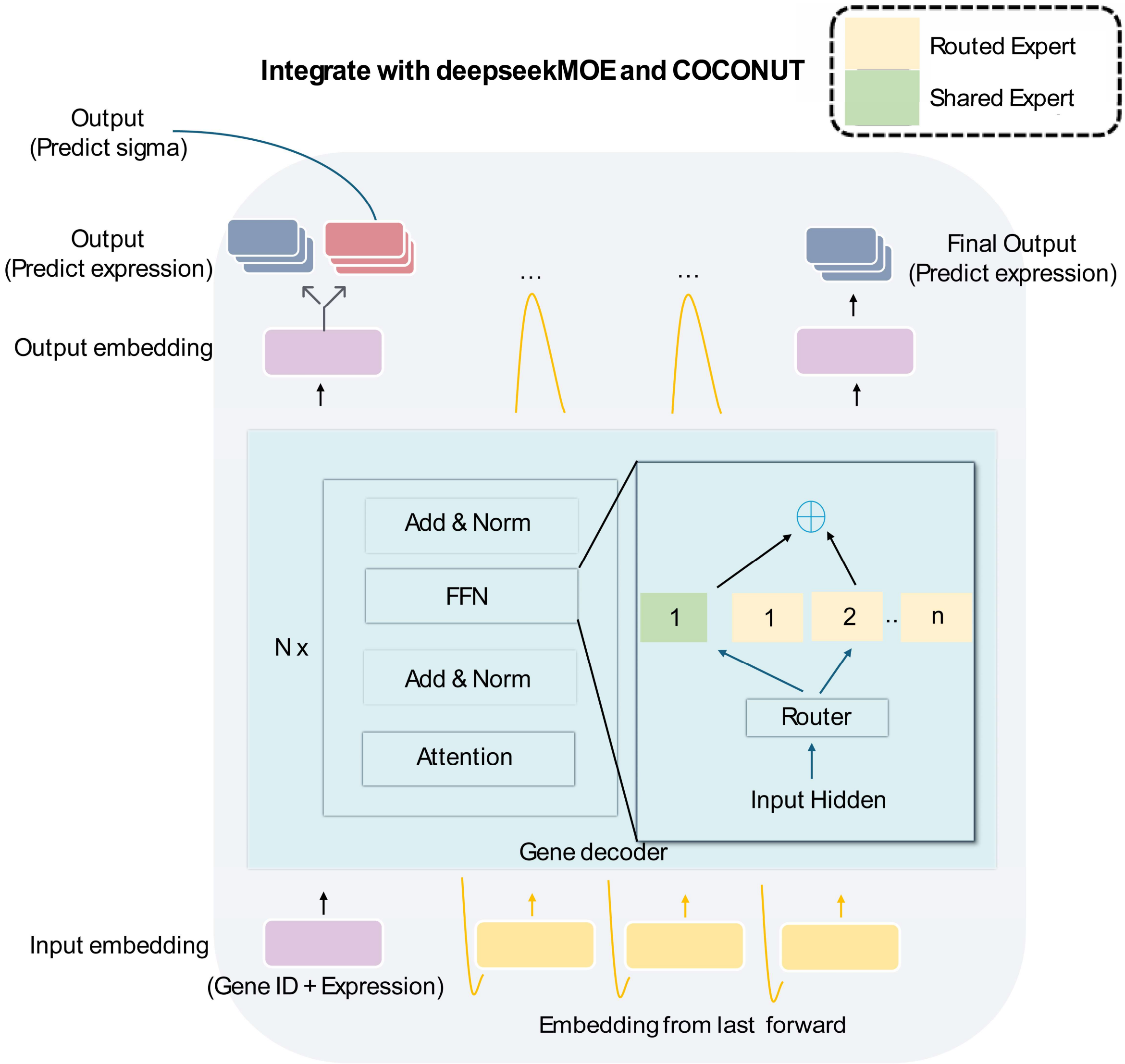

Fig. S1 | Gene decoder layer with MoE and Coconut iteration.

This schematic illustrates the gene decoder layer in Coladan, which follows the standard transformer architecture—self-attention, add & norm, and a feed-forward network (FFN)—stacked N times. In our design, each FFN is replaced by a fine-grained mixture-of-experts (MoE) module: a router examines the input hidden state to dynamically activate a small set of “routed” experts alongside a shared expert pool. The outputs of the active experts are weighted and summed, producing the layer’s output hidden representation. After the final layer, two heads project the output embedding to predict per-gene expression values and aleatoric uncertainty (σ). Crucially, these outputs are then fed back—along with high-confidence prediction positions—into the input embeddings for subsequent decoding passes, enabling multi-step latent reasoning and iterative refinement (the Coconut mechanism).

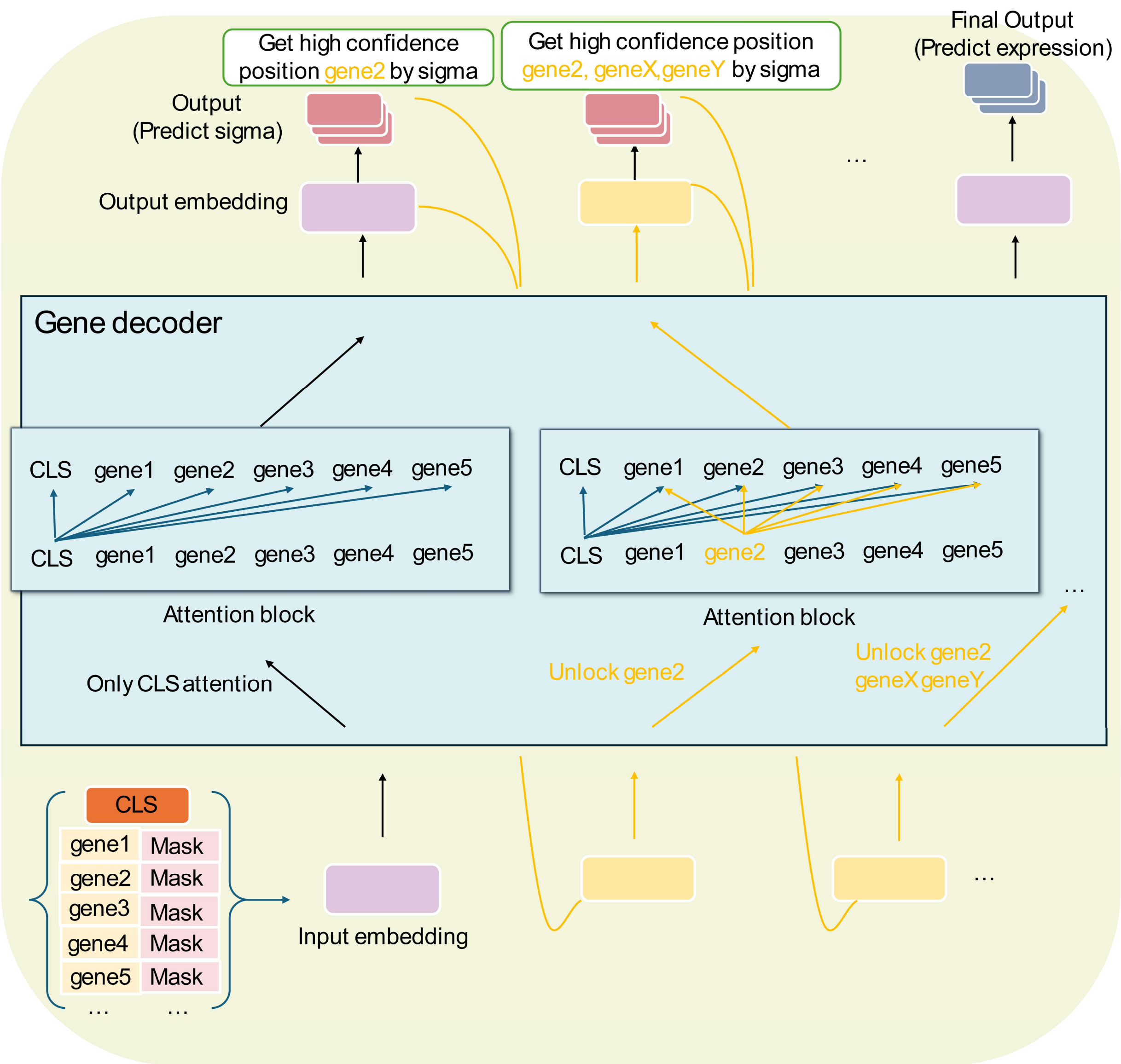

Fig. S2 | Coconut: uncertainty-guided iterative decoding.

All gene tokens remain masked throughout the process, and only a single classification token (CLS) embedding is provided as input to the decoder. In the first pass, the gene decoder produces output embedding along with per-gene aleatoric uncertainties (σ). For the next iteration, we feed that previous output embedding back as the new input embedding, the attention mechanism is modified to "unlock" these high-confidence positions—enabling all gene tokens to attend preferentially to those high-confidence positions. This uncertainty-guided attention adjustment and embedding feedback cycle repeats for C iterations, with each round refining the expression estimates. The final output embedding is projected to the spot's genome-scale expression profile.

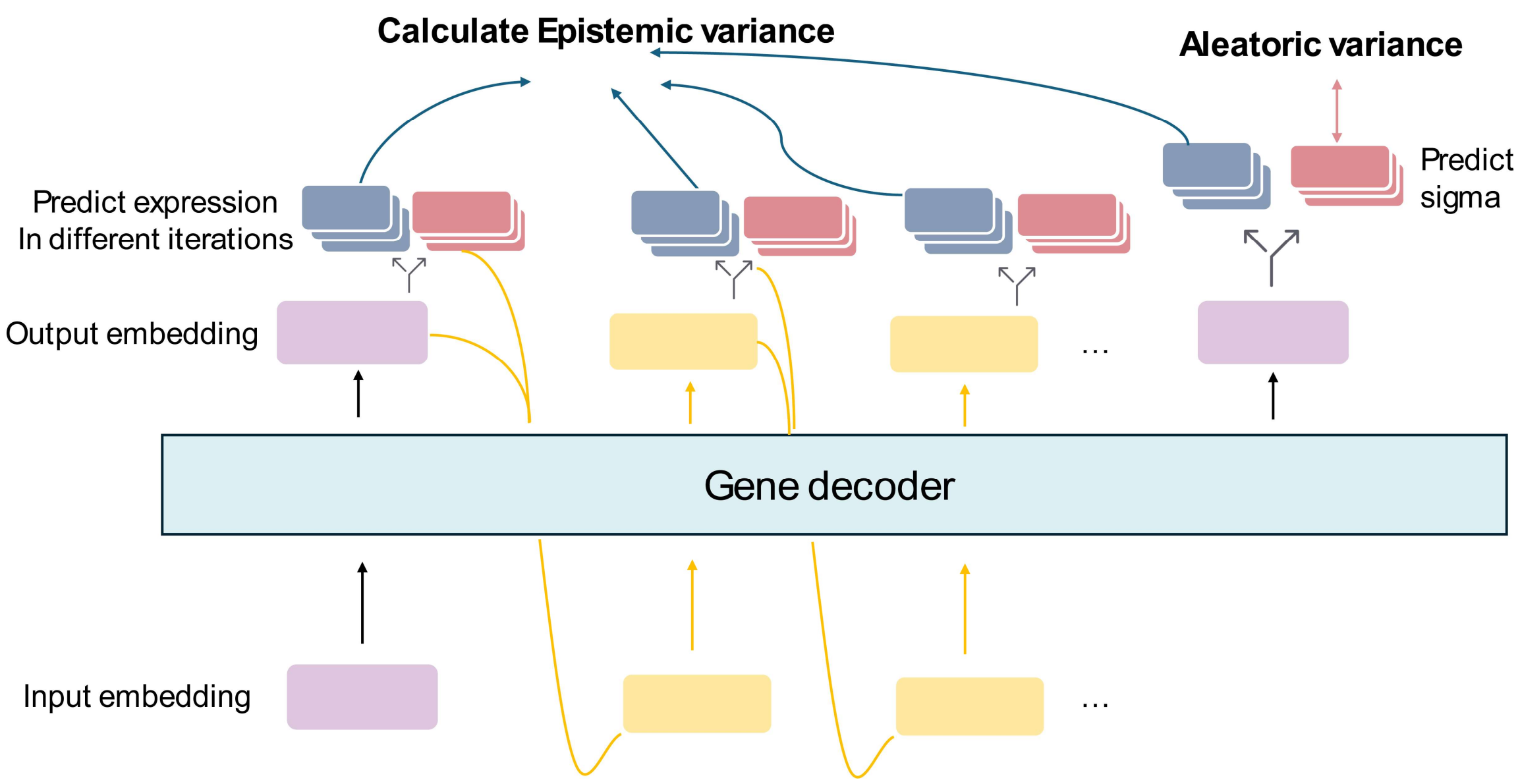

Fig. S3 | Schematic of dual-variance estimation.

a, In each Coconut iteration, the gene decoder outputs both a mean prediction and an aleatoric uncertainty (σ). The aleatoric variance is taken directly from final σ values, while the epistemic variance is computed as the variance of mean predictions across multiple Coconut passes.

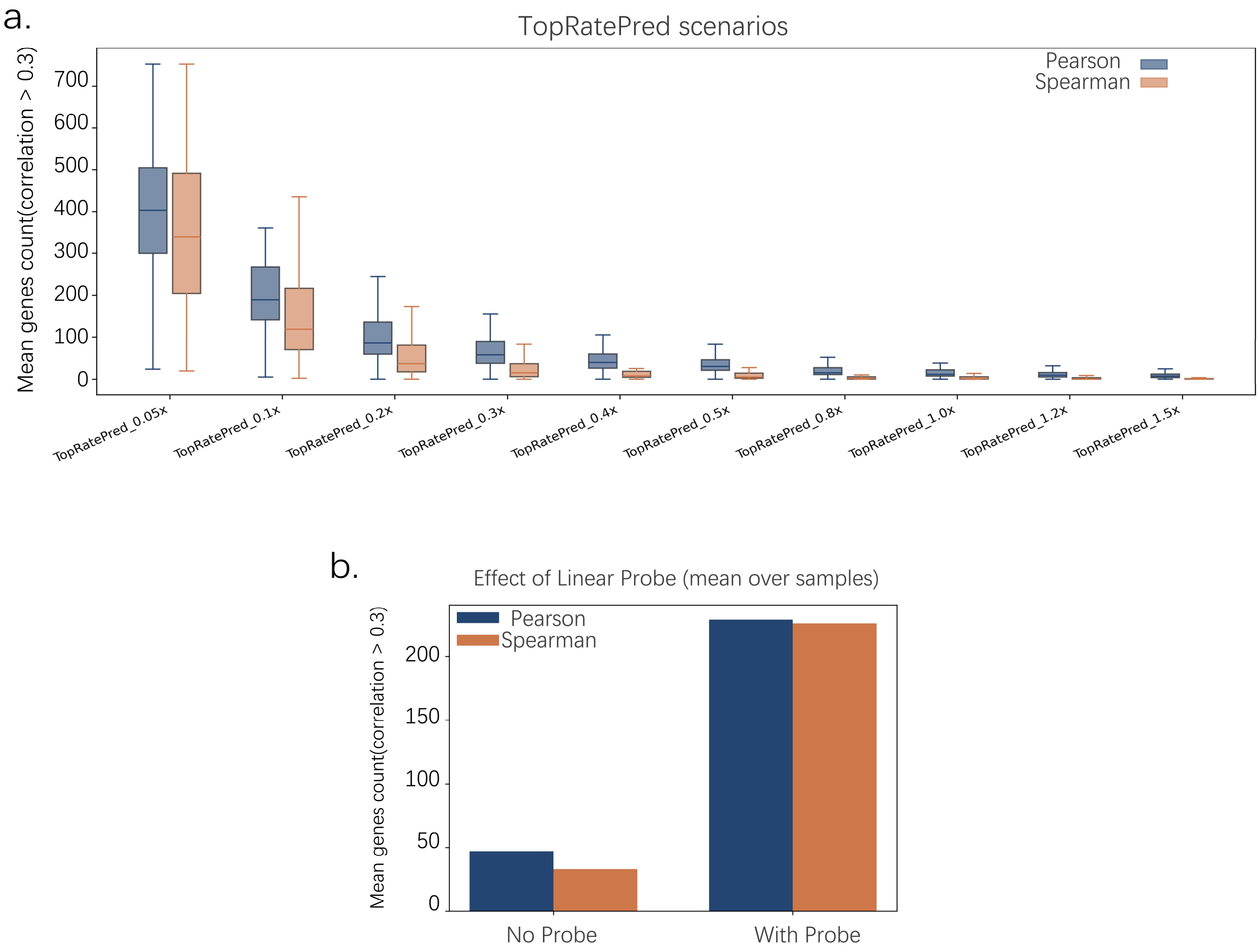

Fig. S4 | Deployment-oriented usability: TopRatePred targeting, and linear-probe gains

a, TopRatePred scenarios. For each sample and each gene, all spots are ranked by the Coladan's predicted expression and only a top-ranked subset is retained at several keep-rates (TopRatePred_0.05× … TopRatePred_1.5×, factors are applied relative to each gene's prevalence, see Methods). Pearson and Spearman are then computed restricted to the retained spots, and we count the number of genes with correlation > 0.3. Boxes summarise the distribution across samples (center line: median; box: interquartile range; whiskers: non-outlier range). As the retained proportion increases from 0.05× to 1.5×, the number of genes exceeding the threshold decreases monotonically, indicating that high-confidence "pred-high" loci carry most of the usable signal for gene-level correlations.

b, Effect of a linear probe (computed on all loci). Mean number of genes per sample with Pearson and Spearman exceeding 0.3 when correlations are computed on all loci. "No Probe" uses the base model alone; "With Probe" adds a lightweight linear probe trained to classify expressed vs. non-expressed from image-derived CLS embeddings.

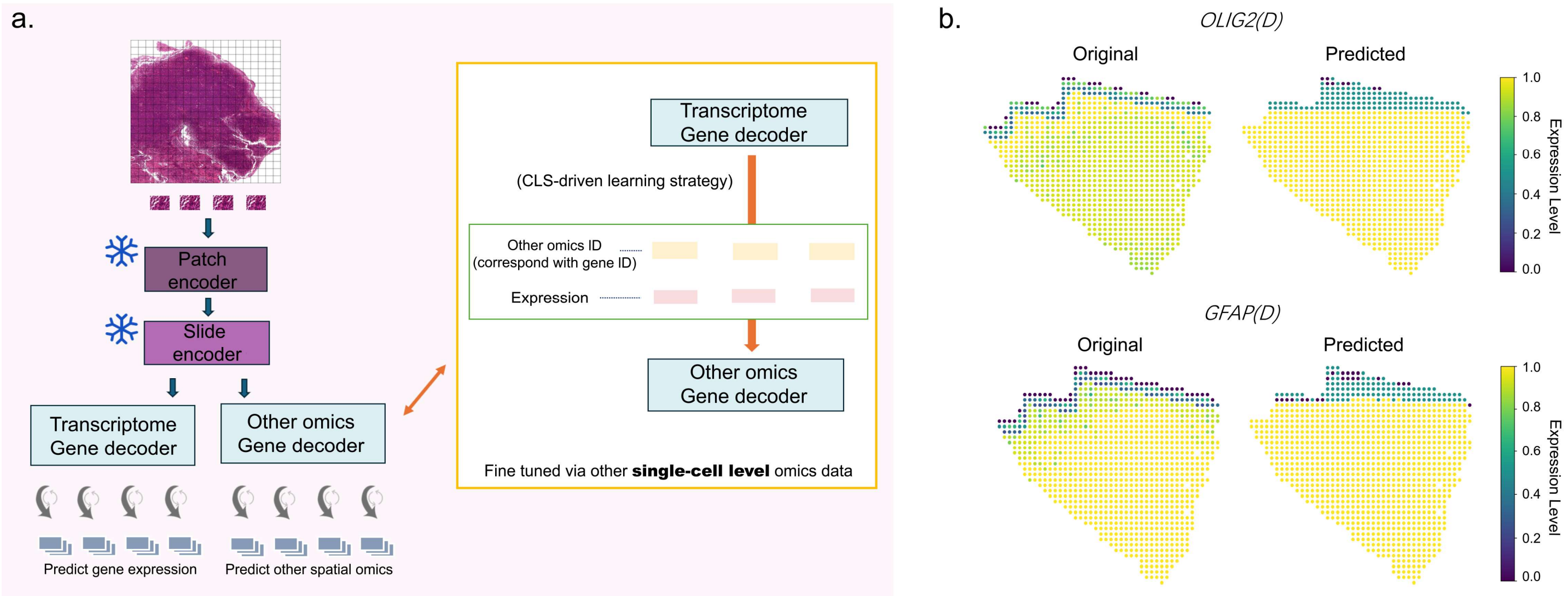

Fig. S5 | Architecture for parallel RNA and other-omics decoding and spatial proteomics prediction via fine-tuned protein branch supplement

a, Architecture for parallel RNA and other-omics decoding. The shared patch and slide encoders (frozen) feed a common embedding into two separate gene-decoder branches—one trained on spatial transcriptomics, the other fine-tuned on single-cell proteomics—enabling simultaneous prediction of RNA and protein abundance from H&E.

b, Spatially resolved prediction of OLIG2 (top) and GFAP (bottom) protein abundance on a CODEX H&E section after fine-tuning the protein decoder. Each spot is colored by normalized expression level (purple = 0, yellow = 1). Measured OLIG2 and GFAP protein levels from CODEX immunofluorescence (left) and Coladan's predicted OLIG2 and GFAP abundance (right). Although overall performance remains modest, achieving such fidelity with minimal fine-tuning underscores the model's potential.

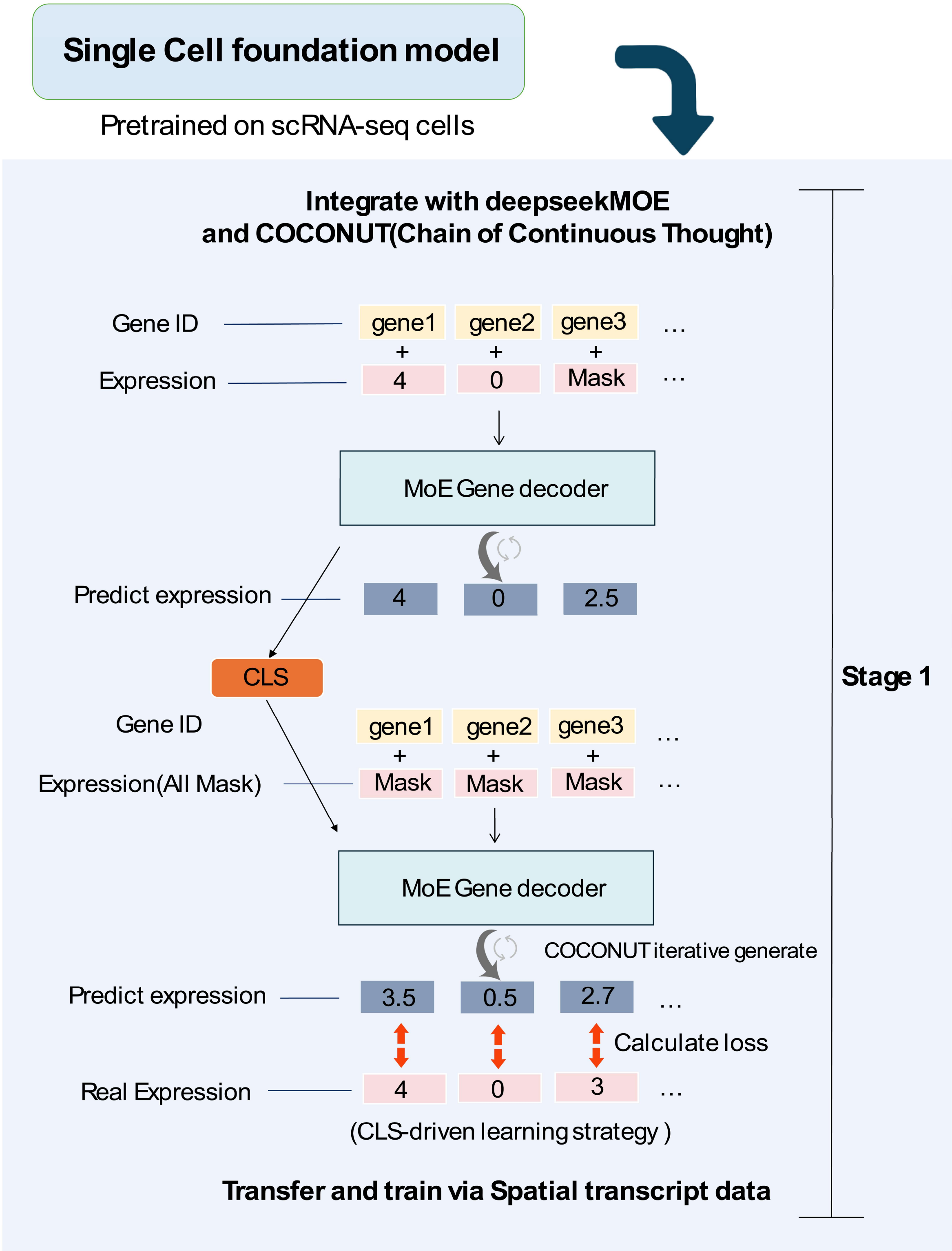

Fig. S6 | Details of training stage 1

A single-cell foundation model pretrained on single-cell RNA sequencing (scRNA-seq) cells was extended with a DeepSeekMoE gene decoder and Coconut iterative generation. Gene IDs and masked expression tokens were first decoded to predict expression values, and the resulting CLS representation was further used to guide all-mask expression reconstruction. The predicted expression profiles were compared with measured spatial transcriptomic expression to compute the training loss, enabling CLS-driven transfer from single-cell transcriptomes to spatial transcriptomic modeling.

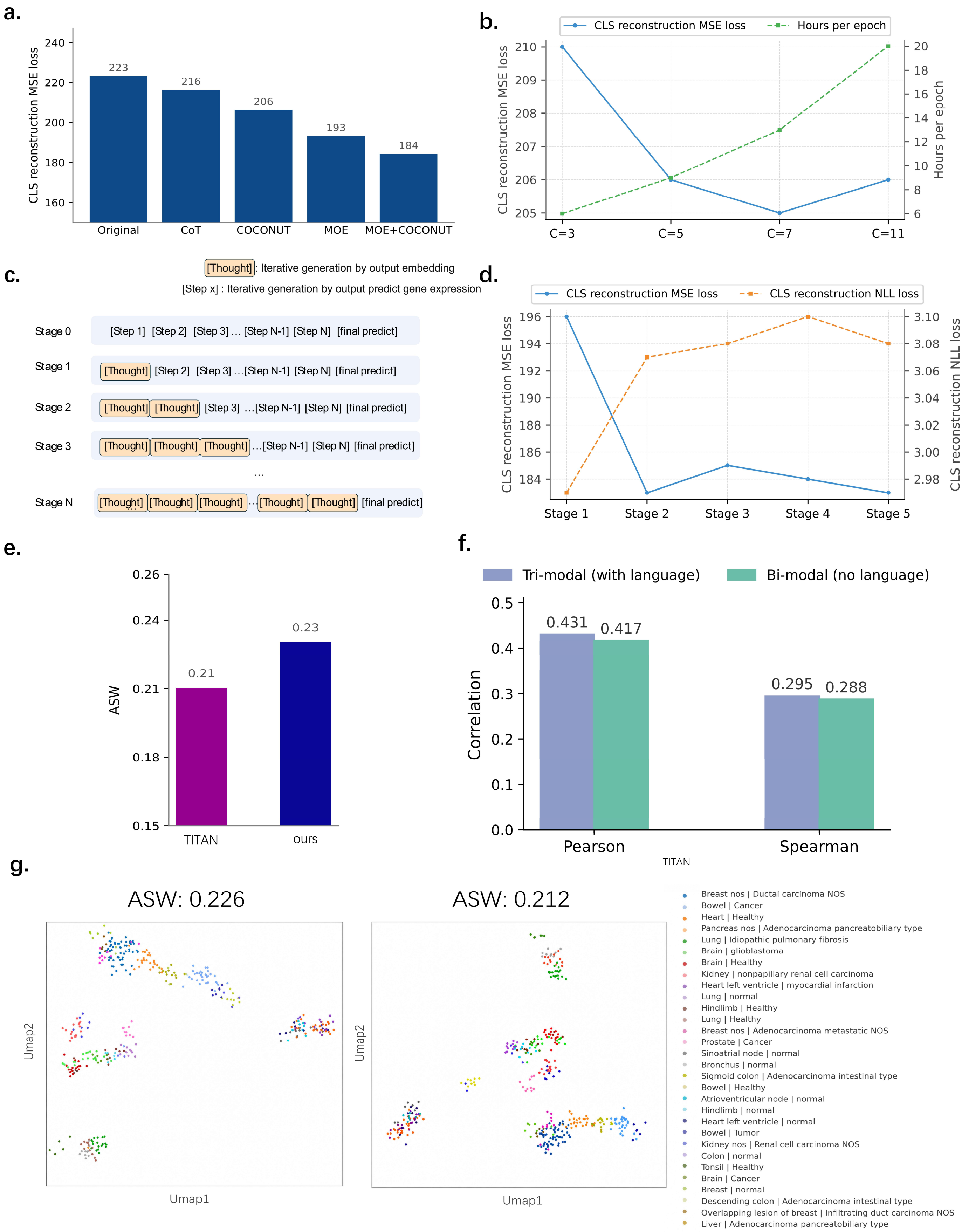

Fig. S7 | Ablation studies of core architectural components and hyperparameters.

a, We evaluated five gene-decoder variants on the CLS reconstruction task by measuring mean squared error (MSE) loss: the unmodified pretrained model (original scGPT, MSE = 223); uncertainty-guided chain-of-thought (CoT) without latent-state feedback ("CoT", see Methods, MSE = 216); Chain-of-Continuous-Thought iteration without MoE routing ("Coconut", MSE = 206); fine-grained DeepSeek-style routing only ("MoE only", MSE = 193); and our combined MoE + Coconut design, which achieves the lowest MSE of 184. Definitions of each variant are detailed in Methods.
b, Trade-off between Coconut iterations (C) and compute cost. As k increases from 3 to 11, CLS reconstruction MSE loss (blue line, left axis) decreases to a minimum at C = 7, but the gain from C = 5 to C = 7 is marginal (206 → 205), while the epoch runtime (green dashed line, right axis) increases substantially. We therefore adopt C = 5 as the default.
c, Coconut training iteration schedule. Each "Step x" denotes a CoT pass(see Methods) without latent feedback; each "Thought" marker indicates a full Coconut iteration that feeds back the previous output embedding and high confidence positions. Over successive stages, an increasing number of "Thought" passes are applied before the final training stage.
d, Reconstruction MSE and Gaussian negative log-likelihood (NLL) loss across Coconut training stages. The x-axis denotes successive Coconut training stages (Stage 1 through Stage k); the left y-axis shows CLS reconstruction mean squared error (MSE, blue line); the right y-axis shows the Gaussian negative log-likelihood (NLL) loss (orange line). Although the NLL loss increases across stages, the reconstruction MSE steadily decreases.
e, average silhouette width (ASW) computed on slide CLS embeddings increases from 0.21 (TITAN) to 0.23 (Coladan), indicating that Coladan preserve foundation-model capacity.
f, Effect of the language modality on genome-scale decoding. Median gene-wise correlations on the benchmark Visium cohort (matched data/compute): tri-modal Coladan (with language) attains Pearson/Spearman = 0.431/0.295, versus 0.417/0.288 for the bi-modal variant (no language). Language-conditioned training supplies complementary semantics that improve cross-modal alignment and prediction accuracy.
g, uniform manifold approximation and projection (UMAP) visualization of slide-level embeddings, colored by tissue type and disease status. Each point represents the embedding of one slide, and colors encode "Tissue | Status" (e.g. "breast | normal", "lung | cancer", "heart | healthy", etc.). Higher average silhouette width (ASW) indicates better separation of biological classes in the embedding space. Left (ASW = 0.226): Trimodal Coladan embeddings integrating H&E image features, slide context, and spatial gene-expression information. Right (ASW = 0.212): Bimodal embeddings (from TITAN, no spatial gene modality information). Labels are reproduced from the original source annotations without re-labelling. Note that tumor-adjacent "Normal" may differ molecularly from "Healthy."

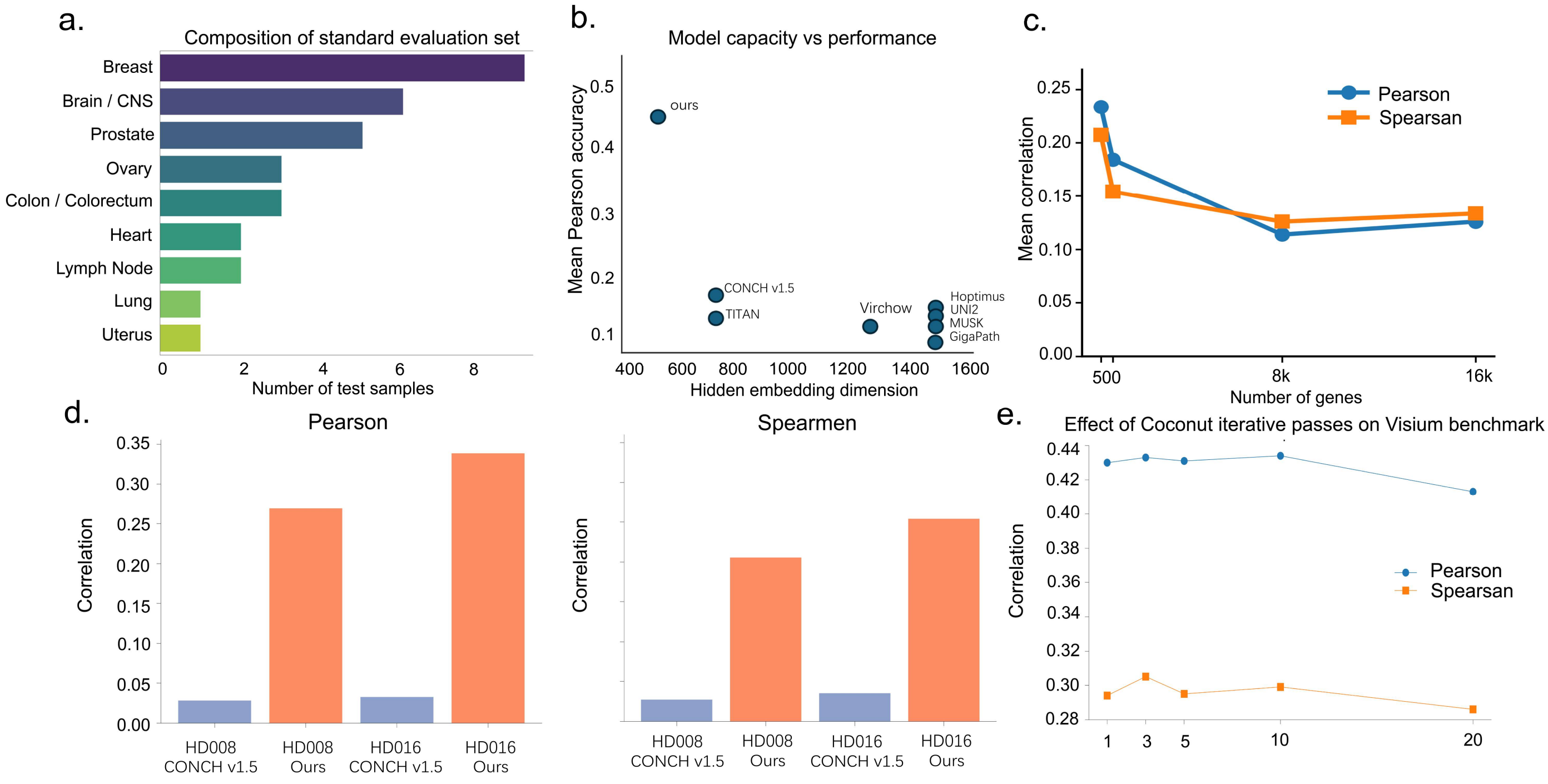

Fig. S8 | Model capacity, scaling and performance.

a, Tissue composition of the 32 standard 10x Genomics Visium evaluation slides used throughout our benchmarking (breast, brain/CNS, prostate, ovary, colon/colorectum, heart, lymph node, lung, uterus).

b, Scatter of hidden-embedding dimensionality versus mean spot-wise Pearson correlation: Coladan (512-D) achieves far higher accuracy than larger backbones (UNI2 768-D, Virchow 1280-D, TITAN 768-D, MUSK 1536-D, H-Optimus-0 1536-D, GigaPath 1536-D, CONCH v1.5 768-D), demonstrating that our compact latent space suffices for genome-scale prediction.

c, Effect of gene-panel size on accuracy. Mean spot-wise Pearson (blue) and Spearman (orange) correlations under matched data/architecture/compute while varying the number of predicted genes (500, 1k, 8k, 16k).

d, Bar plots show spot-wise Pearson and Spearman correlations for two standard Visium-HD binning levels: HD 008 (8 µm bins, directory square_008um) and HD 016 (16 µm bins, square_016um). Coladan (orange) greatly exceeds the fine-tuned CONCH v1.5 baseline (blue) at both spatial resolutions.

e, Spot-wise Pearson (blue circles) and Spearman (orange squares) were evaluated after 1, 3, 5, 10 and 20 Coconut inference passes.

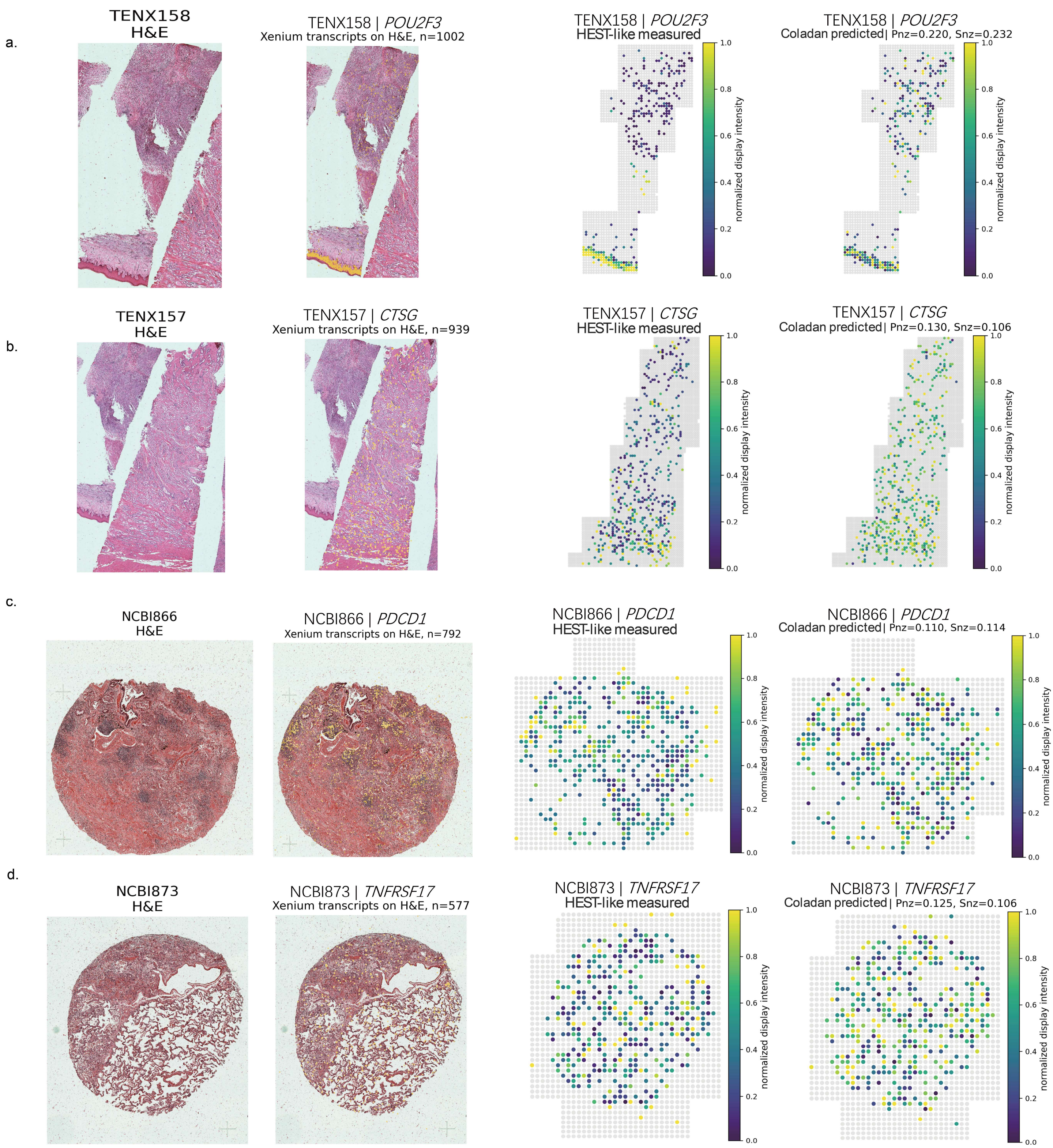

Figure S9 | Representative measured and predicted spatial maps on spot-level Xenium.

Representative sample-gene pairs were selected from the same held-out Xenium slides evaluated in Fig. 4c–d. Each row shows one sample-gene pair: NCBI866–*PDCD1*, NCBI873–*TNFRSF17*, TENX157–

CTSG, and TENX158–*POU2F3*. The first column shows the H&E image. The second column shows native Xenium transcript locations overlaid on H&E as a molecule-level spatial reference. The third column shows processed Xenium-derived measured spot-level Bin50 expression, corresponding to the benchmark target. The fourth column shows Coladan-predicted spot-level Bin50 expression on the same processed units. Grey spots indicate loci with zero measured expression for the displayed gene, whereas colored spots indicate measured non-zero loci. Pnz and Snz denote Pearson and Spearman correlations computed on measured non-zero loci using raw unnormalized Bin50 values.

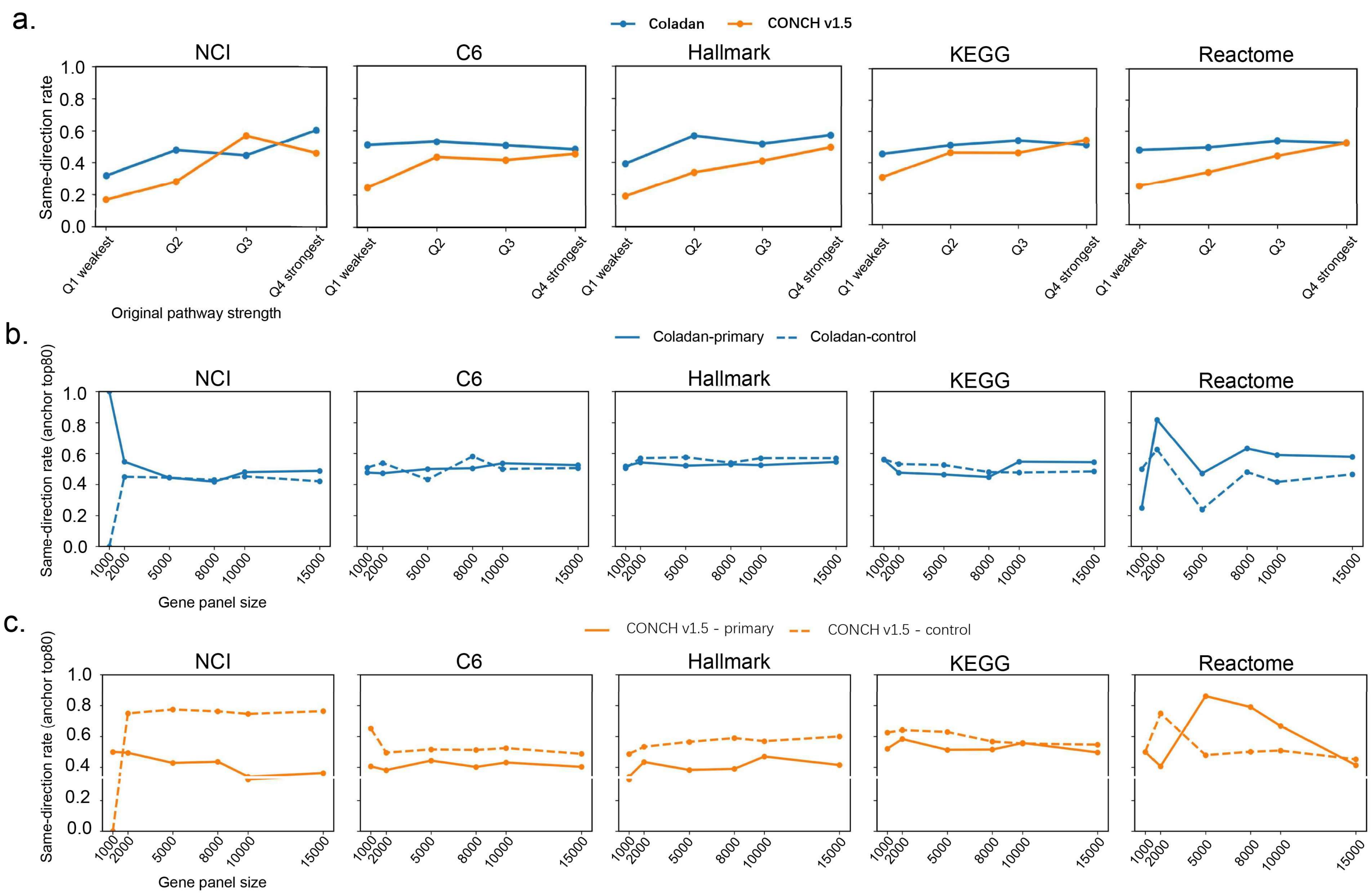

Fig. S10 | Supplementary analysis of pathway-level directional recovery.

a, Anchor-pathway coverage across pathway collections and gene panel sizes. Pathways were stratified into quartiles according to the strength of the original signal (abs(NES_ori)), from Q1 weakest to Q4 strongest. For each pathway collection, curves show the mean same-direction rate for Coladan (blue) and CONCH v1.5 (orange). Stronger original pathways are generally more stable, whereas weaker pathways show lower directional agreement and contribute disproportionately to the remaining pathway-level inversions.

b, Coladan pathway-level recovery in primary versus control comparisons. For each pathway collection (NCI, C6, Hallmark, KEGG, and Reactome), curves show the mean same-direction rate among anchor top80 pathways across gene panel sizes. Solid blue lines indicate the four primary biological comparisons; dashed blue lines indicate the two low-signal section-level controls. Pathway directional recovery is generally more stable in the primary comparisons than in the controls, consistent with stronger underlying biological contrast.

c, CONCH v1.5 pathway-level recovery in primary versus control comparisons. For each pathway collection (NCI, C6, Hallmark, KEGG, and Reactome), curves show the mean same-direction rate among anchor top80 pathways across gene panel sizes. Solid orange lines indicate the four primary biological comparisons; dashed orange lines indicate the two low-signal section-level controls. As in Coladan, directional stability depends on both the pathway collection and the comparison context, with lower stability in the low-signal controls.

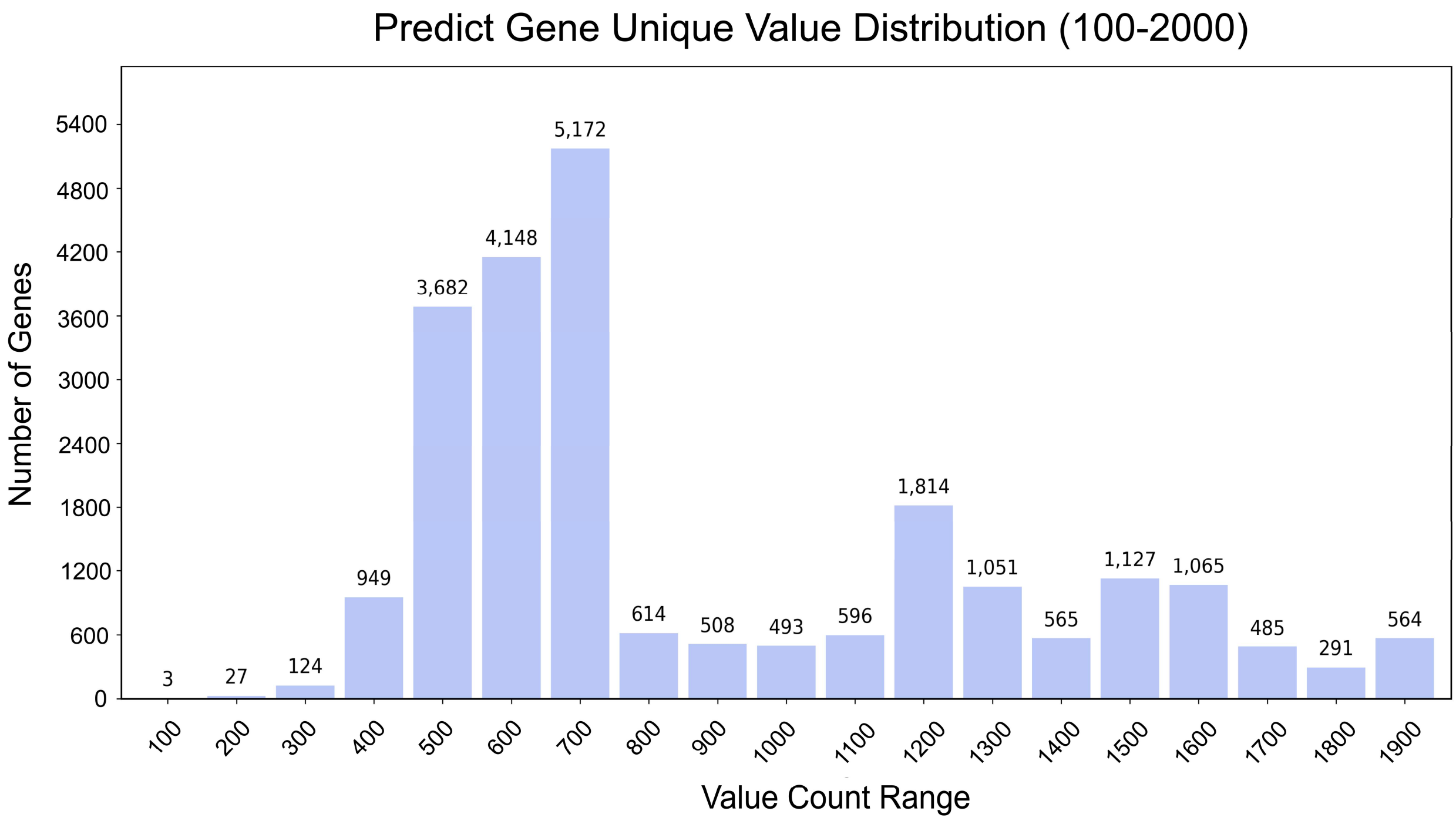

Fig. S11. Distribution of unique predicted expression values per gene across all spots in the test set. Most genes assume hundreds to thousands of distinct values, indicating high-dynamic-range, gene-specific decoding rather than collapse to a generic "pan-cellular" signal.

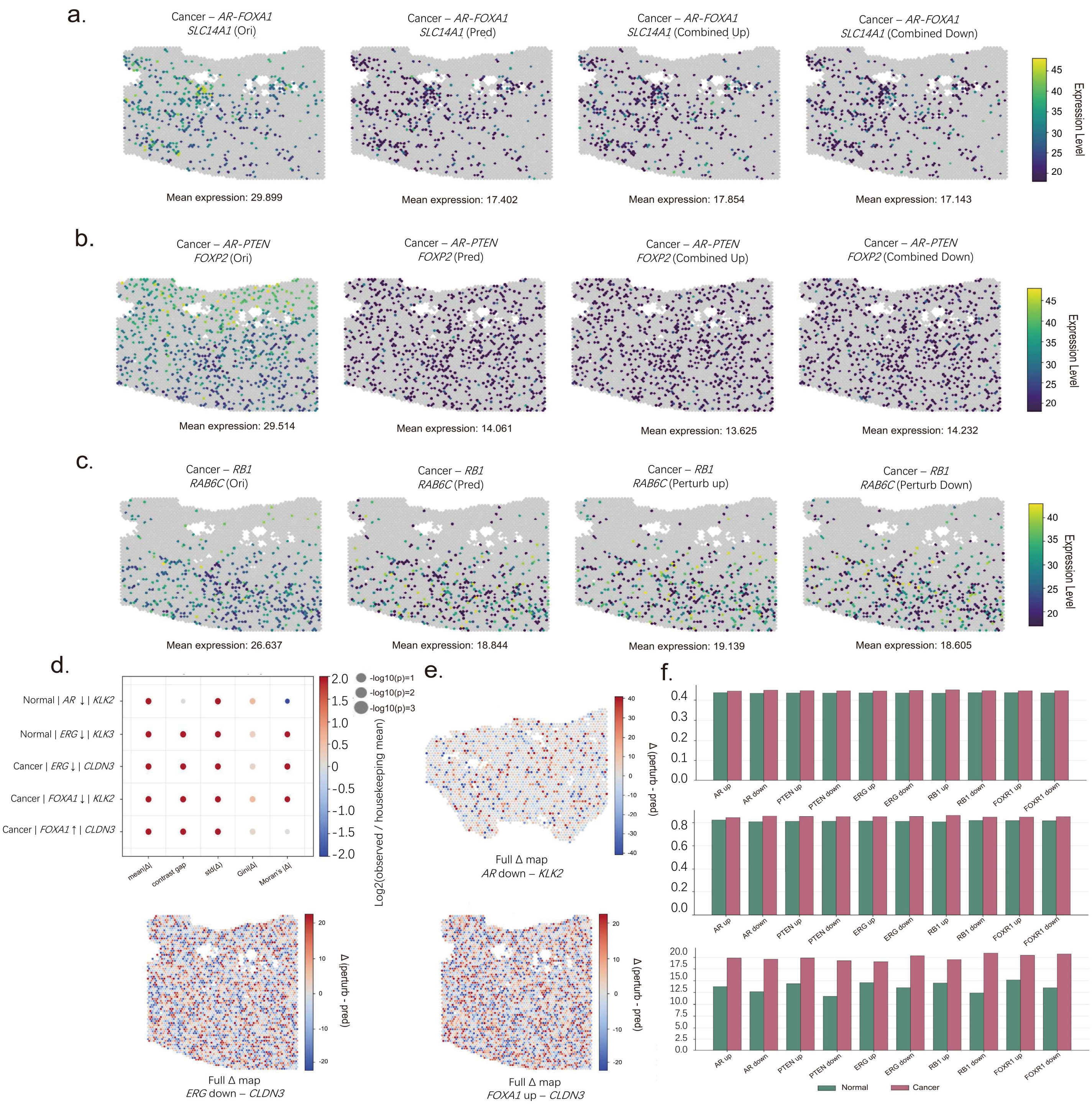

Fig. S12 | Representative spatial perturbation (image-only) and evaluation.

a, Columns are, from left to right: Ori (measured), Pred (unperturbed), and CLS-driven expression after simulated up-/down-regulation (Combined-Up / Combined-Down) for the specified axis (*AR–FOXA1*). Colors denote per-spot expression level. The direction of change for the genes (*SLC14A1*) is concordant with prior studies.

b, Columns are, from left to right: Ori (measured), Pred (unperturbed), and CLS-driven expression after simulated up-/down-regulation (Combined-Up / Combined-Down) for the specified axis (*AR–PTEN*). Colors denote per-spot expression level. The direction of change for the genes (*FOXP2*) is concordant with prior studies.

c, Columns are, from left to right: Ori (measured), Pred (unperturbed), and CLS-driven expression after simulated up-/down-regulation (Up / Down) for the specified gene (*RB1*). Colors denote per-spot expression level. The direction of change for the genes (*RAB6C*) is concordant with prior studies.

d, Displayed perturbation targets compared with housekeeping controls across perturbation-structure metrics. For each displayed case, the target gene was compared against a predefined housekeeping-gene panel under the same perturbation condition. Dot colour indicates the log2 ratio between the observed target-gene value and the housekeeping-gene background mean, and dot size indicates empirical significance derived from the housekeeping background distribution. The perturbation-structure metrics shown are mean|Δ|, contrast gap, std(Δ), Gini|Δ|, and Moran's I of |Δ| (see Methods). Here, Δ = (perturbed prediction − baseline prediction).

e, Additional full spatial perturbation maps for supplementary single-perturbation examples. Top, Normal | *AR* down -> *KLK2*. Bottom, Cancer | *FOXA1* up -> *CLDN3*. Left, Cancer | *ERG* down -> *CLDN3*. Colour indicates the signed perturbation response, defined as Δ = (perturbed prediction – baseline prediction). These maps are shown to provide full-field context for examples that are spatially informative but directionally less clean than the main displayed single-perturbation cases.

f, Perturbation response statistics across cohorts. mean_var_delta—the average across genes of the variance of the per-spot change between the perturbed and unperturbed predictions; mean_cv_absolute—the average across genes of the coefficient of variation computed on the absolute per-spot changes, i.e., spread relative to the mean effect size; mean_gini_absolute—the average across genes of the Gini coefficient of the absolute per-spot changes, reflecting how unevenly the effect is distributed in space. Consistent separation across metrics indicates non-collapsed, non-linear, gene-specific decoding and biologically plausible heterogeneity under image-only simulation.

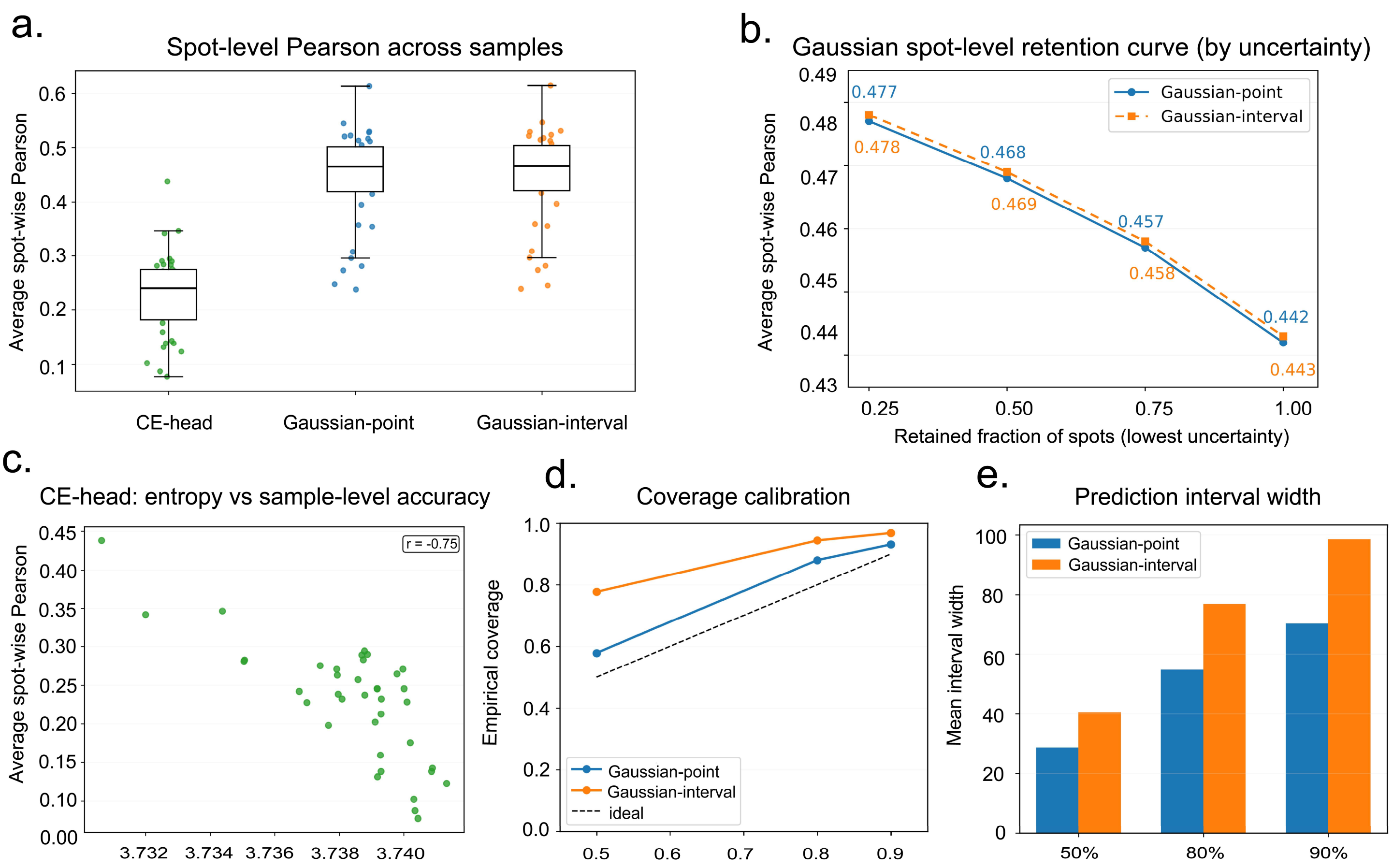

Fig. S13 | Supplementary analysis of uncertainty.

a, Average spot-wise Pearson correlation across samples for three uncertainty formulations: CE-head entropy baseline, point-wise Gaussian, and interval-aware Gaussian. Each dot represents one sample; boxplots show the median and interquartile range, with whiskers extending to 1.5 × interquartile range (IQR). Both Gaussian formulations substantially outperform the CE-head baseline, whereas the point-wise and interval-aware Gaussian models show highly similar spot-level predictive fidelity across samples.

b, Average spot-wise Pearson correlation after retaining only the lowest-uncertainty 25%, 50%, 75%, or all spots for the point-wise Gaussian and interval-aware Gaussian formulations. In both models, restricting analysis to lower-uncertainty spots consistently enriches for higher-fidelity regions, indicating that predicted uncertainty can be used directly to rank spatial locations by reliability. The two Gaussian formulations show nearly identical retention behavior across the full range of retained fractions.

c, Relationship between mean CE-head entropy and average spot-wise Pearson correlation across samples. Each dot represents one sample. Higher entropy is associated with lower predictive accuracy (Pearson $r = -0.75$), indicating that CE-head entropy provides an informative confidence proxy despite the substantially weaker predictive fidelity of the CE-head formulation relative to the Gaussian-based models.

d, Coverage calibration under Gaussian uncertainty formulations. Empirical coverage as a function of nominal coverage (50%, 80%, and 90%) for the point-wise Gaussian and interval-aware Gaussian formulations. The dashed line indicates ideal calibration. The interval-aware Gaussian achieves higher empirical coverage across all nominal levels, consistent with a more conservative uncertainty profile.

e, Prediction interval width under Gaussian uncertainty formulations. Mean prediction interval width at nominal coverage levels of 50%, 80%, and 90% for the point-wise Gaussian and interval-aware Gaussian formulations. The interval-aware Gaussian produces consistently wider intervals, whereas the point-wise Gaussian yields sharper and more compact uncertainty estimates

a.

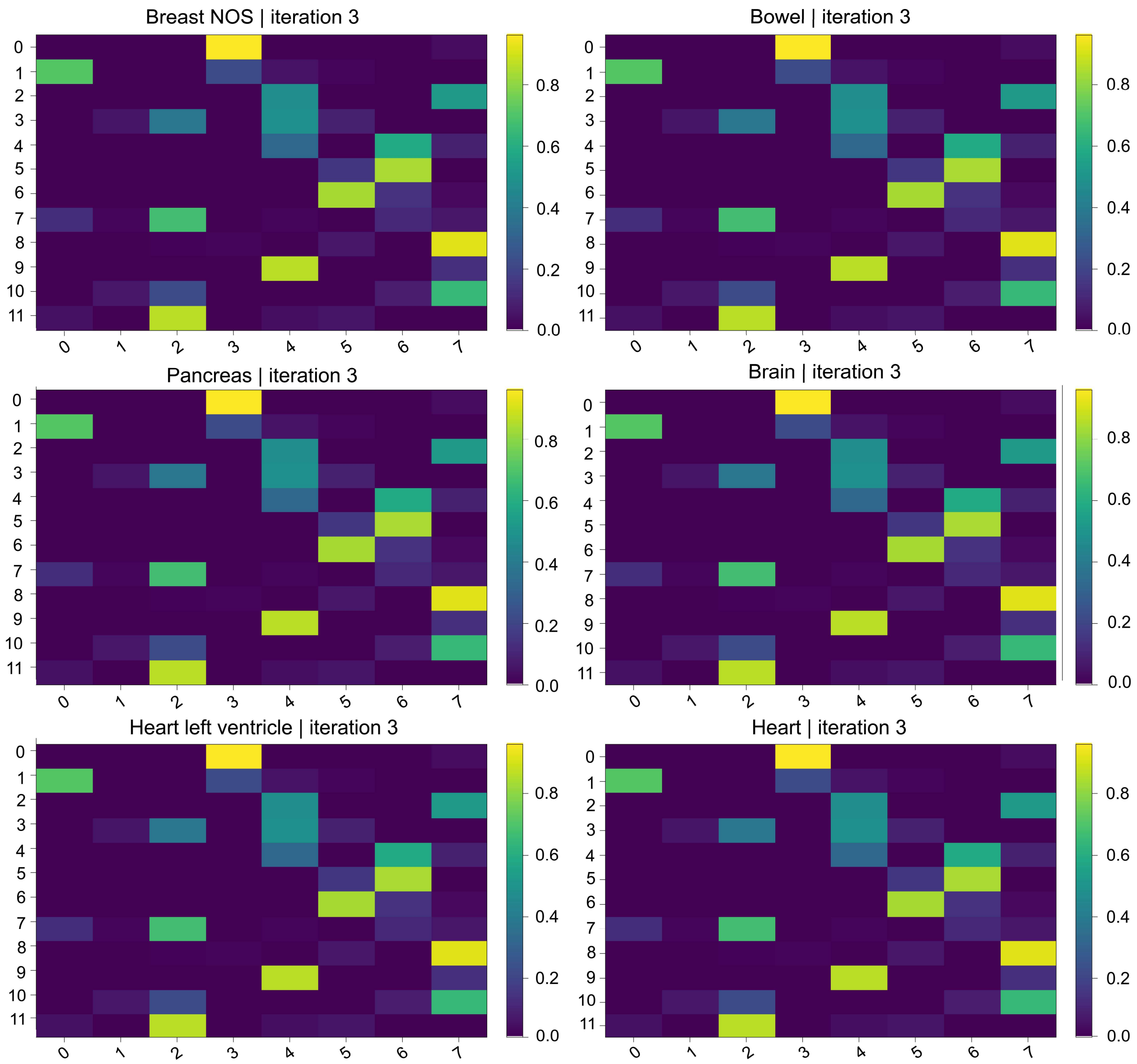

Fig. S14 | Tissue-stratified expert-routing heatmaps at the final Coconut iteration.

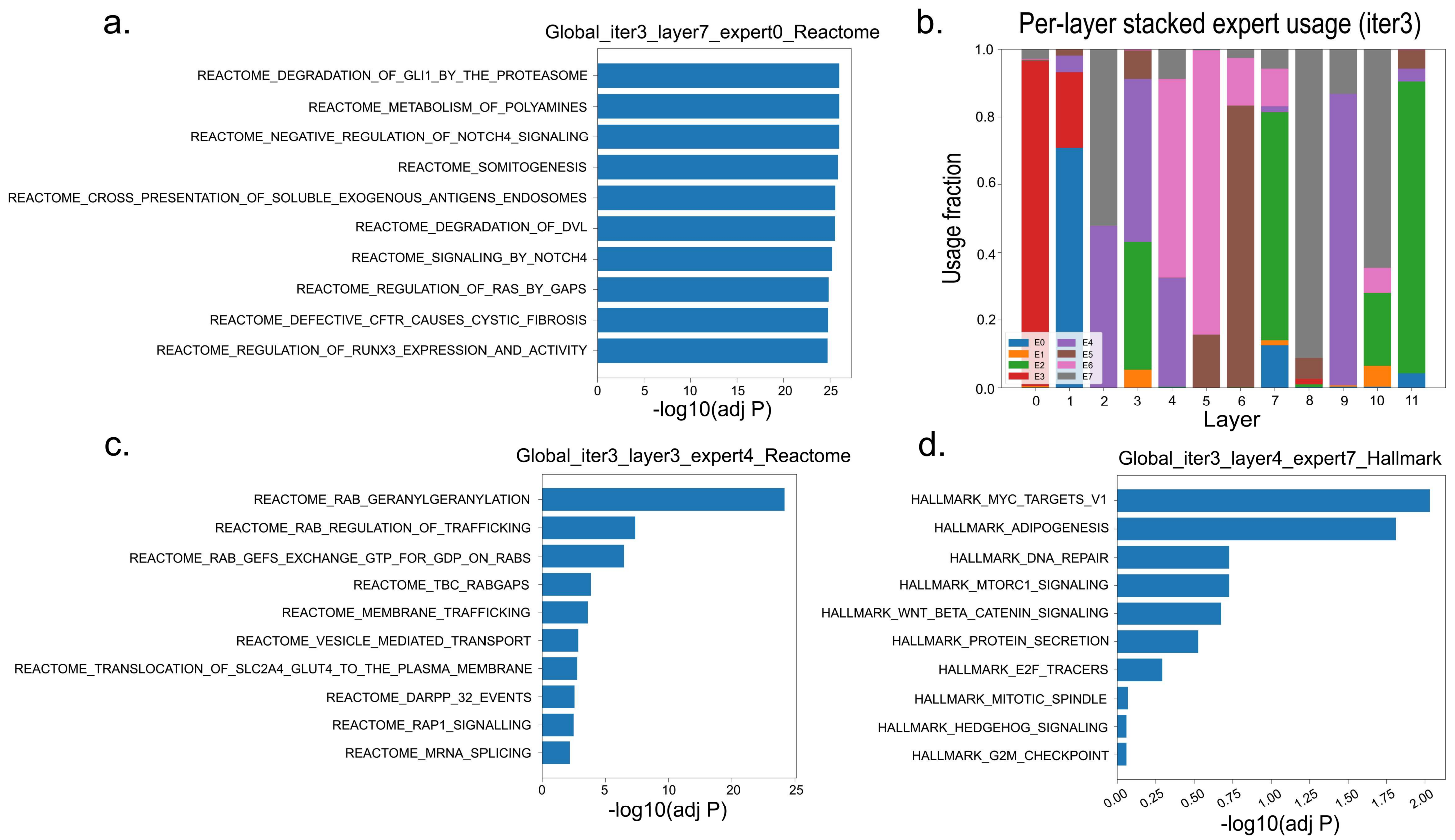

Fig. S15 | Representative expert-associated programs and expert-usage composition at the final Coconut iteration.

a, Top Reactome pathways enriched among genes most frequently routed to layer 7, expert 0 at iteration 3, highlighting a proteostasis / antigen-processing-associated expert program.

b, Per-layer stacked expert-usage fractions at iteration 3. Each bar represents one decoder layer, and colours denote the fraction of valid gene tokens routed to each expert, showing that expert usage is non-uniform across layers and that different layers exhibit distinct routed-expert compositions.

c, Top Reactome pathways enriched among genes most frequently routed to layer 3, expert 4 at iteration 3, highlighting membrane trafficking and RAB geranylgeranylation-related programs.

d, Top Hallmark pathways enriched among genes most frequently routed to layer 4, expert 7 at iteration 3, highlighting MYC- and signaling-related regulatory programs

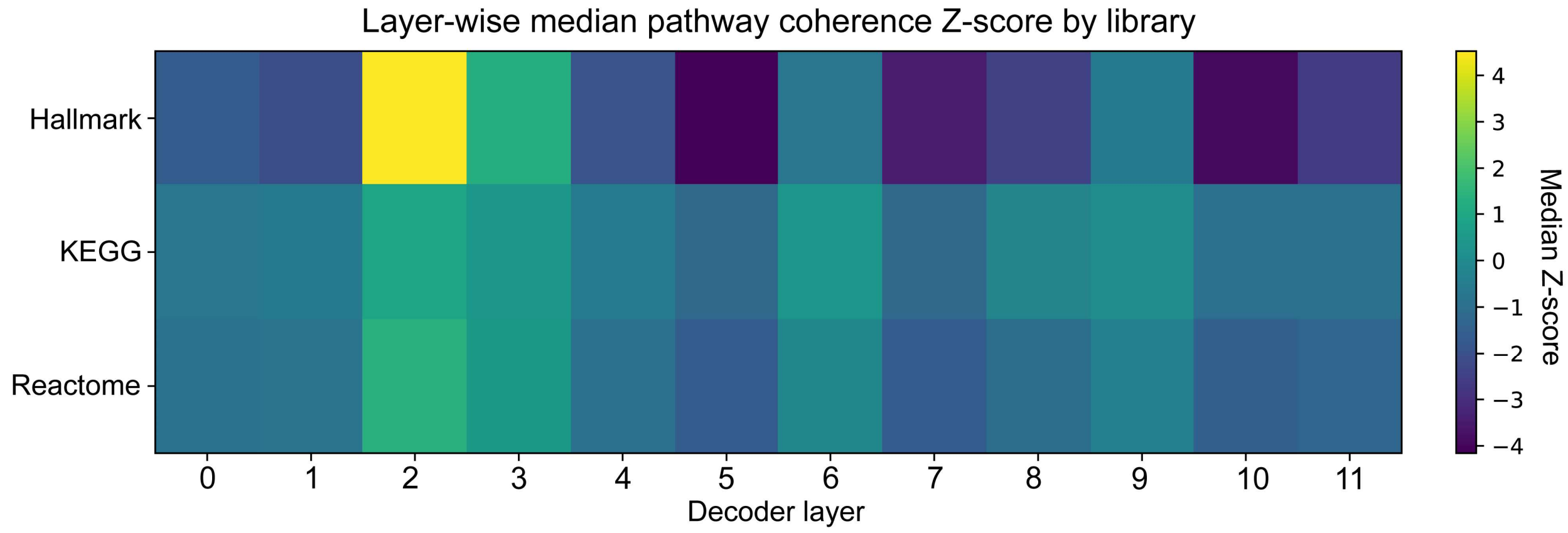

Fig. S16 | Layer-wise median pathway-coherence Z-score by pathway library.

The heatmap summarizes median pathway-coherence Z-scores across decoder layers for Hallmark, KEGG, and Reactome pathway libraries. Warmer colors indicate higher coherence relative to the matched empirical null, whereas cooler colors indicate weaker or negative coherence. Layer 2 showed the strongest positive median coherence across libraries.

Supplementary Tables

Table S1: Hyperparameters used in Coladan stage 1 training

Hyperparameter	Value
Automatic mixed precision	FP16
Batch size	512
Warm ratio	0.4
Mask ratio	30-50%
Loss	0.8 NLL + 0.2 MoE load balancing
lr	0.0001
Max length	5,000
Optimizer	Adam
Data process	Bin50
Gradient accumulation steps	4
Learning rate scheduler	Cosine
Epoch	20

Batch size refers to the total batch size across GPUs. Effect batch size used for optimization is batch size*gradient accumulation steps. Max length denotes the maximum number of gene tokens per spot. Within each batch, all spot inputs are padded to this length—equal to the gene count of the spot with the most genes—by filling any remaining positions with <pad>.

Table S2: UNI2 fine-tuning hyperparameters

Hyperparameter	Value
Prediction target	2500 genes
fine-tuned	only projector
sample batch per gpu	8
lr	2×10^{-5}
Projector	Linear(1536→2048) → LayerNorm → LeakyReLU → Linear(2048→2048) → LeakyReLU → Linear(2048→2500)
warm-up	Cosine with warmup
warm-up steps	30 samples
AMP	fp16（torch.cuda.amp.GradScaler）
epochs	5
optimizer	Adam
data process	Bin50
extract and predict center	224*224px for 64*64px

Table S3: CONCH v1.5 fine-tuning hyperparameters

Hyperparameter	Value
Prediction target	2500 genes
fine-tuned	only projector
sample batch per GPU	8
lr	2×10^{-5}
Projector	Linear(768→2048) → LayerNorm → LeakyReLU → Linear(2048→2048) → LeakyReLU → Linear(2048→2500)
warm-up	Cosine with warmup
warm-up steps	30 samples
AMP	fp16（torch.cuda.amp.GradScaler）
epochs	5
optimizer	Adam
data process	Bin50
extract and predict center	224*224px for 64*64px

Table S4: MUSK fine-tuning hyperparameters

Hyperparameter	Value
Prediction target	2500 genes
fine-tuned	only projector
sample batch per GPU	8
lr	2×10^{-5}
Projector	Linear(1536→2048) → LayerNorm → LeakyReLU → Linear(2048→2048) → LeakyReLU → Linear(2048→2500)
warm-up	Cosine with warmup
warm-up steps	30 samples
AMP	fp16（torch.cuda.amp.GradScaler）
epochs	5
optimizer	Adam
data process	Bin50
extract and predict center	384*384px for 64*64px

Table S5: H-Optimus-0 fine-tuning hyperparameters

Hyperparameter	Value
Prediction target	2500 genes
fine-tuned	only projector
sample batch per GPU	8
lr	2×10^{-5}
Projector	Linear(1536→2048) → LayerNorm → LeakyReLU → Linear(2048→2048) → LeakyReLU → Linear(2048→2500)
warm-up	Cosine with warmup
warm-up steps	30 samples
AMP	fp16（torch.cuda.amp.GradScaler）
epochs	5
optimizer	Adam
data process	Bin50
extract and predict center	384*384px for 64*64px

Table S6: TITAN fine-tuning hyperparameters

Hyperparameter	Value
Prediction target	2500 genes
fine-tuned	projector and last three vision encoder blocks
sample batch per GPU	8
lr	2×10^{-5}
Projector	Linear(768→2048) → LayerNorm → LeakyReLU → Linear(2048→2048) → LeakyReLU → Linear(2048→2500)
warm-up	Cosine with warmup
warm-up steps	30 samples
AMP	fp16（torch.cuda.amp.GradScaler）
epochs	5
optimizer	Adam
data process	Bin50
extract and predict center	384*384px for 64*64px

Table S7: Virchow fine-tuning hyperparameters

Hyperparameter	Value
Prediction target	2500 genes
fine-tuned	only projector
sample batch per GPU	8
lr	2×10^{-5}
Projector	Linear(1280→2048) → LayerNorm → LeakyReLU → Linear(2048→2048) → LeakyReLU → Linear(2048→2500)
warm-up	Cosine with warmup
warm-up steps	30 samples
AMP	fp16（torch.cuda.amp.GradScaler）
epochs	5
optimizer	Adam
data process	Bin50
extract and predict center	224*224px for 64*64px

Table S8: GigaPath fine-tuning hyperparameters

Hyperparameter	Value
Prediction target	2500 genes
fine-tuned	only projector
sample batch per GPU	8
lr	2×10^{-5}
Projector	Linear(1536→2048) → LayerNorm → LeakyReLU → Linear(2048→2048) → LeakyReLU → Linear(2048→2500)
warm-up	Cosine with warmup
warm-up steps	30 samples
AMP	fp16（torch.cuda.amp.GradScaler）
epochs	5
optimizer	Adam
data process	Bin50
extract and predict center	384*384px for 64*64px

Table S9: Layer-wise MoE pathway-enrichment summaries

layer	library	pathways	Mean delta	Median delta	Mean Z score	Median Z score	fraction_empirical_p <0.05
0	Hallmark	50	-0.073	-0.053	-2.306	-1.616	0.02
0	KEGG	358	-0.061	-0.066	-0.714	-0.717	0
0	Reactome	1231	-0.058	-0.053	-1.008	-0.845	0.005
1	Hallmark	50	-0.066	-0.097	-1.516	-2.062	0.1
1	KEGG	358	-0.046	-0.084	-0.345	-0.608	0.061
1	Reactome	1231	-0.046	-0.077	-0.598	-0.829	0.059
2	Hallmark	50	0.053	0.035	6.768	4.523	0.68
2	KEGG	358	0.082	0.053	1.694	0.922	0.282
2	Reactome	1231	0.065	0.031	3.539	1.354	0.382
3	Hallmark	50	0.037	0.035	1.434	1.235	0.42
3	KEGG	358	0.064	0.027	0.758	0.431	0.235
3	Reactome	1231	0.045	0.024	0.959	0.512	0.271
4	Hallmark	50	-0.046	-0.052	-1.482	-1.905	0.08
4	KEGG	358	-0.03	-0.05	-0.323	-0.527	0.034
4	Reactome	1231	-0.037	-0.064	-0.741	-0.925	0.064
5	Hallmark	50	-0.157	-0.185	-3.487	-4.159	0.04
5	KEGG	358	-0.116	-0.156	-0.899	-1.175	0.014
5	Reactome	1231	-0.12	-0.167	-1.595	-1.635	0.024
6	Hallmark	50	-0.037	-0.031	-0.761	-0.751	0.08
6	KEGG	358	0.024	0.044	0.162	0.414	0.022
6	Reactome	1231	-0.007	-0.01	-0.07	-0.112	0.074
7	Hallmark	50	-0.139	-0.175	-2.912	-3.431	0.06
7	KEGG	358	-0.121	-0.149	-0.866	-1.161	0.031
7	Reactome	1231	-0.12	-0.172	-1.424	-1.626	0.048
8	Hallmark	50	-0.104	-0.122	-2.302	-2.47	0.06
8	KEGG	358	-0.044	-0.03	-0.386	-0.215	0
8	Reactome	1231	-0.08	-0.085	-1.05	-1.012	0.011
9	Hallmark	50	-0.028	-0.032	-0.636	-0.601	0.06
9	KEGG	358	0.011	0.011	0.073	0.117	0.008
9	Reactome	1231	-0.029	-0.034	-0.377	-0.384	0.026
10	Hallmark	50	-0.138	-0.167	-3.229	-3.894	0.04
10	KEGG	358	-0.094	-0.116	-0.792	-0.997	0.028
10	Reactome	1231	-0.097	-0.144	-1.372	-1.472	0.054
11	Hallmark	50	-0.142	-0.153	-2.711	-2.628	0.04
11	KEGG	358	-0.141	-0.129	-1.013	-0.928	0.006
11	Reactome	1231	-0.13	-0.145	-1.557	-1.341	0.028

For each decoder layer and pathway library, the table summarizes the layer-wise MoE pathway-

enrichment diagnostic. n pathways tested denotes the number of pathways evaluated in each library. Mean delta and Median delta summarize the average and median enrichment difference relative to the matched empirical null. Mean z and Median z summarize the corresponding standardized enrichment statistics. Fraction empirical $P < 0.05$ denotes the fraction of tested pathways with empirical P values below 0.05. Positive delta or z values indicate stronger pathway enrichment relative to the matched empirical null, whereas negative values indicate weaker enrichment relative to the null.

Table S10: Benchmark result details (Pearson correlation coefficient)

sample	Coladan	UNI2	Virchow	TITAN	MUSK	H-Optimus-0	GigaPath	CONCH v1.5	BLEEP	DeepSpot	HisToGene
V1_Breast_Cancer_Block_A_Section_1	0.5062	0.1396	0.1185	0.1384	0.1225	0.1127	0.0960	0.1714	0.3462	0.2017	0.4073
Targeted_Visium_Human_Glioblastoma_Pan_Cancer	0.3366	0.1674	0.1586	0.2034	0.2106	0.2362	0.1489	0.2104	0.0533	-0.0898	0.013
Visium_FFPE_Human_Ovarian_Cancer	0.3453	0.1222	0.1257	0.1401	0.1086	0.1343	0.0928	0.1683	0.0715	0.0782	0.043
Visium_FFPE_Human_Intestinal_Cancer	0.3866	0.1200	0.1170	0.1362	0.1235	0.1450	0.0954	0.1633	0.075	0.0829	0.1108
CytAssist_FFPE_Human_Lung_Squamous_Cell_Carcinoma	0.3708	0.0981	0.0816	0.0923	0.0784	0.0749	0.0785	0.1097	0.0896	-0.1771	0.1232
Targeted_Visium_Human_Cerebellum_Neuroscience	0.1096	0.1617	0.1206	0.2191	0.2142	0.2111	0.1091	0.3194	0.1247	0.0331	0.1356
Visium_FFPE_Human_Normal_Prostate	0.4252	0.1060	0.1013	0.1169	0.0899	0.1174	0.072	0.1232	0.0798	-0.1966	0.0042
V1_Human_Heart	0.5713	0.1236	0.1125	0.1353	0.1100	0.1357	0.0900	0.1638	0.3174	0.2831	0.4205
V1_Breast_Cancer_Block_A_Section_2	0.5089	0.1432	0.1245	0.1235	0.1272	0.1174	0.0972	0.1774	0.3432	0.2501	0.4111
V1_Human_Invasive_Ductal_Carcinoma	0.5187	0.1183	0.1022	0.1456	0.1004	0.0938	0.0823	0.1409	0.3474	0.2761	0.312
Visium_FFPE_Human_Cervical_Cancer	0.41	0.1059	0.1121	0.1217	0.1125	0.1121	0.077	0.1562	0.0560	0.0982	0.0323
Parent_Visium_Human_ColorectalCancer	0.5372	0.1216	0.1026	0.1080	0.107	0.1127	0.0972	0.1464	0.3736	0.0138	0.4827
Parent_Visium_Human_OvarianCancer	0.518	0.1102	0.1099	0.1042	0.0904	0.1039	0.0863	0.1469	0.3629	0.2278	0.3485
V1_Breast_Cancer_Block_A_Section_1	0.5122	0.1451	0.1255	0.1244	0.1289	0.1156	0.0981	0.1773	0.3442	0.1986	0.4011
V1_Human_Heart	0.5725	0.1181	0.1094	0.1366	0.1102	0.1378	0.0888	0.1651	0.3021	0.2679	0.4255
V1_Human_Brain_Section_2	0.3911	0.1079	0.0880	0.1378	0.0952	0.0986	0.0851	0.1450	0.2396	0.0005	0.1641
Parent_Visium_Human_Glioblastoma	0.4885	0.1215	0.1173	0.1502	0.1144	0.1320	0.0982	0.1579	0.3325	0.1142	0.366
V1_Human_Lymph_Node	0.4881	0.1154	0.1183	0.1254	0.1004	0.1096	0.0898	0.1564	0.3435	0.1045	0.4475
Parent_Visium_Human_Cerebellum	0.4015	0.1200	0.1087	0.1330	0.1180	0.1188	0.0922	0.1626	0.2654	0.2654	0.3618
V1_Human_Brain_Section_1	0.3913	0.1069	0.0852	0.1325	0.0902	0.0946	0.0820	0.1400	0.2358	-0.0082	0.1603
Visium_FFPE_Human_Breast_Cancer	0.4228	0.1071	0.1192	0.1267	0.1104	0.1177	0.0810	0.1485	0.0993	-0.1239	0.0145
Visium_FFPE_Human_Prostate_IF	0.4342	0.1218	0.1162	0.1327	0.1010	0.1377	0.0816	0.1493	0.0881	-0.1865	0.0498
Visium_FFPE_Human_Prostate_Cancer	0.4168	0.1142	0.1089	0.1226	0.1004	0.1262	0.0818	0.1416	0.0719	-0.2489	0.0028
Targeted_Visium_Human_BreastCancer_Immunology	0.4397	0.2763	0.2318	0.1894	0.3155	0.3628	0.2335	0.285	0.1441	0.1969	0.2023
V1_Breast_Cancer_Block_A_Section_2	0.5034	0.1395	0.1207	0.1204	0.1250	0.1113	0.0977	0.1724	0.3454	0.2457	0.4031
Visium_FFPE_Human_Prostate_Acinar_Cell_Carcinoma	0.3254	0.1268	0.1196	0.1603	0.1081	0.1390	0.0944	0.162	0.0750	0.1953	0.0219
Targeted_Visium_Human_OvarianCancer_Immunology	0.3649	0.2300	0.2331	0.1968	0.2383	0.3184	0.1943	0.2497	0.1420	0.1419	0.1814
Targeted_Visium_Human_ColorectalCancer_GeneSignature	0.193	0.1352	0.1205	0.1612	0.0977	0.1300	0.0530	0.1725	0.0277	-0.0556	0.0196
Visium_FFPE_Human_Prostate_IF	0.4346	0.1230	0.1131	0.1324	0.1011	0.1373	0.0828	0.1490	0.0872	-0.1812	0.0539
V1_Human_Lymph_Node	0.4291	0.1225	0.1220	0.1489	0.1078	0.1106	0.0894	0.1603	0.3431	0.1103	0.4627
Parent_Visium_Human_BreastCancer	0.5156	0.1293	0.1234	0.1259	0.1232	0.1237	0.0904	0.1528	0.3420	0.209246	0.3623
Visium_Human_Breast_Cancer	0.5235	0.1315	0.1208	0.1204	0.1207	0.1092	0.0921	0.1637	0.3652	0.198574	0.4344

Table S11: Benchmark result details (Spearman correlation coefficient)

sample	Coladan	UNI2	Virchow	TITAN	MUSK	H-Optimus-0	GigaPath	CONCH v1.5	BLEEP	DeepSpot	HisToGene
V1_Breast_Cancer_Block_A_Section_1	0.3468	0.1243	0.1086	0.1656	0.0928	0.0957	0.0674	0.1537	0.2374	0.0941	0.2867
Targeted_Visium_Human_Glioblastoma_Pan_Cancer	0.2653	0.0817	0.0954	0.1327	0.1335	0.1806	0.056	0.115	0.0459	-0.023	0.0243
Visium_FFPE_Human_Ovarian_Cancer	0.2615	0.1139	0.1188	0.1537	0.0868	0.1182	0.067	0.148	0.1599	0.1855	0.0964
Visium_FFPE_Human_Intestinal_Cancer	0.2913	0.1128	0.1071	0.1334	0.1021	0.1319	0.0694	0.1383	0.1874	0.2023	0.0975
CytAssist_FFPE_Human_Lung_Squamous_Cell_Carcinoma	0.3034	0.0839	0.0588	0.0813	0.0525	0.0623	0.0467	0.0855	0.0574	-0.1556	0.0132
Targeted_Visium_Human_Cerebellum_Neuroscience	0.0847	0.111	0.0483	0.1874	0.1837	0.1571	0.0149	0.3094	0.0705	0.0952	0.1098
Visium_FFPE_Human_Normal_Prostate	0.2785	0.113	0.1039	0.1344	0.0793	0.1168	0.0582	0.1183	0.1418	-0.198	0.0594
V1_Human_Heart	0.413	0.1054	0.1037	0.1365	0.0767	0.1235	0.0613	0.1615	0.2812	0.1914	0.2761
V1_Breast_Cancer_Block_A_Section_2	0.3469	0.129	0.1155	0.1181	0.0974	0.0999	0.066	0.1574	0.1723	0.1364	0.3164
V1_Human_Invasive_Ductal_Carcinoma	0.3255	0.1093	0.0925	0.1078	0.072	0.0828	0.0555	0.1256	0.2366	0.267	0.2814
Visium_FFPE_Human_Cervical_Cancer	0.31	0.0965	0.0984	0.1173	0.0883	0.0944	0.0452	0.139	0.1474	0.1809	0.0418
Parent_Visium_Human_ColorectalCancer	0.3451	0.1075	0.0947	0.1066	0.0829	0.1012	0.0769	0.1319	0.1983	0.0719	0.2572
Parent_Visium_Human_OvarianCancer	0.3253	0.0972	0.1071	0.1025	0.0602	0.0889	0.0536	0.1296	0.232	0.1907	0.2686
V1_Breast_Cancer_Block_A_Section_1	0.3557	0.1307	0.1147	0.1187	0.0981	0.0991	0.0659	0.1574	0.3649	0.0858	0.2514
V1_Human_Heart	0.4127	0.1001	0.1019	0.138	0.0814	0.1269	0.0609	0.1622	0.2648	0.1828	0.2727
V1_Human_Brain_Section_2	0.2071	0.0926	0.066	0.1086	0.071	0.0824	0.0596	0.1365	0.1987	0.0327	0.1428
Parent_Visium_Human_Glioblastoma	0.3042	0.1055	0.1101	0.1479	0.0892	0.1232	0.0745	0.1403	0.2525	0.1104	0.3078
V1_Human_Lymph_Node	0.2989	0.1004	0.1069	0.1215	0.0727	0.0938	0.0612	0.1323	0.0862	0.0774	0.3276
Parent_Visium_Human_Cerebellum	0.2518	0.1005	0.0952	0.1377	0.0913	0.1023	0.0607	0.152	0.2181	0.2213	0.2225
V1_Human_Brain_Section_1	0.2043	0.0916	0.0635	0.089	0.0663	0.0773	0.0564	0.1303	0.1913	0.0333	0.1365
Visium_FFPE_Human_Breast_Cancer	0.2926	0.1035	0.1177	0.1065	0.0949	0.1068	0.0624	0.1357	0.1587	-0.181	0.0217
Visium_FFPE_Human_Prostate_IF	0.2933	0.1205	0.1173	0.1428	0.0838	0.1333	0.061	0.1399	0.1684	-0.1304	0.0819
Visium_FFPE_Human_Prostate_Cancer	0.2801	0.1111	0.1099	0.1281	0.083	0.1227	0.0616	0.1283	0.1422	-0.3045	0.0459
Targeted_Visium_Human_BreastCancer_Immunology	0.3799	0.1688	0.116	0.098	0.212	0.307	0.1087	0.184	0.1171	0.1089	0.1294
V1_Breast_Cancer_Block_A_Section_2	0.3394	0.1265	0.1121	0.1163	0.0975	0.0949	0.0683	0.1551	0.3734	0.1262	0.2996
Visium_FFPE_Human_Prostate_Acinar_Cell_Carcinoma	0.2332	0.1178	0.1184	0.12	0.0816	0.127	0.0692	0.1449	0.1641	0.2923	0.0562
Targeted_Visium_Human_OvarianCancer_Immunology	0.2801	0.1532	0.1304	0.1013	0.1085	0.2665	0.068	0.1301	0.113	0.0576	0.1189
Targeted_Visium_Human_ColorectalCancer_GeneSignature	0.1435	0.0847	0.0802	0.1436	0.0311	0.083	-0.0207	0.1376	0.0115	0.002	-0.0118
Visium_FFPE_Human_Prostate_IF	0.2933	0.1221	0.1142	0.1424	0.084	0.1325	0.0622	0.1398	0.1457	-0.1304	0.0863
V1_Human_Lymph_Node	0.2704	0.1059	0.1076	0.1572	0.0782	0.0939	0.0589	0.1329	0.2812	0.0718	0.3101
Parent_Visium_Human_BreastCancer	0.3523	0.1102	0.1091	0.1185	0.0876	0.1084	0.0577	0.1255	0.1891	0.0807	0.2479
Visium_Human_Breast_Cancer	0.3517	0.1204	0.1133	0.1188	0.0921	0.0921	0.0627	0.1473	0.207	0.1156	0.2427

Table S12: Per-compartment mean signed Δ values

case_label	driver	direction	Target gene	Sample kind	Epithelial Mean delta	Tumor Enriched Mean delta	Stroma Mean delta	Immune Mean delta	Others Mean delta
Normal | *AR*↓ | *KLK2*	*AR*	down	*KLK2*	normal	-0.07301	-	1.029062	0.202382	-0.30144
Normal | *ERG*↓ | *KLK3*	*ERG*	down	*KLK3*	normal	-0.12855	-	0.482366	1.099966	0.456905
Cancer | *ERG*↓ | *CLDN3*	*ERG*	down	*CLDN3*	cancer		-0.23777	-0.45585	-0.20598	0.007084
Cancer | *FOXA1*↑ | *KLK2*	*FOXA1*	up	*KLK2*	cancer	-	0.423831	1.136713	1.272971	-0.25335
Cancer | *FOXA1*↓ | *CLDN3*	*FOXA1*	down	*CLDN3*	cancer	-	-0.10443	-0.48008	0.367788	0.676991

For each displayed perturbation case, the table reports the mean signed **Δ** value within each coarse compartment. For normal prostate sections, compartments are **epithelial**, **stroma**, **immune** and **others**; for prostate cancer sections, compartments are **tumour-enriched**, **stroma**, **immune** and **others**. Positive values indicate a higher predicted expression under the perturbation condition relative to baseline, whereas negative values indicate a lower predicted expression. Here, **Δ = (perturbed prediction − baseline prediction)**.

Table S13: Matched empirical-null comparison

case_label	Normal | AR↓ | KLK2	Normal | ERG↓ | KLK3	Cancer | ERG↓ | CLDN3	Cancer | *FOXA1*↑ | *KLK2*	Cancer | *FOXA1*↓ | *CLDN3*
mean_abs_delta__observed	8.056245573	10.33700661	8.69837422	7.640997304	8.751122455
mean_abs_delta__null_mean	6.069661325	5.911932445	5.52236085	6.428741582	5.526555778
mean_abs_delta__obs_minus_null	1.986584248	4.425074166	3.17601337	1.212255722	3.224566677
mean_abs_delta__p_empirical	0.337932414	0.079384123	0.14917016	0.359328134	0.153769246
contrast_gap_abs__observed	-0.172278111	0.232148911	0.44973905	0.367275503	0.271929808
contrast_gap_abs__null_mean	0.010725221	0.000270042	0.00814070	0.02263826	0.002294695
contrast_gap_abs__obs_minus_nul	-0.183003332	0.231878869	0.44159834	0.344637243	0.269635113
contrast_gap_abs__p_empirical	0.779844031	0.215956809	0.01299740	0.093581284	0.054389122
std_delta__observed	13.50215632	14.09116709	11.3853007	12.95028199	11.42584571
std_delta__null_mean	8.413721165	8.106385523	7.48649529	9.084366213	7.411194563
std_delta__obs_minus_null	5.088435154	5.984781569	3.89880544	3.865915773	4.014651151
std_delta__p_empirical	0.101579684	0.088982204	0.19956008	0.108178364	0.200159968
gini_absdelta__observed	0.628905251	0.503459028	0.47373756	0.631403209	0.470452994
gini_absdelta__null_mean	0.486959206	0.482923252	0.47223730	0.500935414	0.468862703
gini_absdelta__obs_minus_null	0.141946046	0.020535776	0.00150026	0.130467795	0.001590291
gini_absdelta__p_empirical	0.083383323	0.319136173	0.35772845	0.085982803	0.353129374
moranI_abs_delta__observed	-0.007417139	0.014112325	0.01552388	0.006211144	-0.003961076
moranI_abs_delta__null_mean	-0.000140005	-0.00016126	-0.0000869	-0.00084299	0.000464474
moranI_abs_delta__obs_minus_nul	-0.007277133	0.014273594	0.01561076	0.00705414	-0.00442555
moranI_abs_delta__p_empirical	0.785442911	0.066586683	0.00439912	0.161367726	0.721655669

For each displayed perturbation case and each perturbation-structure metric, the table reports the observed target-gene value (observed), the mean of the matched empirical-null distribution (null_mean), the observed-minus-null difference (obs_minus_null), and the empirical P value (p_empirical). The perturbation-structure metrics are defined as follows: mean_abs_delta, mean of |Δ| across spots, summarizing overall perturbation magnitude; contrast_gap_abs, difference between mean |Δ| inside the displayed compartment and mean |Δ| outside that compartment, summarizing compartment-level contrast; std_delta, standard deviation of signed Δ across spots, summarizing overall spatial variation; gini_absdelta, Gini coefficient of |Δ|, quantifying how unevenly perturbation magnitude is distributed across the tissue; and moranI_abs_delta, Moran's I of |Δ|, quantifying spatial autocorrelation of perturbation magnitude. Here, **Δ = (perturbed prediction − baseline prediction)**.

Table S14: VisiumHD evaluation details (Pearson correlation coefficient)

sample	Coladan	TITAN	H-Optimus-0	CONCH v1.5
FF_Human_Breast_Cancer	0.3748	0.0514	0.0515	0.0463
FF_Human_Tonsil	0.3538	0.0433	0.0554	0.0405
Human_Breast_Cancer_Fixed_Frozen	0.3367	0.0366	0.0396	0.0341
Human_Breast_Cancer_Fresh_Frozen	0.3355	0.0338	0.0351	0.0153
Human_Colon_Cancer_P1	0.3359	0.047	0.0527	0.0345
Human_Colon_Cancer_P2	0.3649	0.0362	0.0448	0.0382
Human_Colon_Cancer_P5	0.3361	0.0436	0.0378	0.0212
Human_Colon_Cancer	0.366	0.0386	0.0427	0.0372
Human_Colon_Normal_P3	0.3583	0.0509	0.0425	0.0567
Human_Colon_Normal_P5	0.3331	0.0468	0.0334	0.0182
Human_Kidney_FFPE	0.2903	0.0352	0.0179	0.0319
Human_Lung_Cancer_Fixed_Frozen	0.358	0.041	0.0591	0.0394
Human_Lung_Cancer_HD_Only_Experiment1	0.3553	0.0459	0.0423	0.0268
Human_Lung_Cancer_HD_Only_Experiment2	0.3359	0.0477	0.0422	0.0325
Human_Lung_Cancer_post_Xenium_Prime_5K_Experiment2	0.2992	0.0479	0.0423	0.0261
Human_Lung_Cancer_post_Xenium_v1_Experiment1	0.3254	0.0383	0.0288	0.0194
Human_Pancreas	0.2875	0.0399	0.0076	0.036
Human_Tonsil_Fresh_Frozen	0.3505	0.0414	0.0536	0.0337

Table S15: VisiumHD evaluation details (Spearman correlation coefficient)

sample	Coladan	TITAN	H-Optimus-0	CONCH_v1.5
FF_Human_Breast_Cancer	0.2579	0.0648	0.0707	0.0464
FF_Human_Tonsil	0.2243	0.0540	0.0659	0.0361
Human_Breast_Cancer_Fixed_Frozen	0.2557	0.0597	0.0568	0.0395
Human_Breast_Cancer_Fresh_Frozen	0.2270	0.0428	0.0520	0.0119
Human_Colon_Cancer_P1	0.2770	0.0690	0.0939	0.0520
Human_Colon_Cancer_P2	0.2872	0.0573	0.0792	0.0465
Human_Colon_Cancer_P5	0.2614	0.0707	0.0703	0.0153
Human_Colon_Cancer	0.2876	0.0591	0.0780	0.0460
Human_Colon_Normal_P3	0.2948	0.0834	0.0787	0.0655
Human_Colon_Normal_P5	0.2446	0.0684	0.0539	0.0173
Human_Kidney_FFPE	0.2249	0.0534	0.0225	0.0177
Human_Lung_Cancer_Fixed_Frozen	0.3066	0.0548	0.0971	0.0450
Human_Lung_Cancer_HD_Only_Experiment1	0.2807	0.0763	0.0738	0.0358
Human_Lung_Cancer_HD_Only_Experiment2	0.2733	0.0779	0.0687	0.0399
Human_Lung_Cancer_post_Xenium_Prime_5K_Experiment2	0.2408	0.0768	0.0608	0.0266
Human_Lung_Cancer_post_Xenium_v1_Experiment1	0.2466	0.0662	0.0537	0.0233
Human_Pancreas	0.1373	0.0352	-0.0021	0.0303
Human_Tonsil_Fresh_Frozen	0.2536	0.0619	0.0852	0.0402

Table S16: Xenium evaluation details (Pearson correlation coefficient)

Sample	CONCH v1.5	TITAN	H-Optimus-0	Coladan
TENX158	0.149	0.1099	0.0694	0.379
NCBI882	0.1681	0.1716	0.1871	0.3589
NCBI856	0.063	0.1338	0.1881	0.2616
NCBI884	0.2183	0.1695	0.1784	0.3272
NCBI873	0.0968	0.1657	0.2161	0.247
NCBI866	0.0861	0.1692	0.1601	0.2762
TENX157	0.1154	0.0923	0.0459	0.3779
NCBI865	0.1978	0.2202	0.2601	0.4062
TENX147	0.1724	0.0736	0.1124	0.3862
NCBI857	0.0199	0.1327	0.1895	0.241
NCBI875	0.0689	0.127	0.1502	0.2174

Table S17: Xenium evaluation details (Spearman correlation coefficient)

Sample	CONCH v1.5	TITAN	H-Optimus-0	Coladan
TENX158	0.1411	0.1151	0.0899	0.3291
NCBI882	0.172	0.1961	0.1727	0.3485
NCBI856	0.061	0.1634	0.1661	0.2513
NCBI884	0.2214	0.1937	0.1532	0.3112
NCBI873	0.0979	0.1801	0.1955	0.2365
NCBI866	0.0869	0.175	0.1541	0.26
TENX157	0.1251	0.104	0.0699	0.3351
NCBI865	0.2009	0.2458	0.2299	0.3884
TENX147	0.1536	0.0632	0.0844	0.3929
NCBI857	0.0233	0.1721	0.1879	0.2371
NCBI875	0.0635	0.1355	0.1396	0.2095

Table S18: Hyperparameters used in Coladan stage 2.1 training

Hyperparameter	Value
Automatic mixed precision	FP16
Batch size	512
Warm ratio	0.4
Mask ratio	20-40%
Loss	0.8 NLL + 0.2 MoE load balancing
lr	0.0001
Max length	6,000
Optimizer	Adam
Gene expression process	Bin50
Image process	224*224 px for predict 64*64 px
Gradient accumulation steps	4
Learning rate scheduler	Cosine
Epoch	10

Stage 2.1 trains only the projector while the patch encoder, slide encoder, and gene decoder remain frozen. The batch size refers to the total number of spots processed across all GPUs in this stage.

Table S19: Hyperparameters used in Coladan stage 2.2 training

Hyperparameter	Value
Automatic mixed precision	FP16
Sample batch size	16
Cell batch size	128
Warm ratio	0.4
Mask ratio	20-40%
Loss	0.8 NLL + 0.2 MoE load balancing
lr	0.0001
Max length	6,000
Optimizer	Adam
Gene expression process	Bin50
Image process	224*224 px for predict 64*64 px
Gradient accumulation steps	4
Learning rate scheduler	Cosine
Epoch	10

Stage 2.2 unfreezes all model components for end-to-end training. In this phase, the sample batch size denotes the total number of samples per training step, while the cell batch size specifies the total number of spots processed across all GPUs in each iteration.
